# Light‐Driven *gem* Hydrogenation: An Orthogonal Entry into “Second‐Generation” Ruthenium Carbene Catalysts for Olefin Metathesis

**DOI:** 10.1002/chem.202101176

**Published:** 2021-05-01

**Authors:** Raphael J. Zachmann, Alois Fürstner

**Affiliations:** ^1^ Max-Planck-Institut für Kohlenforschung 45470 Mülheim/Ruhr Germany

**Keywords:** *gem* hydrogenation, Grubbs catalysts, metal carbenes, metathesis, ruthenium

## Abstract

The newly discovered light‐driven *gem* hydrogenation of alkynes opens an unconventional yet efficient entry into five‐coordinate Grubbs‐type ruthenium carbene complexes with *cis*‐disposed chloride ligands. Representatives of this class featuring a chelate substructure formed by an iodo‐substituted benzylidene unit react with (substituted) 2‐isopropoxystyrene to give prototypical “second‐generation” Grubbs‐Hoveyda complexes for olefin metathesis. The new approach to this venerable catalyst family is safe and versatile as it uses a triple bond rather than phenyldiazomethane as the ultimate carbene source and does not require any sacrificial phosphines.

## Graphical abstract

**On the spot**: Light‐driven *gem* hydrogenation elicits the inherent *vic*‐dicarbene character of a triple bond. This unusual reactivity mode can be harnessed in the form of a novel, safe, convenient and efficient approach to second‐generation “Grubbs‐Hoveyda”‐type ruthenium carbene complexes for olefin metathesis that relies on the use of alkynes rather than diazoalkanes as the ultimate carbene sources.

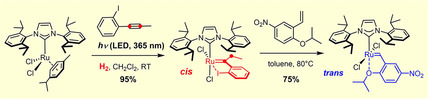

In a recent Communication, we reported that ruthenium complexes of the general type [(NHC)(η^6^‐cymene)RuCl_2_] (**1**) are able to effect the *geminal* hydrogenation of internal alkynes, provided that the reaction mixture is irradiated with UV‐A light (*λ*=370±40 nm).^[1]^ Delivery of both H‐atoms of H_2_ to the same C‐atom of the triple bond (“*gem* hydrogenation”) entails concomitant formation of a discrete metal carbene at the adjacent position (Scheme 1). Despite the immense literature on catalytic hydrogenation at large, this reactivity mode had been unknown until it was discovered during recent investigations into the mechanism of *trans* hydrogenation catalyzed by [Cp*RuCl]_4_ or related piano‐stool complexes;^[2**–**8]^ the light‐driven catalyst system represents only the second known example.^[1]^

**Scheme 1:**
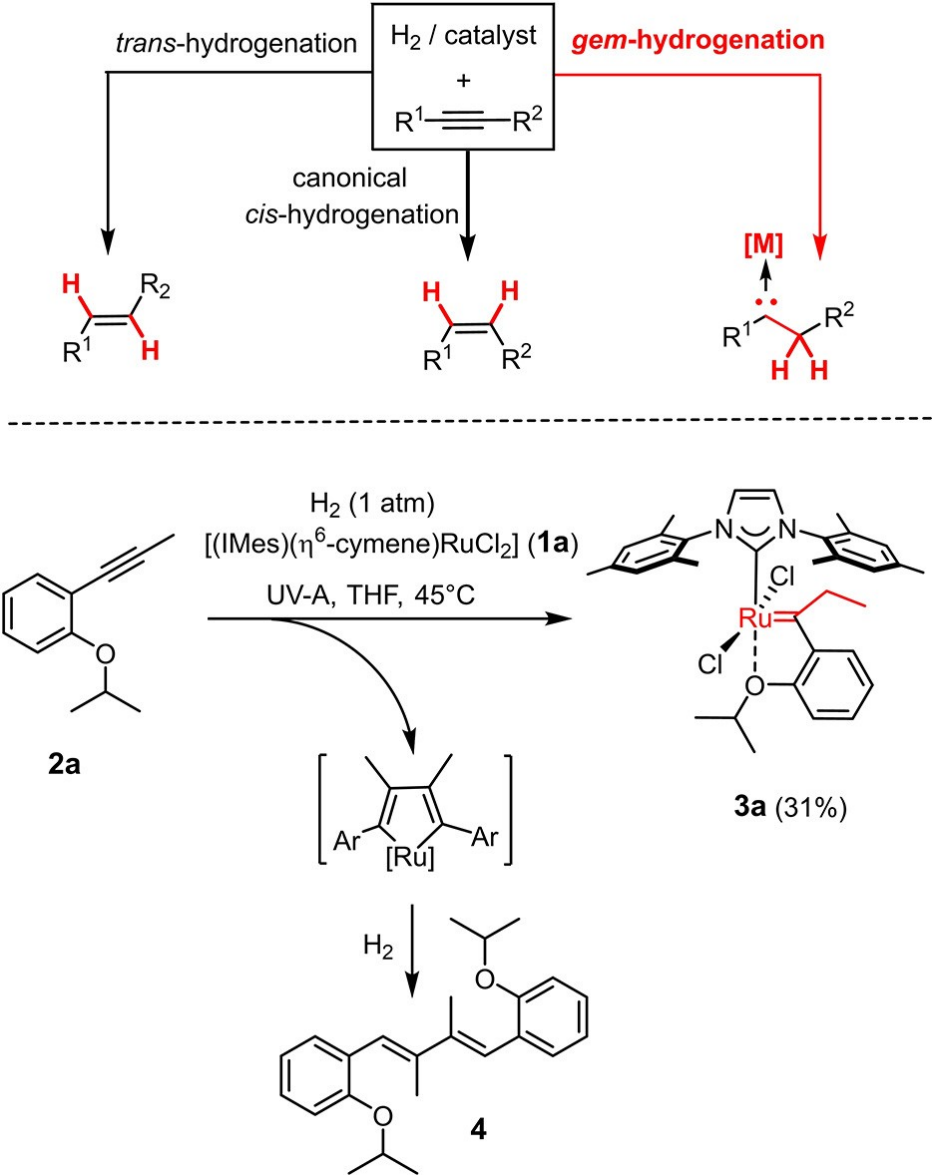
Concept of alkyne *gem* hydrogenation; first application to the direct formation of a Grubbs‐Hoveyda type catalyst;^[1]^ IMes=1,3‐dimesitylimidazol‐2‐ylidene.

For the particular ligand sphere of precatalyst **1**, a “second‐generation” Grubbs‐type ruthenium carbene is generated, thus opening an orthogonal and conceptually novel entry into metathesis.[^9^, ^10^, ^11^, ^12^, ^13^] Proof‐of‐concept notwithstanding, a number of shortcomings currently limit the scope of this transformation. This aspect becomes apparent from the fact that the light‐driven *gem* hydrogenation of 1‐isopropoxy‐2‐(prop‐1‐yn‐1‐yl)benzene (**2 a**) with [(IMes)(η^6^‐cymene)RuCl_2_] (**1 a**)[^1^, ^14^] furnished the expected complex **3 a** in only 31 % yield.^[1]^ Most of the substrate was consumed by competing dimerization with formation of diene **4**, supposedly via oxidative cyclization and subsequent hydrogenolytic cleavage of the transient metallacycle,^[15]^ whereas the fate of the residual ruthenium remained unknown. These critical aspects need to be addressed and solutions be found in order to render the reaction practical and relevant.

Complex **3 a** differs from the classical complexes commonly referred to as “Grubbs‐Hoveyda catalysts” in that the benzylidene unit carries an extra ethyl group rather than the usual H atom.[^16^, ^17^] As a result of the chelate structure, this substituent clashes into one of the mesityl groups of the NHC ligand resulting in severe congestion. As manifested in the X‐ray structure, this unfavorable disposition distorts the ligand sphere of **3 a** and may therefore account, at least in part, for the low yield with which the complex is formed.^[1]^ This handicap could be avoided by turning the ruthenacyclic ring by 90 °C such that it lies parallel rather than orthogonal to the mesityl group; such an arrangement requires the chloride ligands to be *cis*‐oriented. Although less widespread, ruthenium carbene complexes with such an overall coordination geometry are well known; they are commonly observed upon formal replacement of the ether oxygen of an archetype Grubbs‐Hoveyda catalyst by heteroatoms exerting a stronger *trans* influence.[^18^, ^19^, ^20^, ^21^, ^22^, ^23^] Provided that alkynes carrying appropriate such substituents are amenable to the light‐driven *gem* hydrogenation, the resulting ruthenium carbene complexes could adopt the desirable coordination geometry, which in turn might potentially improve the outcome of the reaction in terms of selectivity, efficiency and yield.

To test this opportunity, a representative set of substrates of type **2** was prepared and subjected to the standard hydrogenation conditions (Scheme 2). Despite the strong heteroatom donor sites with high affinity to ruthenium, which could outcompete binding of the triple bond to the metal center and hence prevent the reaction from occurring,^[24]^ all but the ester (**2 e**)[^25^, ^26^] and the bromo derivative **2 g** ^[22]^ reacted well. Specifically, irradiation of a solution of **1 b** (X=H) and readily available **2 b** (1.5 equiv.)^[27]^ in CH_2_Cl_2_ (0.1 M) under H_2_ atmosphere (balloon) at ambient temperature overnight resulted in a very clean and essentially quantitative formation of the expected *cis*‐configured complex **5 a**. The reaction was carried out using commercial photochemical equipment (EvoluChemTM LED, 365 nm, 18 W); it can be performed in ordinary glassware and works nicely on >1.2 gram scale. The structure of the resulting complex **5 a** in the solid state was confirmed by X‐ray diffraction (Figure 1);^[28]^ it proves that a net *gem* hydrogenation event has taken place.^[29]^ The complex itself shows the usual distorted square‐pyramidal coordination geometry about the Ru center carrying two *cis*‐disposed chloride substituents; as expected, a deleterious clash of the ethyl group adjacent to the carbene with the ancillary ligand is prevented.

**Scheme 2:**
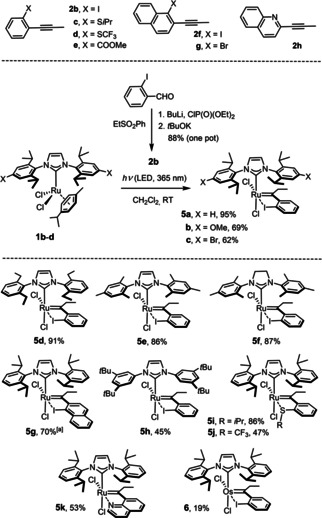
Formation of “second‐generation” ruthenium carbene complexes with *cis*‐disposed dichloride ligands by light‐driven *gem* hydrogenation. [a] A small amount (ca. 3 %) of the *trans*‐configured complex was also obtained in this case, see the Supporting Information.

**Figure 1:**
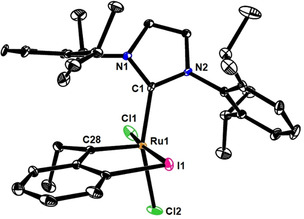
Structure of complex **5 a** in the solid state; H‐atoms and disorder of one of the 2,6‐diisopropylphenyl groups over two positions not shown for clarity; for the entire structure, see the Supporting Information.

A small collection of related *cis*‐configured complexes of type **5** was prepared analogously in good to excellent yields. The variations concern the heteroatom donor sites as well as the substitution pattern on the NHC ligand, which can feature a “saturated” or “unsaturated” backbone. Even ruthenium as the central atom can be replaced, although the formation of the corresponding osmium complex **6** was much less efficient for reasons that are not entirely clear at this point.^[30]^ Despite the poor productivity, this example is important from the conceptual viewpoint in that it shows for the first time that discrete carbene complexes involving metals other than ruthenium can be generated by *gem* hydrogenation.[^1^, ^2^, ^3^, ^4^, ^5^, ^6^, ^7^, ^8^] In view of the importance of metal carbenes in general as catalysts or reactive intermediates, this finding is deemed to provide an encouraging outlook.

The catalytic activity of the new iodo‐chelate complexes thus formed is akin to that of their analogues previously described in the literature;[^22^, ^31^] specifically, **5 a** and **5 e** both converted diallyl malonate **7** into product **8** in essentially quantitative yield within 90 minutes′ reaction time. This result suggests that the necessary *cis*→*trans* isomerization of the coordination sphere is not impeded on steric grounds by the additional ethyl substituent. Encouraged by this result, complex **5 a** was treated with stoichiometric amounts of (substituted) 2‐isopropoxystyrene (1–1.5 equiv.) in toluene at 80 °C in the hope of effecting carbene exchange by cross metathesis; gratifyingly, the reaction worked well on a gram scale (Scheme 3). The other tested iodo‐chelate complexes reacted analogously, even though the yields seem to be dependent on the size and substitution pattern of the NHC ligand, whereas complexes **5 i**–**k** containing sulfur or nitrogen in the chelate ring did not undergo this transformation. This favorable result shows that prototypical “second‐generation” Grubbs‐Hoveyda complexes of type **3** or the corresponding Grela variants (**3 c**)^[32]^ are now accessible in good to excellent overall yield in only three operations starting from commercial [(η^6^‐cymene)RuCl_2_]_2_ without the need to resort to phenyldiazomethane or other hazardous reagents. The new approach therefore constitutes an alternative to the use of propargyl alcohols (or chlorides), which enjoy widespread use as carbene sources.[^10^, ^11^, ^33^, ^34^] The fact that this new entry route does not require any phosphine that, in the end, has to be sacrificed, is an additional virtue.^[35]^ Unlocking the inherent *vic*‐dicarbene character of a triple bond through *gem* hydrogenation provides an attractive alternative that merits further investigation. Studies along these lines are being pursued in our laboratory.

**Scheme 3:**
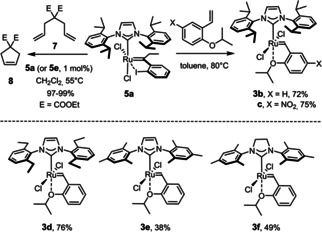
Test reaction and conversion of the *cis*‐configured ruthenium chelate complexes into prototype “second‐generation” Grubbs‐Hoveyda type catalysts by cross metathesis.

## Conflict of interest

The authors declare no conflict of interest.

## Supporting information

As a service to our authors and readers, this journal provides supporting information supplied by the authors. Such materials are peer reviewed and may be re‐organized for online delivery, but are not copy‐edited or typeset. Technical support issues arising from supporting information (other than missing files) should be addressed to the authors.

Supplementary
