# Supplementary material for: Light‐Driven gem Hydrogenation: An Orthogonal Entry into “Second‐Generation” Ruthenium Carbene Catalysts for Olefin Metathesis
Source: Chemistry. 2021 May 1;27(28):7663–6. doi: 10.1002/chem.202101176 (PMC8251631; doi:10.1002/chem.202101176)
Supplement: Supplementary file 1 — Supplementary [file CHEM-27-7663-s001.pdf]

Chemistry–A European Journal

Supporting Information

Light-Driven *gem* Hydrogenation: An Orthogonal Entry into "Second-Generation" Ruthenium Carbene Catalysts for Olefin Metathesis

Raphael J. Zachmann and Alois Fürstner*

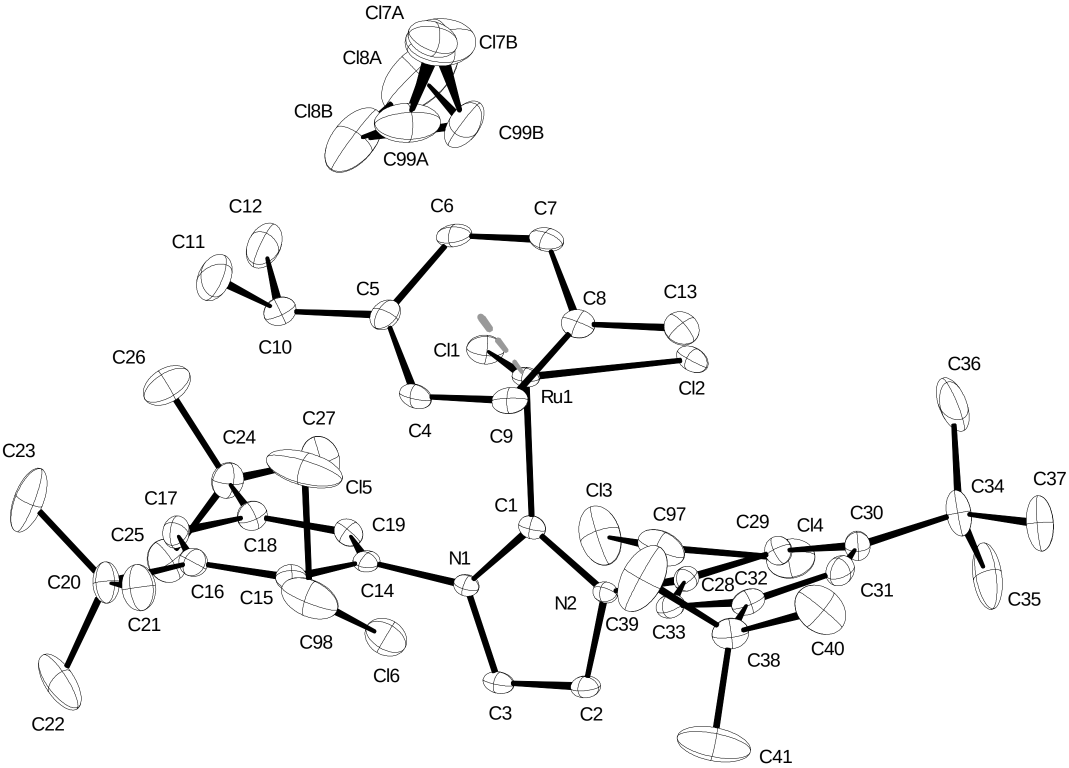

Figure S1. Structure of **1g**·3(CH_2Cl_2) in the solid state; atomic displacement ellipsoids shown at the 50% probability level, H atoms omitted for clarity.

X-ray Crystal Structure Analysis of Complex 1g·3(CH_2Cl_2): $C_{44} H_{64} Cl_8 N_2$ Ru, M_r = 1005.64 g · mol^{-1}, orange plate, crystal size 0.124 x 0.080 x 0.041 mm^3, monoclinic, space group *P2₁/n* [14], a = 11.1000(5) Å, b = 29.9661(15) Å, c = 15.1003(7) Å, β = 102.743(2)°, V = 4899.0(4) Å^3, T = 100(2) K, Z = 4, D_{calc} = 1.363 g · cm^3, λ = 0.71073 Å, μ(Mo-K_α) = 0.788 mm^{-1}, analytical absorption correction ($T_{\min}$ = 0.96, $T_{\max}$ = 0.99), Bruker-AXS Kappa Mach3 APEX-II diffractometer with Iμs microsource, 1.359 < θ < 27.499°, 133291 measured reflections, 11279 independent reflections, 9714 reflections with $I > 2\sigma(I)$, R_{int} = 0.0591. S = 1.196, 538 parameters, residual electron density +1.1 (0.90 Å from Cl3) / -1.3 (1.68 Å from C9) e · Å^{-3}.

The structure was solved by *SHELXT* and refined by full-matrix least-squares (*SHELXL*) against F^2 to R_1 = 0.049 [$I > 2\sigma(I)$], wR_2 = 0.103. **CCDC- 2065091.**

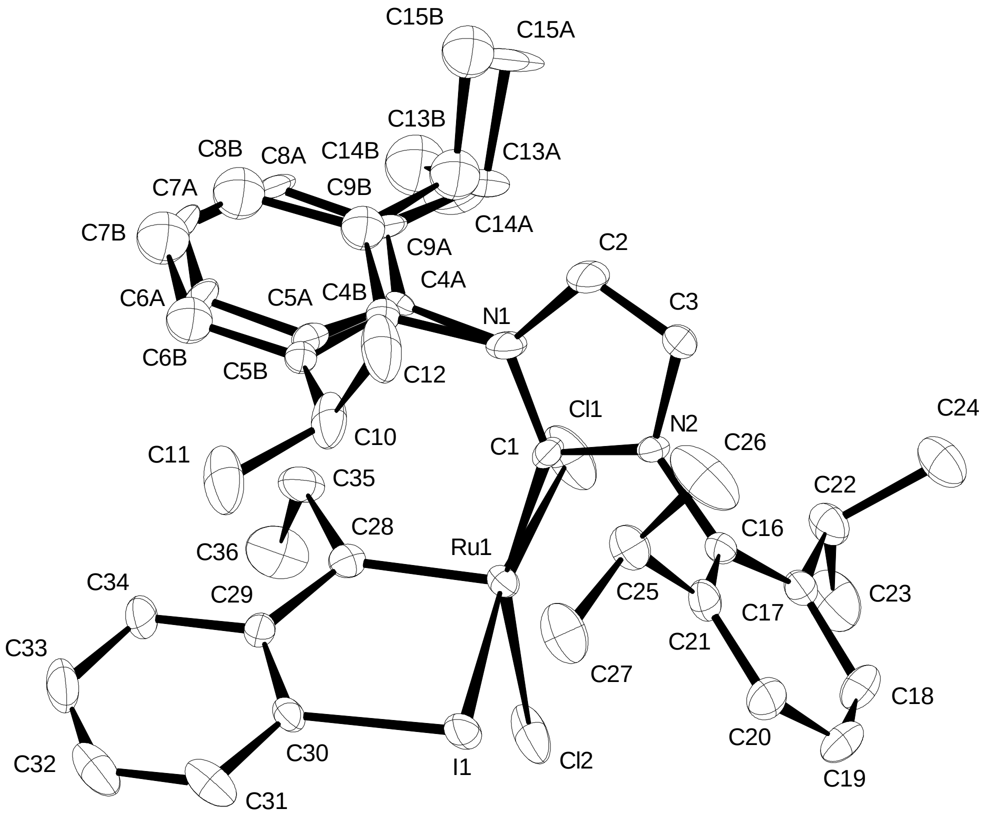

Figure S2. Structure of complex **5a** in the solid state; atomic displacement ellipsoids shown at the 50% probability level, H atoms omitted for clarity.

X-ray Crystal Structure Analysis of Complex 5a: $C_{36}H_{45}Cl_2IN_2Ru$, M_r = 804.61 g · mol^{-1}, orange brown needle, crystal size 0.115 x 0.022 x 0.020 mm^3, monoclinic, space group *P2₁/c* [14], a = 11.9234(6) Å, b = 22.9339(12) Å, c = 15.7265(9) Å, β = 99.992(2)°, V = 4235.2(4) Å^3, T = 100(2) K, Z = 4, D_{calc} = 1.262 g · cm^3, λ = 0.71073 Å, $\mu(Mo\text{-}K_\alpha)$ = 1.247 mm^{-1}, analytical absorption correction ($T_{\min}$ = 0.91, $T_{\max}$ = 1.00), Bruker-AXS Kappa Mach3 APEX-II diffractometer with Iµs microsource, 1.587 < θ < 31.633°, 143829 measured reflections, 14110 independent reflections, 11864 reflections with $I > 2\sigma(I)$, R_{int} = 0.0528. S = 1.171, 426 parameters, absolute structure parameter = -0.02(7), residual electron density +1.4 (3.01 Å from H34) / -1.7 (0.72 Å from I1) e · Å^{-3}.

A strongly disordered solvent mixture was present in solvent accessible voids; these solvents were omitted using SQUEZZE. The structure was solved by *SHELXT* and refined by full-matrix least-squares (*SHELXL*) against F^2 to R_1 = 0.060 [$I > 2\sigma(I)$], wR_2 = 0.129. **CCDC-2065092**.

GENERAL

Unless stated otherwise, all reactions were carried out under argon in flame-dried Schlenk glassware, ensuring rigorously inert conditions. The solvents were purified by distillation over the indicated drying agents and were stored and handled under argon: CH_2Cl_2 (CaH_2), pentane (Na/K alloy), THF (Na/K alloy), toluene (CaH_2). Benzene and *n*-hexane were degassed via freeze-pump-thaw cycles (3 x) and stored over molecular sieves 3Å. Flash chromatography: Merck Geduran silica gel 60 (40 – 63 μm).

NMR spectra were recorded on Bruker DPX 300, AMX 300, AV 400 or AV III 600 spectrometers in the solvents indicated; chemical shifts (δ) are given in ppm relative to TMS, coupling constants (*J*) in Hz. The solvent signals were used as references[1] and the chemical shifts converted to the TMS scale ($CDCl_3$: δ_C = 77.16 ppm; residual $CHCl_3$: δ_H = 7.26 ppm; CD_2Cl_2: δ_C = 54.00 ppm; residual $CHDCl_2$: δ_H = 5.32 ppm; C_6D_6: δ_C = 128.06 ppm; residual C_6HD_5: δ_H = 7.16 ppm).

IR: Alpha Platinum ATR spectrometer (Bruker), wavenumbers ($\tilde{\nu}$) in cm^{-1}.

MS (EI): Finnigan MAT 8200 (70 eV), DI-MS (EI): Finnigan MAT SSQ 7000, ESI-MS: ESQ 3000 (Bruker), Thermo Scientific LTQ-FT, or Thermo Scientific Exactive spectrometer. HRMS: Bruker APEX III FT-MS (7 T magnet), MAT 95 (Finnigan), Thermo Scientific LTQ-FT, Thermo Scientific Exactive instrument. GC-MS: Shimadzu GCMS-QP2010 Ultra instrument.

Elemental analysis was performed by MIKRO Lab (Mikroanalytisches Laboratorium Kolbe, Germany).

Hydrogen gas (N50, ≥99.999 Vol.%) was purchased from AirLiquide and was used without further purification. Deuterium gas (99.8 atom% D, 99.995% purity) was purchased from SigmaAldrich. H_2 and D_2 were handled with balloon techniques.

Unless stated otherwise, all commercially available compounds (abcr, Acros, TCI, Aldrich, Alfa Aesar) were used without further purification.

Light-sensitive reactions were carried out in glassware wrapped in aluminum foil with the fume hood light turned off.

Photolysis experiments were performed in the PhotoRedOxBox TC from HepatoChem (Figure S3) equipped with an EvoluChem™ LED (365 nm, 18 W). When the cooling function was used, water was used as the cooling agent (23°C).

Most reactions were carried out in quartz Schlenk tubes, but control experiments showed that standard borosilicate glassware is equally suitable and can be used without any noticeable effect on yield and purity.

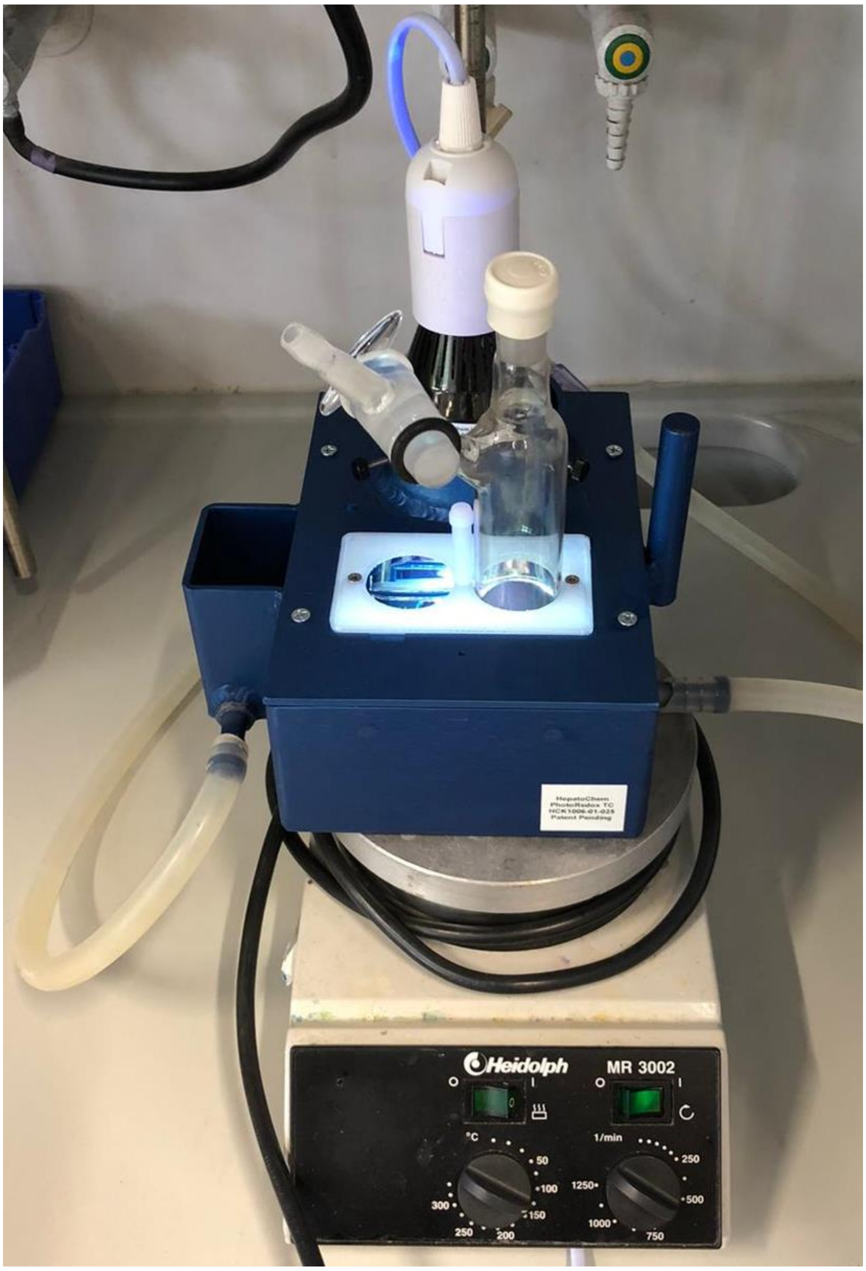

Figure S3. PhotoRedOxBox TC from Hepatochem.

STARTING MATERIALS

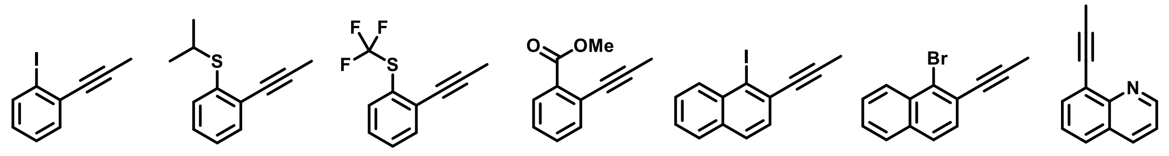
2b 2c 2d 2e 2f 2g 2h

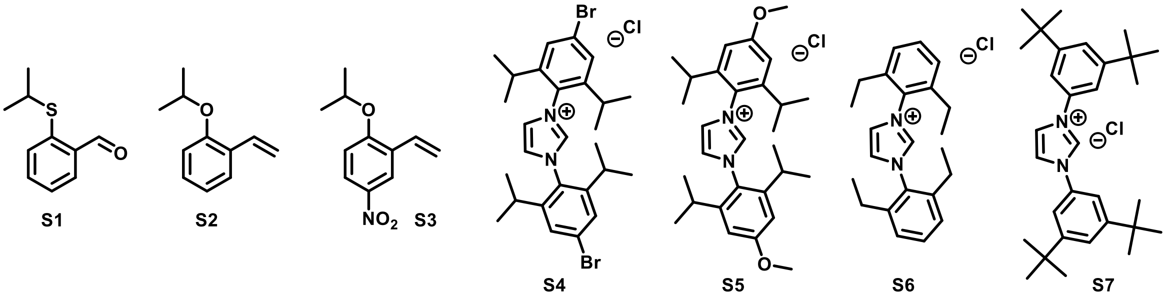
S1 S2 S3 S4 S5 S6 S7

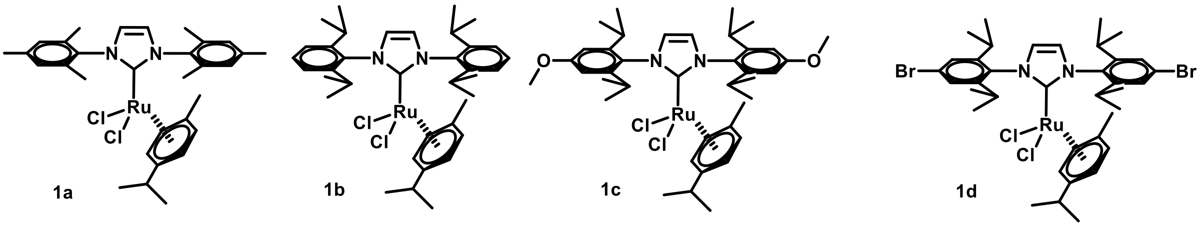
1a 1b 1c 1d

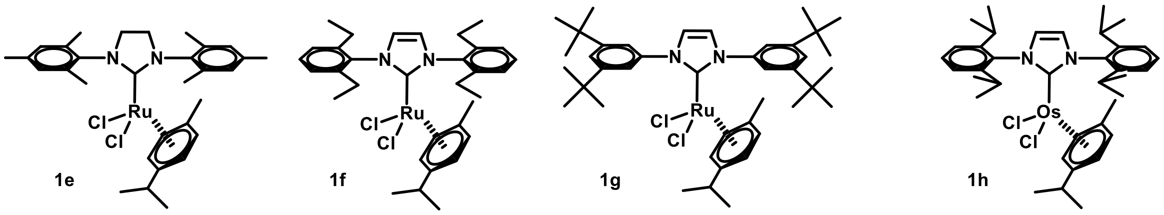
1e 1f 1g 1h

The compounds **2b**,[2] **2e**,[3] **2g**,[4] **S1**,[5] **S2**,[6] **S3**,[7] **S4**,[8] **S5**,[9] **S6**,[10] **S7**,[11] **1b**,[12] **1a**,[12] **1e**,[13] and **1h**[14] were prepared according to literature procedures.

ALKYNES

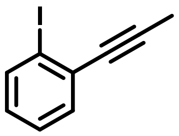

1-Iodo-2-(prop-1-yn-1-yl)benzene (2b). *n*-BuLi (1.6 M in hexanes, 3.23 mL, 5.17 mmol) was added dropwise to a solution of ethyl phenyl sulfone (880 mg, 5.17 mmol) in THF (70 mL) at –78°C. After stirring for 30 min, a solution of 2-iodobenzaldehyde (1.00 g, 4.31 mmol) in THF (10 mL) was introduced and stirring continued for 30 min before diethyl chlorophosphate (747 µL, 5.17 mmol) was added dropwise and the mixture was warmed to room temperature. After stirring for 3 h, the mixture was cooled to –78°C and KO^{t}Bu (2.42 g, 21.6 mmol) was added in one portion. After stirring for 15 min, the mixture was warmed to room temperature and stirring was continued overnight. Sat. aq. NH_4Cl (15 mL) was added, the organic layer was separated and the aqueous phase was extracted with EtOAc

(3 × 100 mL). The combined organic layers were dried over Na_2SO_4 and the solvent was removed under reduced pressure. The residue was purified by flash chromatography (silica, pentane) to provide the title compound as a colorless oil (914 mg, 88%). ^{1}H NMR (400 MHz, $CDCl_3$) δ 7.81 (dd, *J* = 8.0, 1.2 Hz, 1H), 7.40 (dd, *J* = 7.7, 1.7 Hz, 1H), 7.26 (td, *J* = 7.7, 1.2 Hz, 1H), 6.95 (td, *J* = 7.7, 1.7 Hz, 1H), 2.12 (s, 3H). ^{13}C NMR (101 MHz, $CDCl_3$) δ 138.7, 132.7, 130.6, 128.9, 127.8, 101.0, 90.4, 82.1, 4.7. The data are consistent with those reported in the literature.[2]

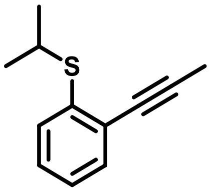

Isopropyl(2-(prop-1-yn-1-yl)phenyl)sulfane (2c). *n*-BuLi (1.6 M in hexanes, 9.81 mL, 15.7 mmol) was added dropwise to a solution of ethyl phenyl sulfone (2.67 g, 15.7 mmol) in THF (170 mL) at −78°C. After stirring for 30 min, a solution of aldehyde **S1** (2.36 g, 13.1 mmol) in THF (50 mL) was introduced and stirring continued for 30 min before diethyl chlorophosphate (2.27 mL, 15.7 mmol) was added dropwise and the mixture was warmed to room temperature. After stirring for 3 h, the mixture was cooled to −78°C and KO^{t}Bu (7.34 g, 65.4 mmol) was added in one portion. After stirring for 15 min, the mixture was warmed to room temperature and stirring was continued overnight. Sat. aq. NH_4Cl (50 mL) was added, the organic layer was separated and the aqueous phase was extracted with EtOAc (3 × 150 mL). The combined organic layers were dried over Na_2SO_4 and the solvent was removed under reduced pressure. The residue was purified by flash chromatography (silica, pentane/*tert*-butyl methyl ether, 50:1) to provide the title compound as a pale yellow oil (1.45 g, 58%). ^{1}H NMR (600 MHz, $CDCl_3$) δ 7.39 (dd, *J* = 7.7, 1.5 Hz, 1H), 7.30 (dd, *J* = 7.9, 1.1 Hz, 1H), 7.20 (td, *J* = 7.7, 1.5 Hz, 1H), 7.10 (td, *J* = 7.5, 1.3 Hz, 1H), 3.55 (hept, *J* = 6.7 Hz, 1H), 2.13 (s, 3H), 1.35 (d, *J* = 6.7 Hz, 6H). ^{13}C NMR (151 MHz, $CDCl_3$) δ 138.6, 133.0, 129.2, 127.9, 125.6, 125.1, 92.0, 78.1, 36.5, 23.1, 4.8. IR (film) ṽ 2962, 1462, 1234, 1154, 1072, 1036, 749, 456 cm^{-1}. HRMS (GC-EI) calcd. for $C_{12}H_{14}S$ [M$^+$]: 190.08107; found: 190.08136.

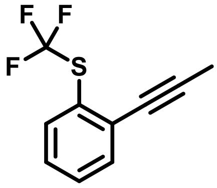

(2-(Prop-1-yn-1-yl)phenyl)(trifluoromethyl)sulfane (2d). *n*-BuLi (1.6 M in hexanes, 1.82 mL, 2.91 mmol) was added dropwise to a solution of ethyl phenyl sulfone (495 mg, 2.91 mmol) in THF (45 mL) at −78°C. After stirring for 30 min, a solution of 2-((trifluoromethyl)thio)benzaldehyde (500 mg, 2.43 mmol) in THF (7 mL) was added and stirring was continued for 30 min. Diethyl chlorophosphate (421 µL, 2.91 mmol) was added dropwise and the mixture was warmed to room temperature. After stirring for 3 h, the mixture was cooled to −78°C before KO^{t}Bu (1.36 g, 12.1 mmol) was added in one portion. After stirring for 15 min, the mixture was warmed to room temperature and stirring was continued overnight. Sat. aq. NH_4Cl (10 mL) was added, the organic layer was separated and the aqueous phase was extracted with EtOAc (3 × 50 mL). The combined organic layers were dried over Na_2SO_4 and the solvent was removed under reduced pressure. The residue was purified by flash chromatography (silica, pentane) to provide the title compound as

a colorless oil (395 mg, 75%). ^1H NMR (400 MHz, CD_2Cl_2) δ 7.68 (dd, *J* = 7.7, 1.1 Hz, 1H), 7.53 (dd, *J* = 7.8, 1.6 Hz, 1H), 7.42 (td, *J* = 7.6, 1.4 Hz, 1H), 7.34 (td, *J* = 7.7, 1.7 Hz, 1H), 2.11 (s, 3H). ^{13}C NMR (101 MHz, CD_2Cl_2) δ 137.0, 133.9, 131.2, 130.9, 130.4 (q, *J* = 308.5 Hz), 128.9, 126.7 (q, *J* = 1.8 Hz), 93.2, 77.8, 4.7. ^{19}F NMR (282 MHz, CD_2Cl_2) δ –42.3. IR (film) ṽ 2236, 1470, 1435, 1123, 1098, 1062, 1033, 974, 953, 756, 665, 542, 493, 467 cm^{-1}. HRMS (GC-EI) calcd. for $C_{10}H_7F_3S$ [M^+]: 216.02151; found: 216.02155.

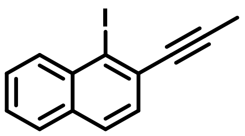

1-Iodo-2-(prop-1-yn-1-yl)naphthalene (2f). *n*-BuLi (1.6 M in hexanes, 1.24 mL, 1.98 mmol) was added dropwise to a solution of aryl bromide **2g** (456 mg, 1.75 mmol) in THF (15 mL) at –78°C. After stirring for 30 min, I_2 (548 mg, 2.16 mmol) was added in one portion. The mixture was warmed to room temperature and stirring continued for 2 h. Sat. aq. $Na_2S_2O_3$ (20 mL) was added, the organic layer was separated, and the aqueous phase was extracted with *tert*-butyl methyl ether (3 × 30 mL). The combined organic layers were dried over Na_2SO_4 and the solvent was removed under reduced pressure to provide the title compound as a yellow oil that was used without further purification (504 mg, 99%). ^1H NMR (400 MHz, $CDCl_3$) δ 8.18 (dd, *J* = 8.5, 1.0 Hz, 1H), 7.72 (dd, *J* = 8.2, 1.8 Hz, 2H), 7.56 (ddd, *J* = 8.4, 6.9, 1.4 Hz, 1H), 7.52 – 7.41 (m, 2H), 2.19 (s, 3H). ^{13}C NMR (101 MHz, $CDCl_3$) δ 135.0, 133.4, 132.8, 130.0, 129.1, 128.5, 128.4, 128.3, 126.9, 106.5, 91.4, 84.0, 4.9. IR (film) ṽ 2911, 2229, 1492, 1312, 1238, 1017, 944, 810, 742, 660, 519 cm^{-1}. HRMS (GC-EI) calcd. for $C_{13}H_9I$ [M^+]: 291.97435; found: 291.97447.

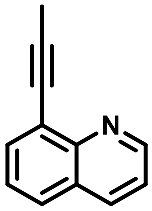

8-(Prop-1-yn-1-yl)quinolone (2h). *n*-BuLi (1.6 M in hexanes, 2.39 mL, 3.82 mmol) was added dropwise to a solution of ethyl phenyl sulfone (650 mg, 3.82 mmol) in THF (40 mL) at –78°C. After stirring for 30 min, a solution of quinoline-8-carbaldehyde (500 mg, 3.18 mmol) in THF (10 mL) was added. After stirring for another 30 min, diethyl chlorophosphate (552 µL, 3.82 mmol) was added dropwise and the mixture was warmed to room temperature. After stirring for 3 h, the mixture was cooled to –78°C and KO*t*Bu (1.78 g, 15.9 mmol) was added in one portion. The mixture was stirred for 15 min at this temperature and at room temperature overnight. Sat. aq. NH_4Cl (10 mL) was added, the organic layer was separated and the aqueous layer was extracted with EtOAc (3 × 50 mL). The combined organic phases were dried over Na_2SO_4 and the solvent was removed under reduced pressure. The residue was purified by flash chromatography (silica, pentane/*tert*-butyl methyl ether, 1:1 → 1:2) to provide the title compound as an orange oil (310 mg, 58%). ^1H NMR (400 MHz, $CDCl_3$) δ 9.04 (dd, *J* = 4.2, 1.8 Hz, 1H), 8.15 (dd, *J* = 8.3, 1.8 Hz, 1H), 7.87 (dd, *J* = 7.2, 1.4 Hz, 1H), 7.75 (dd, *J* = 8.2, 1.4 Hz, 1H), 7.50 – 7.39 (m, 2H), 2.26 (s, 3H). ^{13}C NMR (101 MHz, $CDCl_3$) δ 151.1, 148.4, 136.7, 134.2, 128.5, 127.8, 126.2, 124.2, 121.6, 92.5, 77.5, 5.4. IR (film) ṽ 3044, 1591, 1495,

1468, 1383, 1265, 828, 790, 730, 700, 661, 639 cm^{-1}. HRMS (GC-EI) calcd. for $C_{12}H_9N$ [M$^+$]: 167.07295; found: 167.07278.

RUTHENIUM η^6-CYMENE PRECATALYSTS

Representative Procedure. Preparation of the Ruthenium Complex 1d. KHMDS (568 mg, 2.85 mmol) was added in one portion to a suspension of imidazolium salt **S4** (1.28 g, 2.19 mmol) in Et_2O (35 mL) at room temperature. The mixture was vigorously stirred for 10 min before it was filtered through a pad of Celite and the filter cake was carefully rinsed with Et_2O (20 mL). The combined filtrates were evaporated in high vacuum to provide the corresponding N-heterocyclic carbene (NHC) as a pale yellow solid (1.05 g, 88%) which was directly used in the next step.

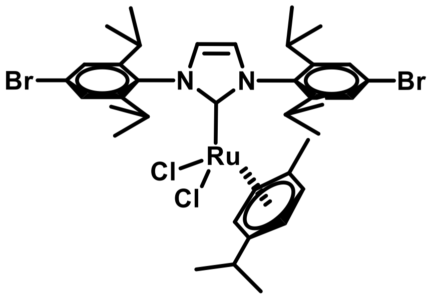

Under exclusion of light, a solution of this NHC (1.05 g, 1.92 mmol) in THF (25 mL) was added dropwise to a suspension of dichloro-(*p*-cymene)-ruthenium(II) dimer (479 mg, 782 μmol) in THF (25 mL) at room temperature. After stirring for 1 h in the dark, the solvent was removed in high vacuum. The red-orange residue was dissolved in Et_2O (5 mL) and the product precipitated by addition of pentane (50 mL) at room temperature. The supernatant was filtered off, and the precipitate was washed with pentane (2 × 20 mL) and dried in high vacuum to afford the title compound as an orange solid (1.46 g, 81%). ^{1}H NMR (400 MHz, C_6D_6) δ 7.44 (s, 4H), 6.37 (s, 2H), 4.80 (d, *J* = 5.8 Hz, 2H), 4.46 (d, *J* = 5.8 Hz, 2H), 3.20 (hept, *J* = 6.7 Hz, 4H), 2.51 (hept, *J* = 6.9 Hz, 1H), 1.67 (s, 3H), 1.28 (d, *J* = 6.6 Hz, 12H), 1.02 (d, *J* = 6.9 Hz, 6H), 0.85 (d, *J* = 6.8 Hz, 12H). ^{13}C NMR (101 MHz, C_6D_6) δ 174.7, 149.0, 139.1, 127.3, 126.5, 124.3, 105.3, 97.2, 86.4, 84.4, 30.5, 29.1, 25.9, 23.1, 22.8, 18.8. IR (film) ṽ 2964, 1462, 1443, 1387, 1366, 1327, 1228, 929, 807, 733, 699 cm^{-1}. HRMS (ESI$^+$) calcd. for $C_{37}H_{48}Br_2Cl_2N_2Ru$ [M–Cl]$^+$: 815.09108; found: 815.09004.

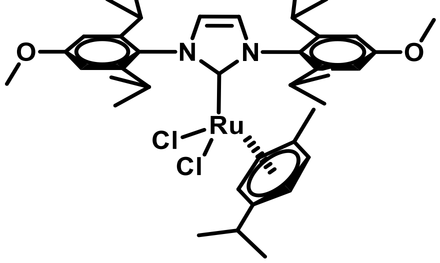

Complex 1c. Prepared analogously from imidazolium salt **S5** (904 mg, 1.86 mmol); sticky red solid (794 mg, 61%). ^{1}H NMR (400 MHz, C_6D_6) δ 6.87 (s, 4H), 6.56 (s, 2H), 4.94 (d, *J* = 5.8 Hz, 2H), 4.62 (d, *J* = 5.7 Hz, 2H), 3.45 – 3.32 (m, 10H), 2.68 (hept, *J* = 7.0 Hz, 1H), 1.81 (s, 3H), 1.49 (d, *J* = 6.6 Hz, 12H), 1.09 (d, *J* = 7.0 Hz, 6H), 1.06 (d, *J* = 6.8 Hz, 12H). ^{13}C NMR (101 MHz, C_6D_6) δ 174.9, 160.9, 147.9, 133.6, 128.6, 126.9, 109.1, 105.1, 96.9, 86.5, 84.4, 54.9, 30.5, 29.2, 26.3, 23.5, 22.9, 18.9. IR (film) ṽ 2962, 1597, 1466, 1441, 1336, 1306, 1242, 1217, 1199, 1033 cm^{-1}. HRMS (ESI$^+$) calcd. for $C_{39}H_{54}Cl_2N_2O_2Ru$ [M–Cl]$^+$: 719.29118; found: 719.19215.

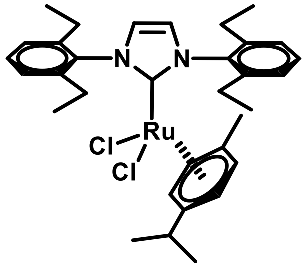

Complex 1f. Prepared analogously from imidazolium salt **S6** (850 mg, 2.30 mmol); the crude red-orange residue was dissolved in CH_2Cl_2 (5 mL) and the product precipitated by addition of pentane (80 mL) at 0°C. The supernatant was filtered off and the precipitate was washed with pentane (2 × 20 mL) and dried in high vacuum to give the title compound as an orange solid (1.05 g, 78%). ^1H NMR (400 MHz, C_6D_6) δ 7.23 – 7.18 (m, 2H), 7.03 (d, J = 7.7 Hz, 4H), 6.26 (s, 2H), 4.87 (d, J = 5.9 Hz, 2H), 4.36 (d, J = 5.9 Hz, 2H), 2.92 (dq, J = 15.3, 7.7 Hz, 4H), 2.59 (dq, J = 15.1, 7.5 Hz, 4H), 2.47 (hept, J = 7.0 Hz, 1H), 1.66 (s, 3H), 1.18 – 1.07 (m, 12H), 1.05 (d, J = 7.0 Hz, 6H). ^{13}C NMR (101 MHz, C_6D_6) δ 174.9, 142.1, 140.7, 129.4, 125.9, 125.4, 101.9, 96.5, 86.5, 86.3, 30.7, 25.5, 22.9, 18.1, 14.7. IR (film) ṽ 2963, 2931, 2873, 1459, 1390, 1269, 935, 803, 760, 728, 696 cm^{-1}. HRMS (ESI$^+$) calcd. for $C_{33}H_{42}Cl_2N_2Ru$ $[M{-}Cl]^+$: 603.20745; found: 603.20786.

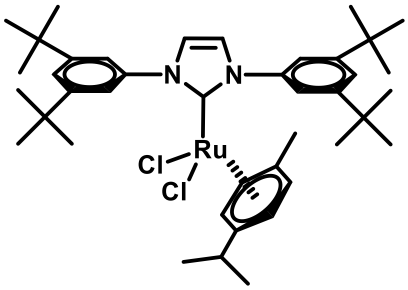

Complex 1g. Prepared analogously from imidazolium salt **S7** (592 mg, 1.23 mmol); the crude red-orange material was suspended in benzene (10 mL), the mixture was filtered through a pad of Celite and the filter cake was washed with benzene (30 mL). The combined filtrates were concentrated to 1/5 of its original volume. Addition of pentane (150 mL) at 0°C provided an orange solid, which was washed with pentane (2 × 20 mL) and dried in high vacuum. The material was dissolved in CH_2Cl_2 (10 mL), the solution was loaded on a flame-dried silica pad under Ar and the product quickly eluted with CH_2Cl_2/MeOH (50:1, 50 mL). An orange band was collected while a yellow residue stayed on top of the silica pad. Concentration of the product-containing fractions in high vacuum provided the title compound as an orange solid (220 mg, 31%). Crystals suitable for X-ray analysis were obtained by slowly cooling a concentrated solution in CH_2Cl_2 to −50°C over the course of 48 h. ^1H NMR (400 MHz, C_6D_6) δ 7.71 (d, J = 1.7 Hz, 4H), 7.59 (t, J = 1.7 Hz, 2H), 6.43 (s, 2H), 5.04 (d, J = 5.9 Hz, 2H), 4.34 (d, J = 5.6 Hz, 2H), 2.72 (hept, J = 6.9 Hz, 1H), 1.78 (s, 3H), 1.40 (s, 36H), 1.10 (d, J = 7.0 Hz, 6H). ^{13}C NMR (101 MHz, C_6D_6) δ 176.3, 150.9, 142.1, 125.2, 123.9, 122.4, 104.9, 99.4, 88.3, 82.3, 35.3, 31.7, 31.0, 22.9, 18.9. IR (film) ṽ 2957, 1592, 1434, 1363, 1302, 1247, 728, 709, 691 cm^{-1}. HRMS (ESI$^+$) calcd. for $C_{41}H_{58}Cl_2N_2Ru$ $[M{-}Cl]^+$: 715.33265; found: 715.33409.

cis-DICHLORO RUTHENIUM CARBENE COMPLEXES

Representative Procedure. Preparation of the Ruthenium Carbene Complex 5a. Alkyne **2b** (602 mg, 2.41 mmol) was added to a solution of the ruthenium cymene complex **1b** (1.18 g, 1.61 mmol) in CH_2Cl_2 (16 mL) in a flame dried quartz Schlenk tube under argon. The tube was

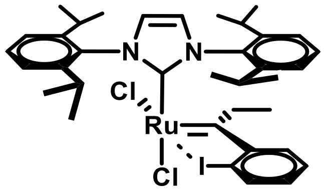

closed with a rubber septum and a balloon filled with hydrogen was connected. Under vigorous stirring, the gas atmosphere was exchanged via an outlet cannula and the light source was switched on. After 2 min, the outlet cannula was removed (balloon still attached!) and the mixture was stirred overnight at room temperature under hydrogen atmosphere with constant irradiation at λ = 365 nm. The mixture was directly loaded on a flame-dried silica pad under argon, eluting with CH_2Cl_2/Et_2O (1:1, 100 mL). A brown-yellow band was collected, whereas a dark residue stayed on top of the silica pad. The product-containing fractions were concentrated and the dark brown residue was triturated with pentane (30 mL). The supernatant was filtered off and the remaining solid was washed with pentane (2 × 20 mL). Drying in high vacuum afforded the title compound as a beige-brown solid (1.25 g, 95%). The same result was obtained when the reaction was carried out in standard borosilicate glassware.

Crystals suitable for X-ray analysis were obtained by layering a concentrated CH_2Cl_2 solution with pentane at –20°C followed by slow diffusion of the pentane into the CH_2Cl_2 layer over the course of several days. ^{1}H NMR (400 MHz, CD_2Cl_2) δ 7.65 – 7.55 (m, 2H), 7.51 (ddd, *J* = 12.6, 7.9, 1.7 Hz, 2H), 7.46 – 7.28 (m, 6H), 7.22 (d, *J* = 2.0 Hz, 1H), 6.76 (dd, *J* = 5.6, 3.6 Hz, 1H), 4.47 (dq, *J* = 13.3, 7.5 Hz, 1H), 4.27 (hept, *J* = 6.7 Hz, 1H), 3.55 (hept, *J* = 6.7 Hz, 1H), 2.31 (hept, *J* = 6.7 Hz, 1H), 1.82 (dq, *J* = 13.3, 7.4 Hz, 1H), 1.72 (hept, *J* = 6.9, 6.4 Hz, 1H), 1.54 (d, *J* = 6.5 Hz, 3H), 1.50 (d, *J* = 6.6 Hz, 3H), 1.47 (d, *J* = 6.8 Hz, 3H), 1.22 (d, *J* = 7.0 Hz, 3H), 1.20 (d, *J* = 6.9 Hz, 3H), 1.12 (d, *J* = 6.7 Hz, 3H), 0.85 (t, *J* = 7.5 Hz, 3H), 0.60 (d, *J* = 6.8 Hz, 3H), -0.05 (d, *J* = 6.7 Hz, 3H). ^{13}C NMR (101 MHz, CD_2Cl_2) δ 309.2, 183.5, 157.2, 149.6, 147.8, 146.0, 145.1, 136.8, 135.8, 135.4, 132.0, 130.6, 130.5, 129.4, 127.9, 126.4, 126.1, 125.9, 124.6, 123.4, 122.6, 98.3, 50.5, 29.6, 29.5, 29.4, 28.9, 28.2, 27.8, 27.2, 25.8, 23.7, 22.7, 22.3, 19.6, 9.1. IR (film) ṽ 2963, 2928, 1462, 1441, 1396, 1387, 1321, 1307, 1263, 802, 758, 732, 702 cm^{-1}. HRMS (APPI$^+$) calcd. for $C_{36}H_{45}Cl_2IN_2Ru$ [M$^+$]: 804.10437; found: 804.10437. Anal. calcd. for $C_{36}H_{45}Cl_2IN_2Ru$: C, 53.74; H, 5.64; N, 3.48; found: C, 53.37; H, 5.55; N, 3.39.

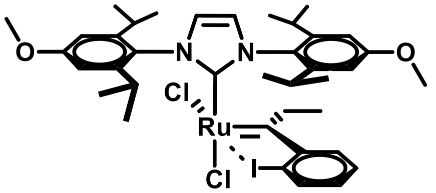

Ruthenium Complex 5b. Prepared analogously from the ruthenium cymene complex **1c** (204 mg, 267 µmol) and alkyne **2b** (101 mg, 406 µmol). The crude material was purified by flash chromatography (silica, pentane/*tert*-butyl methyl ether, 1:1 → 1:2) to provide the title complex as a brown solid (153 mg, 69%). ^{1}H NMR (400 MHz, CD_2Cl_2) δ 7.62 (dd, *J* = 7.3, 1.6 Hz, 1H), 7.54 (dd, *J* = 7.7, 1.9 Hz, 1H), 7.42 – 7.30 (m, 3H), 7.15 (d, *J* = 2.0 Hz, 1H), 6.97 (d, *J* = 2.9 Hz, 1H), 6.89 (d, *J* = 2.8 Hz, 1H), 6.79 (d, *J* = 2.9 Hz, 1H), 6.25 (d, *J* = 2.9 Hz, 1H), 4.57 (dq, *J* = 14.9, 7.5 Hz, 1H), 4.20 (hept, *J* = 6.5 Hz, 1H), 3.91 (s, 3H), 3.86 (s, 3H), 3.50 (hept, *J* = 6.7 Hz, 1H), 2.28 (hept, *J* = 6.8 Hz, 1H), 1.98 (dq, *J* = 14.6, 7.4 Hz, 1H), 1.68 (hept, *J* = 7.3 Hz, 1H), 1.52 (d, *J* = 6.6 Hz, 3H), 1.47 (d, *J* = 6.6 Hz, 3H), 1.44 (d, *J* = 6.9 Hz, 3H), 1.20 (dd, *J* =

6.8, 2.3 Hz, 6H), 1.10 (d, *J* = 6.7 Hz, 3H), 0.89 (t, *J* = 7.4 Hz, 3H), 0.59 (d, *J* = 6.8 Hz, 3H), -0.05 (d, *J* = 6.7 Hz, 3H). ^{13}C NMR (101 MHz, CD_2Cl_2) δ 308.8, 184.2, 162.1, 161.1, 157.3, 151.3, 149.4, 147.6, 146.7, 135.9, 130.4, 130.1, 129.3, 128.3, 128.1, 126.7, 122.5, 110.8, 110.0, 109.9, 109.4, 98.3, 56.0, 50.8, 29.9, 29.6, 29.1, 28.0, 27.6, 27.0, 25.7, 23.6, 22.8, 22.5, 19.6, 9.2. IR (film) ṽ 2962, 1598, 1465, 1439, 1333, 1305, 1253, 1214, 1195, 1034 cm^{-1}. HRMS (ESI$^+$) calcd. for $C_{38}H_{49}Cl_2IN_2O_2Ru$ [M–Cl]$^+$: 829.15653; found: 829.15780.

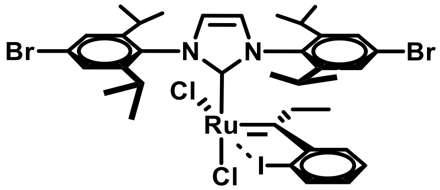

Ruthenium Complex 5c. Prepared analogously from ruthenium cymene complex **1d** (378 mg, 417 μmol) and alkyne **2b** (166 mg, 665 μmol). The crude material was purified by flash chromatography (silica, pentane/*tert*-butyl methyl ether, 1:1) to provide the title compound as a brown solid (260 mg, 62%). Solutions of this complex under air tend to decompose within several hours. ^{1}H NMR (400 MHz, CD_2Cl_2) δ 7.65 – 7.60 (m, 2H), 7.54 (s, 1H), 7.54 – 7.50 (m, 1H), 7.44 (s, 1H), 7.43 – 7.40 (m, 2H), 7.35 (s, 1H), 7.18 (s, 1H), 6.85 (s, 1H), 4.62 (dq, *J* = 13.2, 7.5 Hz, 1H), 4.08 (hept, *J* = 5.9, 5.4 Hz, 1H), 3.49 (hept, *J* = 6.6 Hz, 1H), 2.34 (hept, *J* = 6.3 Hz, 1H), 2.16 – 1.96 (m, 1H), 1.72 (hept, *J* = 6.7, 6.0 Hz, 1H), 1.53 – 1.47 (m, 3H), 1.50 – 1.41 (m, 6H), 1.22 – 1.16 (m, 6H), 1.13 – 1.08 (m, 3H), 0.89 (t, *J* = 7.5 Hz, 3H), 0.62 (d, *J* = 6.8 Hz, 3H), 0.00 (d, *J* = 6.6 Hz, 3H). ^{13}C NMR (101 MHz, CD_2Cl_2) δ 309.1, 184.3, 157.2, 152.0, 150.3, 148.4, 147.6, 135.9, 135.8, 134.4, 130.9, 129.6, 129.5, 129.0, 128.1, 127.9, 127.0, 126.7, 126.5, 124.9, 122.6, 97.7, 51.2, 30.0, 29.8, 29.7, 29.2, 27.8, 27.5, 26.9, 25.7, 23.4, 22.8, 22.4, 19.7, 9.2. IR (film) ṽ 2964, 2930, 1571, 1463, 1441, 1388, 1322, 937, 865, 806, 761 cm^{-1}. HRMS (ESI$^+$) calcd. for $C_{36}H_{43}Br_2Cl_2IN_2Ru$ [M–Cl]$^+$: 924.95642; found: 924.95479.

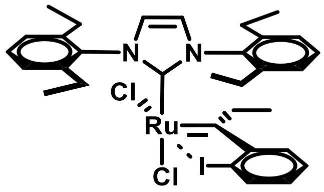

Ruthenium Complex 5d. Prepared analogously from the ruthenium cymene complex **1f** (211 mg, 331 μmol) and alkyne **2b** (124 mg, 497 μmol). The reaction mixture was loaded on a flame-dried silica pad under argon, eluting with CH_2Cl_2/Et_2O (1:1, 50 mL). A brown-yellow band was collected and a dark residue stayed on top of the silica pad. The product-containing fractions were concentrated and the green residue was triturated with pentane (30 mL). The supernatant was filtered off, the remaining solid was dissolved in toluene (5 mL) and the product precipitated with pentane (40 mL) at room temperature. The solid material was filtered off and dried in high vacuum to afford the title compound as a green solid (225 mg, 91%). ^{1}H NMR (400 MHz, CD_2Cl_2) δ 7.56 (dd, *J* = 13.3, 7.6 Hz, 2H), 7.46 (dd, *J* = 7.8, 1.4 Hz, 1H), 7.44 – 7.35 (m, 1H), 7.39 – 7.29 (m, 4H), 7.29 (ddd, *J* = 7.6, 3.9, 1.4 Hz, 2H), 7.06 (d, *J* = 2.0 Hz, 1H), 6.60 (dd, *J* = 7.6, 1.6 Hz, 1H), 4.70 (dq, *J* = 13.5, 7.5 Hz, 1H), 3.11 (ddq, *J* = 30.2, 15.1, 7.6 Hz, 2H), 2.95 (dq, *J* = 15.3, 7.7 Hz, 1H), 2.63 (ddq, *J* = 24.3, 15.1, 7.5 Hz, 2H), 2.32 (dq, *J* = 14.8, 7.4 Hz, 1H), 2.03 (dq, *J* = 13.4, 7.6 Hz, 1H), 1.43 – 1.28 (m, 1H), 1.31 – 1.14 (m, 10H), 0.83 (t, *J* = 7.5 Hz, 3H), 0.56 (t, *J* = 7.5 Hz, 3H). ^{13}C NMR (101 MHz, CD_2Cl_2) δ 308.8, 180.7, 157.0, 144.8, 143.2, 141.9, 141.4, 137.4, 136.1,

135.1, 131.6, 130.4, 130.3, 129.1, 128.8, 127.5, 127.4, 127.0, 126.6, 126.0, 122.8, 96.9, 50.6, 27.0, 26.7, 25.2, 24.5, 16.0, 15.0, 14.6, 9.1. IR (film) ṽ 2965, 2933, 2873, 1460, 1431, 1400, 1317, 1272, 810, 761 cm^{-1}. HRMS (ESI+) calcd. for $C_{32}H_{37}Cl_2IN_2Ru$ $[M–Cl]^+$: 713.07280; found: 713.07298.

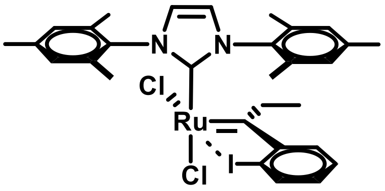

Ruthenium Complex 5e. Prepared analogously from Ru-cymene complex **1a** (290 mg, 475 μmol) and alkyne **2b** (178 mg, 712 μmol). During the purification, all manipulations were carried out in air with reagent-grade solvents. The mixture was directly loaded on a silica pad, eluting with CH_2Cl_2/Et_2O (1:1, 50 mL). A green band was collected while a dark residue stayed on top of the silica pad. The product-containing fractions were concentrated to provide a dark green residue which was re-dissolved in CH_2Cl_2 (5 mL) and the product was precipitated with pentane (50 mL) at room temperature. The supernatant was filtered off and the remaining solid was washed with pentane (2 × 20 mL). Drying in high vacuum afforded the title compound as a green solid (295 mg, 86%). ^1H NMR (400 MHz, CD_2Cl_2) δ 7.60 – 7.55 (m, 1H), 7.45 – 7.39 (m, 1H), 7.38 – 7.30 (m, 2H), 7.26 (d, *J* = 2.0 Hz, 1H), 7.19 (s, 1H), 7.04 (s, 1H), 7.00 (s, 1H), 6.96 (d, *J* = 2.0 Hz, 1H), 6.32 (s, 1H), 4.88 (dq, *J* = 13.8, 7.5 Hz, 1H), 2.62 (s, 3H), 2.40 (s, 3H), 2.33 (s, 3H), 2.32 (s, 3H), 2.28 – 2.20 (m, 1H), 2.19 (s, 3H), 0.95 (s, 3H), 0.89 (t, *J* = 7.5 Hz, 3H). ^{13}C NMR (101 MHz, CD_2Cl_2) δ 308.7, 179.4, 157.3, 141.1, 139.6, 138.5, 137.4, 136.2, 136.1, 135.3, 135.1, 134.9, 131.1, 130.5, 130.1, 130.1, 129.6, 128.7, 126.3, 126.0, 122.4, 96.6, 50.9, 21.5, 21.4, 20.6, 19.3, 17.3, 9.1. IR (film) ṽ 2959, 2919, 1483, 1432, 1398, 1380, 1317, 1265, 1011, 853, 762, 732, 700 cm^{-1}. HRMS (ESI+) calcd. for $C_{30}H_{33}Cl_2IN_2Ru$ $[M–Cl]^+$: 685.04150; found: 685.04187.

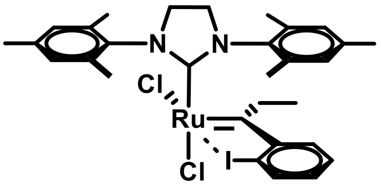

Ruthenium Complex 5f. Prepared analogously from the ruthenium cymene complex **1e** (101 mg, 165 μmol) and alkyne **2b** (61.6 mg, 247 μmol). The crude material residue was triturated with pentane and the supernatant was filtered off. The residual green solid was washed with pentane (2 × 20 mL) before it was re-dissolved in CH_2Cl_2 (5 mL) and the product was precipitated with pentane (30 mL) at room temperature. Washing with pentane (10 mL) and drying in high vacuum afforded the title compound as a pale green solid (103 mg, 87%). ^1H NMR (400 MHz, CD_2Cl_2) δ 7.60 (dd, *J* = 7.8, 1.3 Hz, 1H), 7.40 (ddd, *J* = 7.4, 1.5 Hz, 1H), 7.31 (ddd, *J* = 7.8, 6.8, 1.0 Hz, 1H), 7.22 (dd, *J* = 8.1, 1.5 Hz, 1H), 7.11 (d, *J* = 2.1 Hz, 1H), 6.99 (d, *J* = 1.8 Hz, 1H), 6.93 (d, *J* = 1.7 Hz, 1H), 6.22 (d, *J* = 2.0 Hz, 1H), 4.80 (dq, *J* = 14.0, 7.5 Hz, 1H), 4.23 (ddd, *J* = 11.4, 10.1 Hz, 1H), 4.11 – 3.88 (m, 2H), 3.82 (ddd, *J* = 11.5, 10.1 Hz, 1H), 2.76 (s, 3H), 2.49 (s, 3H), 2.47 (s, 3H), 2.35 (s, 3H), 2.27 (s, 3H), 2.10 (dq, *J* = 13.5, 7.5 Hz, 1H), 1.23 (s, 3H), 0.84 (t, *J* = 7.5 Hz, 3H). ^{13}C NMR (101 MHz, CD_2Cl_2) δ 308.3, 214.2, 157.2, 140.4, 140.0, 138.7, 138.4, 136.5, 136.5, 135.8, 134.9, 134.8, 131.2, 130.6, 130.3, 130.3, 130.0, 128.6, 122.7, 97.2, 52.5, 52.2,

50.3, 21.4, 21.3, 20.8, 20.8, 19.3, 17.6, 9.4. IR (film) ṽ 2958, 2918, 1482, 1431, 1401, 1264, 1012, 852, 762, 732 cm^{-1}. HRMS (ESI+) calcd. for $C_{30}H_{35}Cl_2IN_2Ru$ [M–Cl]+: 687.05715; found: 687.05685.

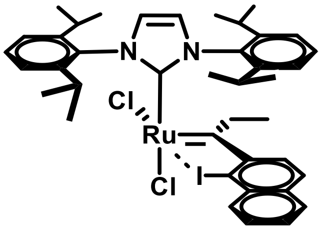

Ruthenium Complex *cis*-5g. Prepared analogously from the ruthenium cymene complex **1b** (91 mg, 121 µmol) and alkyne **2f** (45.3 mg, 182 µmol). The crude material was purified by flash chromatography (silica, pentane/*tert*-butyl methyl ether, 2:1 → 1:2). A minor, pre-eluting yellow fraction and a major green fraction were collected. Concentration of the main fraction provided the title compound as a green-brown solid (132 mg, 70%). ^{1}H NMR (400 MHz, CD_2Cl_2) δ 7.94 – 7.88 (m, 1H), 7.82 – 7.77 (m, 2H), 7.69 (ddd, *J* = 8.1, 6.8, 1.1 Hz, 1H), 7.64 (t, *J* = 7.8 Hz, 1H), 7.59 – 7.53 (m, 2H), 7.48 – 7.39 (m, 5H), 7.21 (d, *J* = 2.0 Hz, 1H), 6.71 (t, *J* = 4.6 Hz, 1H), 4.73 (dq, *J* = 13.5, 7.5 Hz, 1H), 4.27 (hept, *J* = 6.4 Hz, 1H), 3.60 (hept, *J* = 6.1 Hz, 1H), 2.32 (hept, *J* = 6.5, 6.0 Hz, 1H), 1.91 (dq, *J* = 14.8, 7.4 Hz, 1H), 1.67 – 1.59 (m, 1H), 1.57 (d, *J* = 6.6 Hz, 3H), 1.55 – 1.50 (m, 6H), 1.22 (dd, *J* = 6.9, 3.7 Hz, 6H), 1.12 (d, *J* = 6.7 Hz, 3H), 0.90 (t, *J* = 7.4 Hz, 3H), 0.51 (d, *J* = 6.8 Hz, 3H), -0.44 (d, *J* = 6.7 Hz, 3H). ^{13}C NMR (101 MHz, CD_2Cl_2) δ 308.6, 183.3, 155.8, 149.6, 147.9, 146.1, 145.1, 136.9, 135.6, 135.0, 133.3, 132.1, 131.3, 130.6, 130.5, 129.9, 129.1, 128.5, 128.0, 126.3, 126.2, 125.9, 124.7, 123.5, 121.2, 104.7, 51.4, 29.7, 29.4, 29.4, 28.9, 28.2, 27.8, 27.2, 25.8, 23.7, 22.8, 22.4, 19.4, 8.7. IR (film) ṽ 2963, 2928, 1462, 1396, 1322, 1260, 802, 756, 733, 703 cm^{-1}. HRMS (APPI+) calcd. for $C_{40}H_{47}Cl_2IN_2Ru$ [M+]: 854.11990; found: 854.11927.

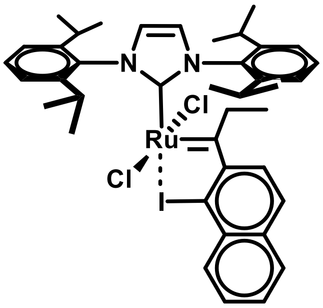

Concentration of the yellow fraction provided the *trans*-configured dichloro carbene complex *trans*-**5g** as a brown oil (5 mg, 3%), which analyzed as follows: ^{1}H NMR (400 MHz, CD_2Cl_2) δ 7.97 (dd, *J* = 8.4, 1.0 Hz, 1H), 7.82 – 7.76 (m, 2H), 7.71 (ddd, *J* = 8.1, 6.8, 1.1 Hz, 1H), 7.64 (t, *J* = 7.7 Hz, 1H), 7.56 (tdd, *J* = 7.7, 6.5, 1.4 Hz, 2H), 7.50 (d, *J* = 8.9 Hz, 1H), 7.45 (q, *J* = 2.7, 2.2 Hz, 2H), 7.41 (dd, *J* = 7.7, 1.5 Hz, 1H), 7.37 (d, *J* = 1.9 Hz, 1H), 7.22 (d, *J* = 1.9 Hz, 1H), 6.70 (dd, *J* = 6.0, 3.2 Hz, 1H), 4.46 (dq, *J* = 13.6, 7.5 Hz, 1H), 4.13 (hept, *J* = 6.8 Hz, 1H), 3.61 (hept, *J* = 6.5 Hz, 1H), 2.35 (hept, *J* = 13.6, 6.9 Hz, 1H), 1.67 (hept, *J* = 6.6 Hz, 1H), 1.60 – 1.57 (m, 4H), 1.55 (dd, *J* = 6.6 Hz, 6H), 1.23 (d, *J* = 7.0 Hz, 3H), 1.18 (d, *J* = 6.9 Hz, 3H), 1.11 (d, *J* = 6.7 Hz, 3H), 1.08 (t, *J* = 7.4 Hz, 3H), 0.52 (d, *J* = 6.8 Hz, 3H), –0.42 (d, *J* = 6.7 Hz, 3H).

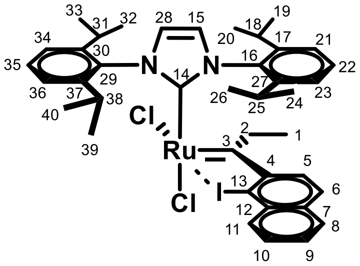

Atom	δ (ppm)	COSY	HSQC	HMBC	NOESY
1 C	8.51		1	2', 2''	
H3	0.89	2', 2''	1	2, 3	5
2 C	51.29		2', 2''	1	
H'	4.73	1, 2''	2	1, 3, 4	2'', 27
H''	1.91	1, 2'	2	1, 3, 4	2', 5
3 C	308.54			1, 2', 2'', 5	
4 C	155.69			2', 2'', 5, 6	
5 C	121.01		5		
H	7.47	6	5	3, 4, 7, 13	1, 2''
6 C	130.36		6	8	
H	7.81	5	6	4, 8, 12	
7 C	133.15			5, 9, 11	
8 C	128.91		8	6, 10, 11	
H	7.79	9	8	6, 10, 12	
9 C	128.32		9		
H	7.69	8, 10	9	7, 11	
10 C	129.71		10	8	
H	7.56	9, 11	10	8, 12	
11 C	131.14		11	9	
H	7.92	10	11	7, 8, 13	
12 C	134.90			6, 8, 10	
13 C	104.55			5, 11	
14 C	183.13			15, 28	

Atom	δ (ppm)	COSY	HSQC	HMBC	NOESY
15 C	126.20		15	28	
H	7.40	28	15	14, 28	19, 26
16 C	135.44			18, 21, 23, 25	
17 C	149.50			18, 19, 20, 22	
18 C	29.53		18	19, 20, 21	
H	4.27	19, 20	18	16, 17, 19, 20, 21	19, 20
19 C	27.66		19	18, 20	
H3	1.21	18	19	17, 18, 20	15, 18, 21
20 C	22.27		20	18, 19	
H3	1.56	18	20	17, 18, 19	18, 21
21 C	126.04		21	18, 23	
H	7.56	22	21	16, 18, 23	19, 20
22 C	131.94		22		
H	7.64	21, 23	22	17, 24	
23 C	124.54		23	21, 25	
H	7.40	22	23	16, 21, 25	26, 27
24 C	145.94			22, 25, 26, 27	
25 C	29.28		25	23, 26, 27	
H	2.31	26, 27	25	16, 23, 24, 26, 27	26, 27, 33, 39
26 C	28.02		26	25, 27	
H3	1.11	25	26	24, 25, 27	15, 23, 25, 39
27 C	22.64		27	25, 26	
H3	1.52	25	27	24, 25, 26	2', 23, 25

Atom	δ (ppm)	COSY	HSQC	HMBC	NOESY
28 C	127.83		28	15	
H	7.21	15	28	14, 15	32, 39
29 C	136.72			31, 34, 36, 38	
30 C	147.75			31, 32, 33, 35	
31 C	28.77		31	32, 33, 34	
H	3.60	32, 33	31	29, 30, 32, 33, 34	32, 33
32 C	27.03		32	31, 33	
H3	1.22	31	32	30, 31, 33	28, 31, 34
33 C	23.57		33	31, 32	
H3	1.52	31	33	30, 31, 32	25, 31, 34
34 C	125.75		34	31, 36	
H	7.45		34	29, 31, 36	32, 33
35 C	130.43		35		
H	7.45	36	35	30, 37	
36 C	123.38		36	34, 38	
H	6.71	35	36	29, 34, 38	40
37 C	144.92			35, 38, 39, 40	
38 C	29.24		38	36, 39, 40	
H	1.62	39, 40	38	29, 36, 37, 39, 40	39, 40
39 C	25.65		39	38, 40	
H3	0.52	38	39	37, 38, 40	25, 26, 28, 38, 40
40 C	19.24		40	38, 39	
H3	-0.44	38	40	37, 38, 39	36, 38, 39

Table S1 NMR analysis of the *cis*-configured Ru carbene *cis*-**5g**; this dataset was recorded on a 600 MHz spectrometer in CD_2Cl_2.

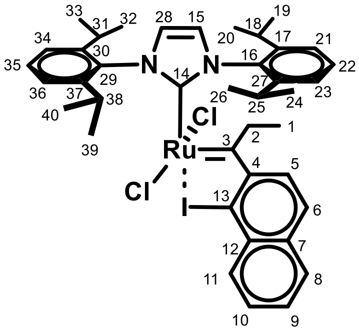

Atom	δ (ppm)	J	COSY	HSQC	HMBC
1 C	11.30			1	2', 2''
H3	1.07		2', 2''	1	2, 3
2 C	50.40			2', 2''	1
H'	4.46	15.19(2'')	1, 2''	2	1, 3, 4
H''	1.57	15.19(2')	1, 2'	2	1, 3, 4
3 C	305.85				1, 2', 2'', 5
4 C	155.92				2', 2'', 6
5 C	121.12			5	
H	7.50		6	5	3, 7, 13
6 C	130.09			6	8
H	7.79		5	6	4, 7, 8, 12
7 C	133.17				5, 6, 8, 9, 11
8 C	128.94			8	6, 10
H	7.79		9	8	6, 7, 10, 12
9 C	128.33			9	11
H	7.71		8, 10	9	7, 11
10 C	129.75			10	8
H	7.57		9, 11	10	8, 12
11 C	131.23			11	9
H	7.97		10	11	7, 9, 13
12 C	134.90				6, 8, 10
13 C	104.46				5, 11
14 C	183.06				15, 28

Atom	δ (ppm)	J	COSY	HSQC	HMBC
15 C	126.26			15	28
H	7.37		28	15	14, 28
16 C	134.87				18, 21, 23, 25
17 C	149.34				18, 19, 20, 22
18 C	29.43			18	19, 20, 21
H	4.13		19, 20	18	16, 17, 19, 20, 21
19 C	27.72			19	18, 20
H3	1.18		18	19	17, 18, 20
20 C	22.38			20	18, 19
H3	1.58		18	20	17, 18, 19
21 C	126.05			21	18, 23
H	7.54			21	16, 18, 23
22 C	131.94			22	
H	7.64			22	17, 24
23 C	124.50			23	21, 25
H	7.41			23	16, 21, 25
24 C	145.71				22, 25, 26, 27
25 C	29.35			25	23, 26, 27
H	2.35		26, 27	25	16, 23, 24, 26, 27
26 C	28.08			26	25, 27
H3	1.11		25	26	24, 25, 27
27 C	22.72			27	25, 26
H3	1.55		25	27	24, 25, 26

Atom	δ (ppm)	J	COSY	HSQC	HMBC
28 C	127.77			28	15
H	7.22		15	28	14, 15
29 C	136.79				31, 34, 36, 38
30 C	147.73				31, 32, 33, 35
31 C	28.84			31	32, 33, 34
H	3.60		31, 32, 33	31	29, 30, 32, 33, 34
32 C	27.01			32	31, 33
H3	1.23		31	32	30, 31, 33
33 C	23.61			33	31, 32
H3	1.54		31	33	30, 31, 32
34 C	125.78			34	31, 36
H	7.45			34	29, 31, 36
35 C	130.46			35	
H	7.45		36	35	30, 37
36 C	123.33			36	34, 38
H	6.71		35	36	29, 34, 38
37 C	144.90				35, 38, 39, 40
38 C	29.11			38	36, 39, 40
H	1.67		39, 40	38	29, 36, 37, 39, 40
39 C	25.51			39	38, 40
H3	0.52		38	39	37, 38, 40
40 C	19.47			40	38, 39
H3	-0.42		38	40	37, 38, 39

Table S2. NMR analysis of the *trans*-configured Ru carbene *trans*-**5g**; this dataset was recorded on a 600 MHz spectrometer in CD_2Cl_2.

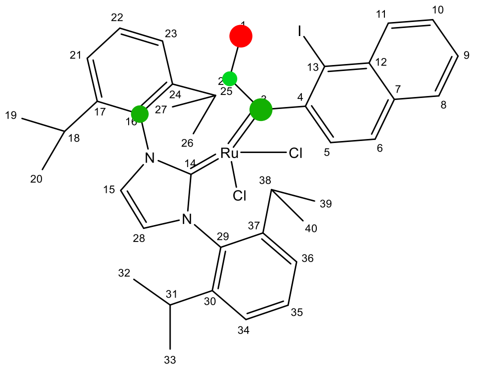

$31		trans-$31		Δδ	
Atom	δ (ppm)	Atom	δ (ppm)	13C	1H
1 C	8,51	1 C	11,3	-2,79	
H3	0,89	H3	1,07		-0,18
2 C	51,29	2 C	50,4	0,89	
H'	4,73	H'	4,46		0,27
H''	1,91	H''	1,57		0,34
3 C	308,54	3 C	305,85	2,69	
4 C	155,69	4 C	155,92	-0,23	
5 C	121,01	5 C	121,12	-0,11	
H	7,47	H	7,5		-0,03
6 C	130,36	6 C	130,09	0,27	
H	7,81	H	7,79		0,02
7 C	133,15	7 C	133,17	-0,02	
8 C	128,91	8 C	128,94	-0,03	
H	7,79	H	7,79		0
9 C	128,32	9 C	128,33	-0,01	
H	7,69	H	7,71		-0,02
10 C	129,71	10 C	129,75	-0,04	
H	7,56	H	7,57		-0,01
11 C	131,14	11 C	131,23	-0,09	
H	7,92	H	7,97		-0,05
12 C	134,9	12 C	134,9	0	
13 C	104,55	13 C	104,46	0,09	
14 C	183,13	14 C	183,06	0,07	
15 C	126,2	15 C	126,26	-0,06	
H	7,4	H	7,37		0,03
16 C	135,44	16 C	134,87	0,57	
17 C	149,5	17 C	149,34	0,16	
18 C	29,53	18 C	29,43	0,1	
H	4,27	H	4,13		0,14
19 C	27,66	19 C	27,72	-0,06	
H3	1,21	H3	1,18		0,03
20 C	22,27	20 C	22,38	-0,11	
H3	1,56	H3	1,58		-0,02
21 C	126,04	21 C	126,05	-0,01	
H	7,56	H	7,54		0,02

$31		trans-$31		Δδ	
Atom	δ (ppm)	Atom	δ (ppm)	13C	1H
22 C	131,94	22 C	131,94	0	
H	7,64	H	7,64		0
23 C	124,54	23 C	124,5	0,04	
H	7,4	H	7,41		-0,01
24 C	145,94	24 C	145,71	0,23	
25 C	29,28	25 C	29,35	-0,07	
H	2,31	H	2,35		-0,04
26 C	28,02	26 C	28,08	-0,06	
H3	1,11	H3	1,11		0
27 C	22,64	27 C	22,72	-0,08	
H3	1,52	H3	1,55		-0,03
28 C	127,83	28 C	127,77	0,06	
H	7,21	H	7,22		-0,01
29 C	136,72	29 C	136,79	-0,07	
30 C	147,75	30 C	147,73	0,02	
31 C	28,77	31 C	28,84	-0,07	
H	3,6	H	3,6		0
32 C	27,03	32 C	27,01	0,02	
H3	1,22	H3	1,23		-0,01
33 C	23,57	33 C	23,61	-0,04	
H3	1,52	H3	1,54		-0,02
34 C	125,75	34 C	125,78	-0,03	
H	7,45	H	7,45		0
35 C	130,43	35 C	130,46	-0,03	
H	7,45	H	7,45		0
36 C	123,38	36 C	123,33	0,05	
H	6,71	H	6,71		0
37 C	144,92	37 C	144,9	0,02	
38 C	29,24	38 C	29,11	0,13	
H	1,62	H	1,67		-0,05
39 C	25,65	39 C	25,51	0,14	
H3	0,52	H3	0,52		0
40 C	19,24	40 C	19,47	-0,23	
H3	-0,44	H3	-0,42		-0,02

Table S3. Comparison of the NMR data of *cis*- and *trans*-**5g**

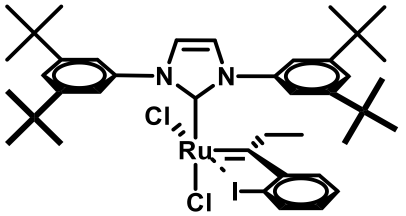

Ruthenium Complex 5h. Prepared analogously from the ruthenium cymene complex **1g** (91 mg, 121 µmol) and alkyne **2b** (45.3 mg, 182 µmol). The mixture was loaded on a silica pad, eluting with CH_2Cl_2/Et_2O (1:1, 30 mL). A brown-yellow band was collected and a dark residue stayed on top of the silica pad. The combined product-containing fractions were concentrated, the residue was triturated with pentane and the supernatant was filtered off. The resulting brown solid was re-dissolved in *tert*-butyl methyl ether (2 mL) and the product was precipitated with pentane (30 mL) at –20° C. The supernatant was filtered off and the filtrate was stored at –20° C overnight to provide a second crop of product of the same purity. Drying in vacuum provided the title compound as a brown solid (47 mg, 45%). ^{1}H NMR (400 MHz, CD_2Cl_2) δ 8.04 (s, 2H), 7.72 (t, *J* = 1.9 Hz, 1H), 7.65 (t, *J* = 1.7 Hz, 1H), 7.55 (t, *J* = 1.8 Hz, 1H), 7.47 (d, *J* = 2.0 Hz, 1H), 7.42 (dd, *J* = 7.8, 1.1 Hz, 1H), 7.28 (td, *J* = 7.5, 1.4 Hz, 1H), 7.14 (td, *J* = 7.6, 7.2, 1.2 Hz, 1H), 6.98 (d, *J* = 2.0 Hz, 1H), 6.71 (dd, *J* = 7.9, 1.4 Hz, 1H), 6.19 (t, *J* = 1.9 Hz, 1H), 5.30 – 5.23 (m, 1H), 2.27 (dq, *J* = 15.3, 7.7 Hz, 1H), 1.47 (s, 18H), 1.42 (s, 9H), 1.22 (s, 9H), 0.84 (t, *J* = 7.5 Hz, 3H). ^{13}C NMR (101 MHz, CD_2Cl_2) δ 316.4, 175.1, 159.5, 154.8, 152.8, 152.1, 140.3, 139.1, 134.7, 130.4, 129.2, 126.8, 125.0, 124.6, 123.9, 123.4, 122.1, 119.9, 119.5, 95.6, 53.3, 36.1, 35.9, 35.2, 31.9, 31.8, 31.6, 31.6, 7.6. IR (film) ṽ 2959, 2904, 1590, 1604, 1477, 1437, 1363, 1325, 1247, 709 cm^{-1}. HRMS (ESI$^+$) calcd. for $C_{40}H_{53}Cl_2IN_2Ru$ [M–Cl]$^+$: 825.19800; found: 825.19821.

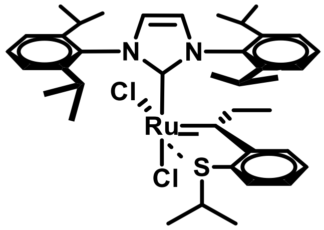

Ruthenium Complex 5i. Prepared analogously from the ruthenium cymene complex **1b** (348 mg, 500 µmol) and alkyne **2c** (128 mg, 675 µmol). The reaction mixture was loaded on a silica pad, eluting with pentane/acetone (1:1, 50 mL). A green band was collected and a dark residue stayed on top of the silica pad. The product-containing fractions were concentrated, the residue was re-dissolved in CH_2Cl_2 (4 mL) and the product precipitated with pentane (50 mL) at 0° C. The supernatant was filtered off and the green residue was washed with pentane (10 mL). Drying in high vacuum provided the title complex as a pale green powder (323 mg, 86%). ^{1}H NMR (400 MHz, CD_2Cl_2) δ 7.57 (t, *J* = 7.7 Hz, 1H), 7.53 – 7.46 (m, 3H), 7.44 – 7.37 (m, 3H), 7.36 – 7.31 (m, 1H), 7.26 (ddd, *J* = 8.1, 6.2, 2.1 Hz, 1H), 7.20 (d, *J* = 2.0 Hz, 1H), 7.13 (d, *J* = 1.9 Hz, 1H), 6.70 (dd, *J* = 5.8, 3.4 Hz, 1H), 4.64 (dq, *J* = 13.1, 7.6 Hz, 1H), 4.12 (hept, *J* = 6.7 Hz, 1H), 3.65 (hept, *J* = 6.8 Hz, 1H), 3.26 (hept, *J* = 6.8 Hz, 1H), 2.38 (hept, *J* = 6.9 Hz, 1H), 1.71 (dq, *J* = 13.0, 7.5 Hz, 1H), 1.65 – 1.58 (m, 1H), 1.54 (dd, *J* = 6.8, 4.0 Hz, 6H), 1.49 (d, *J* = 6.6 Hz, 3H), 1.17 (d, *J* = 6.8 Hz, 3H), 1.15 (d, *J* = 6.9 Hz, 3H), 1.11 (d, *J* = 6.8 Hz, 3H), 0.89 (d, *J* = 7.0 Hz, 3H), 0.88 (t, *J* = 7.6 Hz, 3H), 0.80 (d, *J* = 6.7 Hz, 3H), 0.58 (d, *J* = 6.7 Hz, 3H), -0.13 (d, *J* = 6.6 Hz, 3H). ^{13}C NMR (101 MHz, CD_2Cl_2) δ 314.7, 182.9, 155.4, 149.1, 148.1, 147.3, 145.2, 137.4, 136.9, 135.4, 131.6, 131.5, 130.5, 130.3, 129.0, 127.7, 125.9, 125.8, 125.7, 124.6, 123.2, 47.0, 39.2, 29.9, 29.5,

29.2, 28.8, 27.9, 27.5, 27.4, 26.5, 23.7, 23.0, 22.8, 22.3, 21.5, 19.6, 8.5. IR (film) ṽ 2962, 1462, 1395, 1320, 1265, 802, 760, 702 cm^{-1}. HRMS (ESI+) calcd. for $C_{39}H_{52}Cl_2N_2RuS$ $[M–Cl]^+$: 717.25777; found: 717.25759. Anal. calcd. for $C_{39}H_{52}Cl_2N_2RuS$: C, 62.22; H, 6.96; N, 3.72; found: C, 62.27; H, 6.89; N, 3.65.

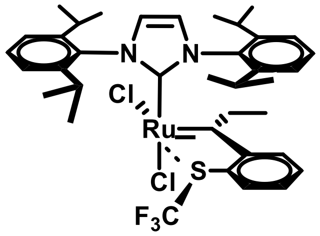

Ruthenium Complex 5j. Prepared analogously from the ruthenium cymene complex **1b** (183 mg, 263 µmol) and alkyne **2d** (85.2 mg, 394 µmol) with a reduced reaction time of 3 h. The crude material was purified by flash chromatography (silica, pentane/*tert*-butyl methyl ether, 1:1) to provide a green solid (96 mg, 47%). ^{1}H NMR (400 MHz, CD_2Cl_2) δ 7.72 – 7.69 (m, 1H), 7.67 – 7.58 (m, 2H), 7.51 – 7.37 (m, 6H), 7.30 (d, *J* = 1.9 Hz, 1H), 7.22 (d, *J* = 1.9 Hz, 1H), 6.73 (p, *J* = 4.0 Hz, 1H), 4.40 (dq, *J* = 12.8, 7.6 Hz, 1H), 3.95 (hept, *J* = 6.4 Hz, 1H), 3.55 (hept, *J* = 6.8 Hz, 1H), 2.39 (hept, *J* = 6.8 Hz, 1H), 1.70 (dq, *J* = 12.8, 7.4 Hz, 1H), 1.64 – 1.52 (m, 1H), 1.52 – 1.50 (m, 9H), 1.21 – 1.16 (m, 6H), 1.14 (d, *J* = 6.7 Hz, 3H), 0.88 (t, *J* = 7.5 Hz, 3H), 0.61 (d, *J* = 6.7 Hz, 3H), -0.08 (d, *J* = 6.7 Hz, 3H). ^{13}C NMR (101 MHz, CD_2Cl_2) δ 315.1, 179.9, 154.1, 148.8, 148.1, 146.5, 144.6, 136.4, 134.1, 132.4, 132.2, 131.1, 131.1, 130.7, 130.6, 128.0, 126.1, 126.1, 126.0, 124.5, 123.2, 122.8, 119.7, 47.0, 30.3, 29.7, 29.3, 29.0, 28.1, 27.6, 27.4, 26.1, 23.7, 22.7, 22.0, 19.5, 8.5. IR (film) ṽ 2964, 2931, 1463, 1399, 1325, 1205, 1155, 1089, 758 cm^{-1}. HRMS (ESI+) calcd. for $C_{37}H_{45}Cl_2F_3N_2RuS$ $[M+Na]^+$: 801.15683; found: 801.15588.

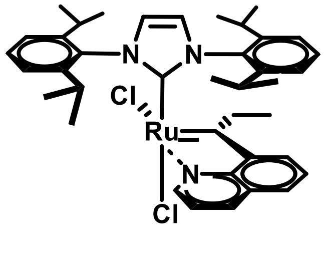

Ruthenium Complex 5k. Prepared analogously from the ruthenium cymene complex **1b** (147 mg, 198 µmol) and alkyne **2h** (49.7 mg, 298 µmol). The crude residue was purified by flash chromatography (silica, pentane/*tert*-butyl methyl ether, 1:5) to provide the title compound as a pale green solid (80 mg, 53%). ^{1}H NMR (600 MHz, CD_2Cl_2) δ 8.14 (dd, *J* = 8.2, 0.9 Hz, 1H), 8.03 (dd, *J* = 8.3, 1.3 Hz, 1H), 7.98 (dd, *J* = 7.4, 1.0 Hz, 1H), 7.74 (t, *J* = 7.8 Hz, 1H), 7.67 (dd, *J* = 7.8, 1.5 Hz, 1H), 7.50 (t, *J* = 7.7 Hz, 1H), 7.48 – 7.41 (m, 2H), 7.34 – 7.28 (m, 1H), 7.25 (d, *J* = 1.8 Hz, 1H), 7.18 (d, *J* = 1.9 Hz, 1H), 6.92 (dd, *J* = 7.6, 1.5 Hz, 1H), 6.87 (dd, *J* = 8.3, 5.1 Hz, 1H), 6.51 (dd, *J* = 5.0, 1.3 Hz, 1H), 4.48 (hept, *J* = 6.7 Hz, 1H), 3.88 (dq, *J* = 12.4, 7.7 Hz, 1H), 3.49 (hept, *J* = 6.7 Hz, 1H), 1.65 (d, *J* = 6.7 Hz, 3H), 1.64 – 1.52 (m, 2H), 1.48 (d, *J* = 6.5 Hz, 3H), 1.40 (hept, *J* = 7.0 Hz, 1H), 1.20 (d, *J* = 6.8 Hz, 3H), 1.18 (d, *J* = 6.9 Hz, 3H), 0.95 (d, *J* = 6.8 Hz, 3H), 0.80 (t, *J* = 7.6 Hz, 3H), 0.40 (d, *J* = 6.8 Hz, 3H), 0.03 (d, *J* = 6.8 Hz, 3H), -0.13 (d, *J* = 6.6 Hz, 3H). ^{13}C NMR (151 MHz, CD_2Cl_2) δ 308.7, 187.9, 158.0, 154.5, 150.6, 149.3, 148.3, 145.4, 137.1, 135.5, 132.1, 131.7, 131.6, 130.6, 129.0, 128.0, 126.0, 125.9, 125.4, 124.8, 124.3, 123.3, 113.6, 44.7, 29.5, 29.4, 29.0, 28.5, 27.7, 27.3, 26.9, 26.4, 23.9, 22.2, 21.9, 19.5, 9.1. IR (film) ṽ 2962, 2928, 1462, 1395, 1317, 1267, 829, 803, 784, 759, 732, 702 cm^{-1}. HRMS (ESI+) calcd. for $C_{39}H_{47}Cl_2IN_3Ru$ $[M–Cl]^+$: 694.24965; found: 694.25059.

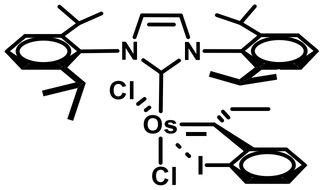

Osmium Complex 6. Prepared analogously from the osmium cymene complex **1h** (272 mg, 347 µmol) and alkyne **2b** (126 mg, 520 µmol). The crude material was purified by flash chromatography (silica, pentane/*tert*-butyl methyl ether, 2:1) to provide a green solid, which was triturated and washed with pentane (2 × 5 mL). The supernatant was filtered off and the residue was dried in high vacuum to provide the title complex as a grey-brown solid (60 mg, 19%). ^{1}H NMR (400 MHz, CD_2Cl_2) δ 7.73 (dd, *J* = 7.9, 1.1 Hz, 1H), 7.56 (t, *J* = 7.7 Hz, 1H), 7.47 – 7.38 (m, 4H), 7.38 – 7.27 (m, 2H), 7.22 (d, *J* = 2.0 Hz, 1H), 7.20 – 7.09 (m, 2H), 6.69 (dd, *J* = 6.5, 2.7 Hz, 1H), 4.26 (dq, *J* = 13.4, 7.6 Hz, 1H), 3.86 (hept, *J* = 6.7 Hz, 1H), 3.57 (hept, *J* = 6.6 Hz, 1H), 2.49 (hept, *J* = 6.4 Hz, 1H), 1.78 (hept, *J* = 6.6 Hz, 1H), 1.59 – 1.56 (m, 1H), 1.55 (d, *J* = 6.6 Hz, 3H), 1.52 (d, *J* = 6.7 Hz, 3H), 1.49 (d, *J* = 6.6 Hz, 3H), 1.20 (d, *J* = 6.8 Hz, 3H), 1.15 (d, *J* = 6.8 Hz, 3H), 1.11 (d, *J* = 6.7 Hz, 3H), 0.72 – 0.60 (m, 6H), –0.03 (d, *J* = 6.7 Hz, 3H). ^{13}C NMR (101 MHz, CD_2Cl_2) δ 244.0, 165.6, 164.6, 149.8, 147.7, 145.9, 145.4, 136.6, 135.5, 134.7, 131.9, 130.3, 130.0, 128.4, 126.6, 125.8, 125.6, 125.5, 124.5, 123.5, 121.7, 106.5, 56.8, 29.8, 29.6, 29.5, 28.9, 28.1, 27.9, 27.1, 25.9, 23.6, 22.9, 22.2, 19.8, 5.9. IR (film) ṽ 2963, 2928, 1461, 1386, 1321, 869, 802, 758, 734 cm^{-1}. HRMS (ESI$^+$) calcd. for $C_{36}H_{45}Cl_2IN_2Os$ $[M{-}Cl]^+$: 859.19253; found: 859.19312.

H/D SCRAMBLING EXPERIMENTS

gem-HYDROGENATION UNDER H_2/D_2-ATMOSPHERE

A 1:1 mixture of H_2 and D_2 was prepared by using a gas-tight 10 mL Hamilton syringe: a gas volume of 5 mL was taken out of a balloon filled with H_2 and of a second balloon filled with D_2.

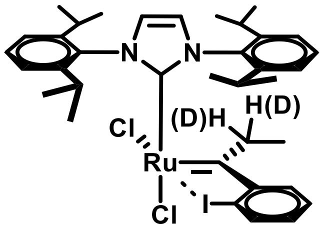

Alkyne **2b** (40.3 mg, 166 µmol) was added to a solution of the ruthenium cymene complex **1b** (78.9 mg, 114 µmol) in CD_2Cl_2 (2 mL) in a flame-dried quartz Schlenk tube under Ar and the tube was closed with a rubber septum. The total volume of 10 mL H_2/D_2 was then flushed through the quartz Schlenk tube using an outlet cannula; this procedure was repeated nine times.

The mixture was then stirred under this H_2/D_2-atmosphere at room temperature overnight with constant irradiation at λ = 365 nm. The mixture was loaded on a silica pad, eluting with CH_2Cl_2/Et_2O (1:1, 30 mL). A brown-yellow band was collected and a dark residue stayed on top of the silica pad. The product-containing fractions were concentrated and the dark brown residue was dissolved in CH_2Cl_2 (2 mL). A brown solid was precipitated upon addition of pentane (30 mL) at room temperature and the supernatant was filtered off. Drying of the residue in high vacuum

afforded a beige-brown solid (56.6 mg, 62%). Identity and composition of the different isotopomers were established by ^{1}H and ^{13}C NMR spectroscopy. The isotopomeric ratio was determined to be $[D_0]:[D_1]:[D_2]$ = 26:42:33.

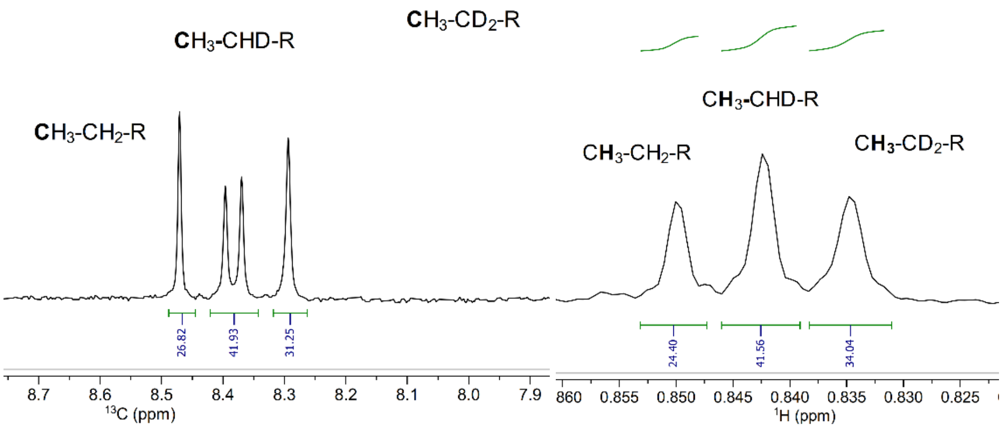

^{2}H Incorporation

	^{13}C	PSYCHE	Average
H_2	26.8 %	24.4 %	25.6 ± 1.7 %
HD	41.9 %	41.6 %	41.7 ± 0.3 %
D_2	31.3 %	34.0 %	32.6 ± 2.0 %

Figure S4. ^{13}C NMR and ^{1}H PSYCHE NMR (Homodecoupled ^{1}H NMR) spectra of the obtained mixture of isotopomers.

HEADSPACE ANALYSIS OF *gem*-HYDROGENATION UNDER H_2/D_2-ATMOSPHERE

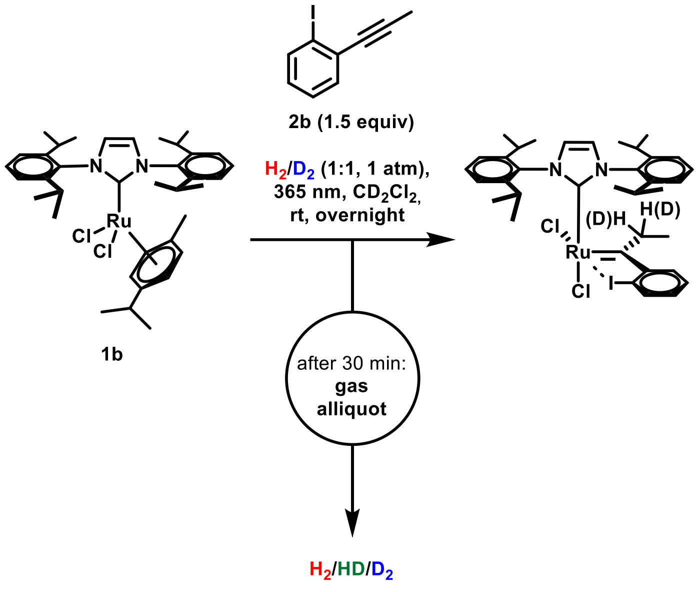

A 1:1 mixture of H_2 and D_2 was prepared by using a gas-tight 10 mL Hamilton syringe: a gas volume of 5 mL was taken out of a balloon filled with H_2 and of a second balloon filled with D_2.
Alkyne **2b** (74.2 mg, 307 µmol) was added to a solution of the ruthenium cymene complex **1b** (142 mg, 204 µmol) in CD_2Cl_2 (4 mL) in a flame-dried quartz Schlenk tube under Ar and the tube was closed with a rubber septum. The total volume of 10 mL H_2/D_2 was then flushed through the quartz Schlenk tube using an outlet cannula; this procedure was repeated nine times.
The mixture was then stirred under H_2/D_2-atmosphere at room temperature for 30 min with constant irradiation at λ = 365 nm. At this point, a gas-tight 1 mL Hamilton syringe was used to take a 1 mL aliquot of the gas in the headspace of the Schlenk tube, which was analyzed by MS (Finnigan MAT SSQ 7000). The sample was directly injected via the GC-inlet of the spectrometer and no column was used. No adequate mass calibration is possible in this mass range: therefore the masses were determined by comparison to an authentic HD sample and helium as references within the measurement. The sample does contain H_2, HD and D_2, see Figure S5.

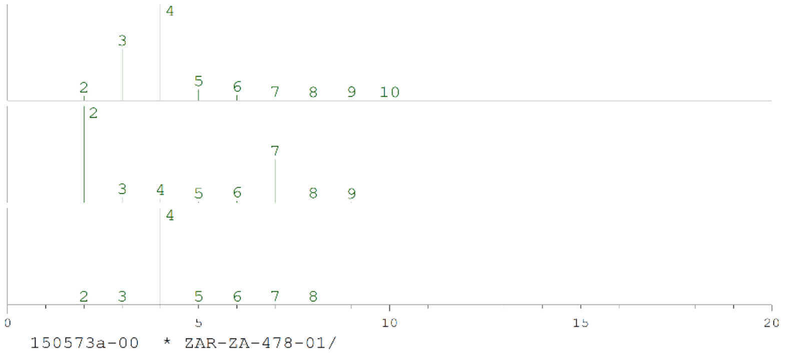

Figure S5. MS analysis of the gas in the headspace: top = headspace sample; middle = H_2/HD (weak) reference sample; bottom = He reference sample

SECOND GENERATION HOVEYDA-GRUBBS CARBENE COMPLEXES FORMED BY CROSS-METATHESIS

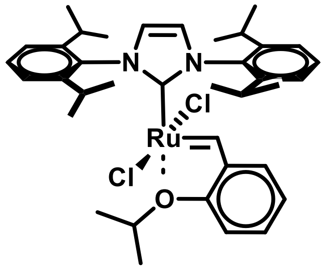

Representative Procedure. Preparation of Complex 3b. Freshly prepared 2-isopropoxystyrene **S2** (303 mg, 1.87 mmol) was added to a suspension of complex **5a** (986 mg, 1.87 mmol) in toluene (40 mL) in a flame-dried Schlenk tube under argon. The mixture was stirred overnight at 80° C (bath temperature). After cooling to room temperature, the mixture was concentrated in high vacuum. The dark brown residue was triturated with pentane at –78° C, the supernatant was filtered off and the residue was washed with pentane (2 × 10 mL). The residue was then purified by flash chromatography (silica, CH_2Cl_2) to provide the title compound as a yellow-brown solid (625 mg, 72%). ^{1}H NMR (400 MHz, CD_2Cl_2) δ 16.50 (s, 1H), 7.63 (t, *J* = 7.8 Hz, 2H), 7.52 (ddd, *J* = 8.8, 7.3, 1.8 Hz, 1H), 7.43 (d, *J* = 7.8 Hz, 4H), 7.19 (s, 2H), 6.97 (dd, *J* = 7.6, 1.7 Hz, 1H), 6.94 – 6.83 (m, 2H), 4.92 (hept, *J* = 6.1 Hz, 1H), 3.08 (hept, *J* = 6.8 Hz, 4H), 1.37 (d, *J* = 6.1 Hz, 6H), 1.19 (d, *J* = 6.7 Hz, 12H), 1.14 (d, *J* = 6.9 Hz, 12H). ^{13}C NMR (101 MHz, CD_2Cl_2) δ 287.1, 177.7, 152.9, 148.8, 145.2, 136.7, 130.9, 129.2, 126.8, 124.4, 123.0, 122.0, 113.5, 75.7, 29.3, 26.7, 23.0, 22.0. IR (film) ṽ 2965, 1474, 1384, 1306, 1265, 1099, 934, 801, 734, 700 cm^{-1}. HRMS (APPI$^+$) calcd. for $C_{37}H_{48}Cl_2N_2ORu$ [M$^+$]: 708.21817; found: 708.21761.

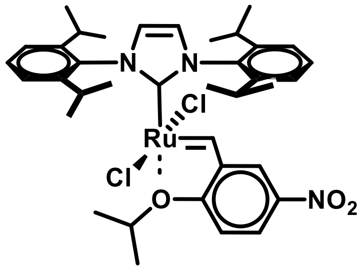

Complex 3c. Prepared analogously from the ruthenium carbene **5a** (83.6 mg, 104 µmol) and 2-isopropoxy-5-nitrostyrene **S3** (20.6 mg, 99.4 µmol). The crude product was triturated with pentane at 0° C, the supernatant was filtered off and the residue was washed with pentane (10 mL). The residue was purified by flash chromatography (silica, pentane/CH_2Cl_2, 4:1) to provide a yellow-brown solid (54.0 mg, 75%). ^{1}H NMR (600 MHz, CD_2Cl_2) δ 16.43 (s, 1H), 8.41 (dd, *J* = 9.0, 2.7 Hz, 1H), 7.79 (d, *J* = 2.7 Hz, 1H), 7.67 (t, *J* = 7.8 Hz, 2H), 7.46 (d, *J* = 7.8 Hz, 4H), 7.22 (s, 2H), 6.97 (d, *J* = 8.9 Hz, 1H), 5.03 (hept, *J* = 6.2 Hz, 1H), 3.04 (hept, *J* = 6.8 Hz, 4H), 1.41 (d, *J* = 6.1 Hz, 6H), 1.19 (d, *J* = 6.7 Hz, 12H), 1.14 (d, *J* = 6.9 Hz, 12H). ^{13}C NMR (151 MHz, CD_2Cl_2) δ 280.7, 174.1, 157.2, 148.7, 144.7, 143.9, 136.3, 131.2, 127.0, 124.6, 124.1, 116.4, 113.5, 78.5, 29.4, 26.7, 23.0, 22.0. IR (film) ṽ 2965, 1523, 1465, 1384, 1317, 1272, 1239, 1092, 918, 737, 702 cm^{-1}. HRMS (APPI$^+$) calcd. for $C_{37}H_{47}Cl_2N_3O_3Ru$ [M$^+$]: 753.20325; found: 753.20234.

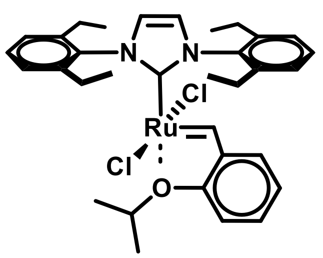

Complex 3d. Prepared analogously from the ruthenium carbene **5d** (49.8 mg, 66.5 µmol) and styrene **S2** (16.2 mg, 99.8 µmol). The dark brown-green crude material was triturated with pentane, the supernatant was filtered off and the residue was washed with pentane (5 mL). All subsequent manipulations were carried out in air with reagent-grade solvents. The dark residue was dissolved in toluene (2 mL), the mixture was filtered through a pad of Celite and the filter cake was washed with toluene (15 mL). The combined filtrates were concentrated to 1/5 of the original volume before pentane (20 mL) was added at room temperature. The precipitated green solid was washed with pentane (10 mL) and dried in high vacuum to provide the title compound as a dark green solid (33 mg, 76%). ^{1}H NMR (600 MHz, CD_2Cl_2) δ 16.52 (s, 1H), 7.57 (t, *J* = 7.7 Hz, 2H), 7.53 (ddd, *J* = 8.2, 7.3, 1.7 Hz, 1H), 7.37 (d, *J* = 7.7 Hz, 4H), 7.21 (s, 2H), 7.01 (dd, *J* = 7.6, 1.6 Hz, 1H), 6.90 (td, *J* = 7.5, 0.8 Hz, 1H), 6.84 (d, *J* = 8.3 Hz, 1H), 4.88 (hept, *J* = 6.1 Hz, 1H), 2.72 (dq, *J* = 15.3, 7.7 Hz, 4H), 2.57 (dq, *J* = 15.1, 7.5 Hz, 4H), 1.28 (d, *J* = 6.1 Hz, 6H), 1.15 (t, *J* = 7.6 Hz, 12H). ^{13}C NMR (151 MHz, CD_2Cl_2) δ 290.6, 176.8, 152.8, 145.5, 144.5, 137.6, 130.6, 129.4, 127.0, 125.9, 123.0, 122.2, 113.5, 75.8, 25.8, 21.6, 15.3. IR (film) ṽ 2964, 1459, 1398, 1310, 1295, 1264, 1214, 1097, 937, 808, 761, 702 cm^{-1}. HRMS (ESI$^+$) calcd. for $C_{33}H_{40}Cl_2N_2ORu$ [M–Cl]$^+$: 617.18672; found: 617.18659.

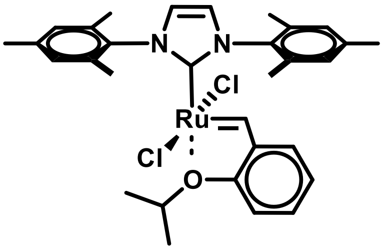

Complex 3e. Prepared analogously from the ruthenium carbene **5e** (52.1 mg, 72.3 µmol) and styrene **S2** (17.6 mg, 109 µmol). The crude material was purified by flash chromatography (silica, pentane/CH_2Cl_2, 1:1) to provide the title compound as a green solid (17.2 mg, 38%). ^{1}H NMR (600 MHz, CD_2Cl_2) δ 16.66 (d, *J* = 0.8 Hz, 1H), 7.56 (ddd, *J* = 8.3, 7.4, 1.6 Hz, 1H), 7.15 (s, 2H), 7.13 (s, 4H), 7.05 (dd, *J* = 7.6, 1.6 Hz, 1H), 6.94 (td, *J* = 7.5, 0.8 Hz, 1H), 6.86 (d, *J* = 8.3 Hz, 1H), 4.92 (hept, *J* = 6.1 Hz, 1H), 2.46 (s, 6H), 2.24 (s, 12H), 1.31 (d, *J* = 6.1 Hz, 6H). ^{13}C NMR (151 MHz, CD_2Cl_2) δ 292.0, 175.7, 152.7, 145.9, 140.2, 138.5, 136.5, 129.5, 129.4, 125.4, 123.1, 122.3, 113.6, 75.8, 21.5, 21.5, 19.4. The spectral data are in good agreement with those reported in the literature.[15]

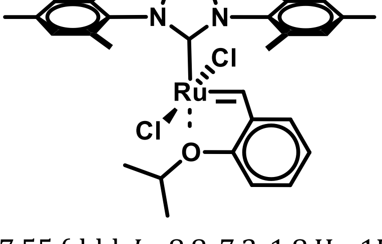

Complex 3f. Prepared analogously from the ruthenium carbene **5f** (54.0 mg, 74.7 µmol) and styrene **S2** (18.9 mg, 116 µmol); the crude material was purified by flash chromatography (silica, pentane/CH_2Cl_2, 4:1) to provide the title compound as a green solid (23.7 mg, 49%). ^{1}H NMR (400 MHz, CD_2Cl_2) δ 16.51 (s, 1H), 7.55 (ddd, *J* = 8.8, 7.3, 1.8 Hz, 1H), 7.07 (s, 4H), 6.96 (dd, *J* = 7.5, 1.8 Hz, 1H), 6.91 (td, *J* = 7.4, 0.8 Hz, 1H), 6.84 (d, *J* = 8.3 Hz, 1H), 4.88 (hept, *J* = 6.2 Hz, 1H), 4.16 (s, 4H), 2.44 (s, 18H), 2.41 (s, 6H). ^{13}C

NMR (101 MHz, CD_2Cl_2) δ 296.1, 211.3, 152.6, 145.7, 139.4, 130.0, 129.8, 122.9, 122.7, 113.5, 75.7, 52.1, 21.4, 21.4, 19.7. The spectral data are consistent with those of a commercially available sample.

CONTROL EXPERIMENT

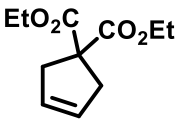

Diethyl cyclopent-3-ene-1,1-dicarboxylate (8). A pressure-Schlenk flask was charged with diethyl diallylmalonate (**7**) (72.5 µL, 300 µmol), complex **5e** (2.2 mg, 3.1 µmol) and CH_2Cl_2 (3 mL). The flask was closed and the mixture stirred at 55°C for 1 h. After cooling to room temperature, the flask was vented, the solvent was removed under reduced pressure, and the residue was purified by flash chromatography (silica, pentane/*tert*-butyl methyl ether 10:1) to provide the title compound as a colorless oil (62.0 mg, 97%). ^1H NMR (400 MHz, $CDCl_3$) δ 5.60 (s, 2H), 4.20 (q, J = 7.1 Hz, 4H), 3.01 (s, 4H), 1.25 (t, J = 7.1 Hz, 6H).

When iodo carbene **5a** (2.5 mg, 3.1 µmol) was used instead, a reaction time of 1.5 h was necessary to provide the desired compound as colorless oil (63.2 mg, 99%).

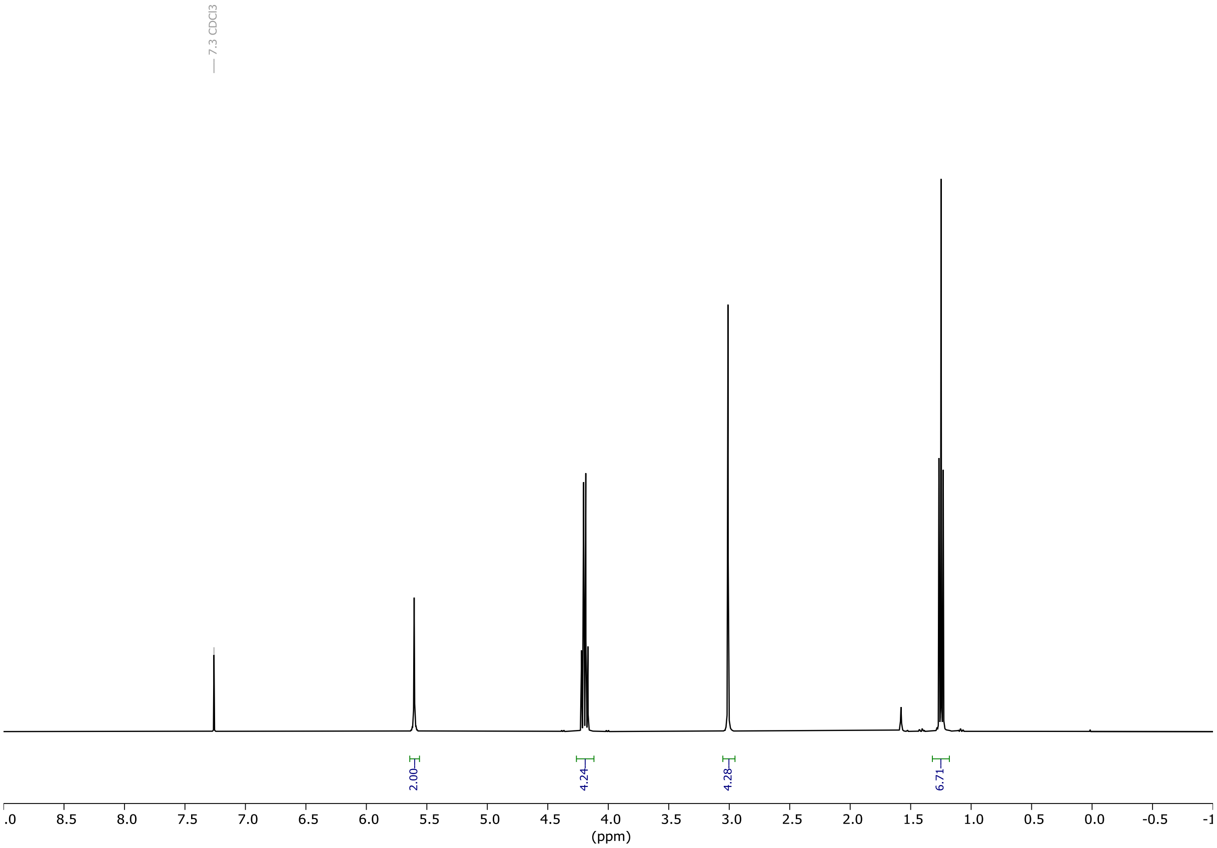

COPIES OF SPECTRA

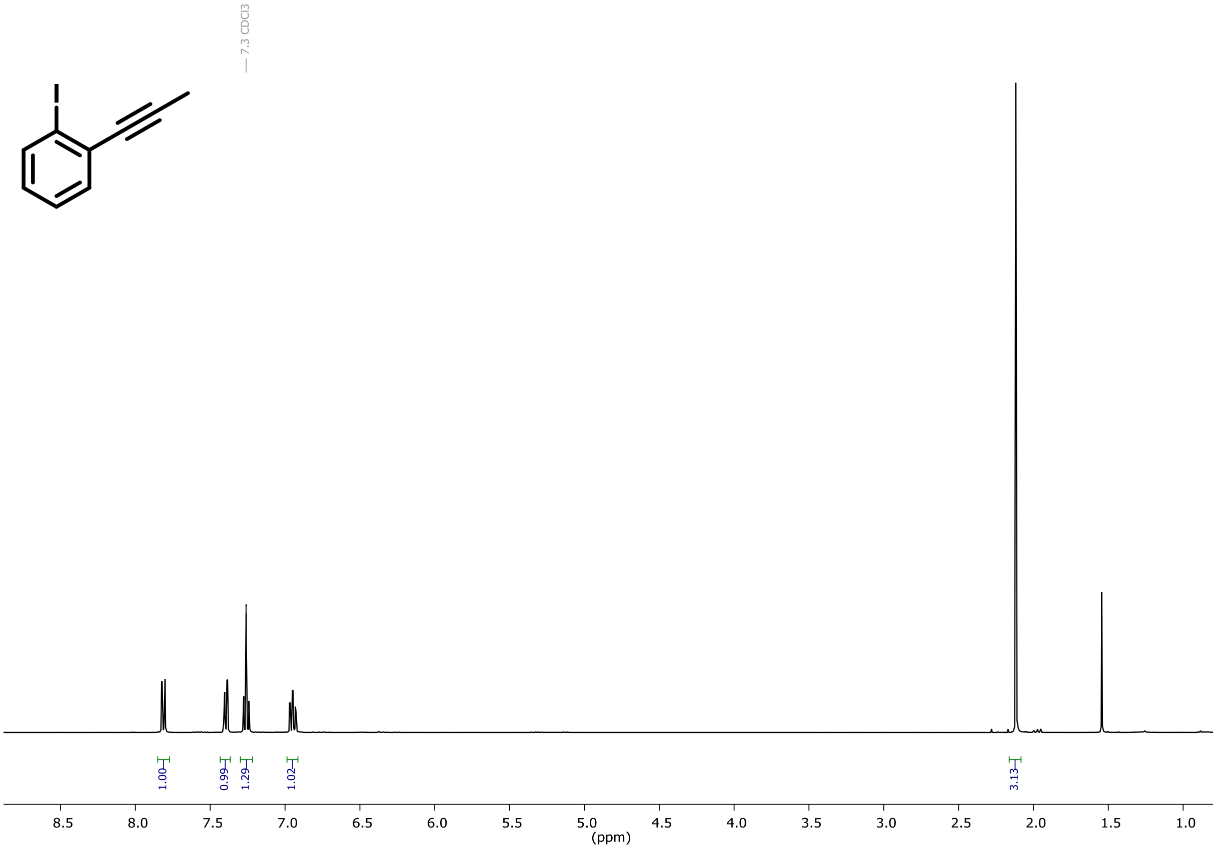

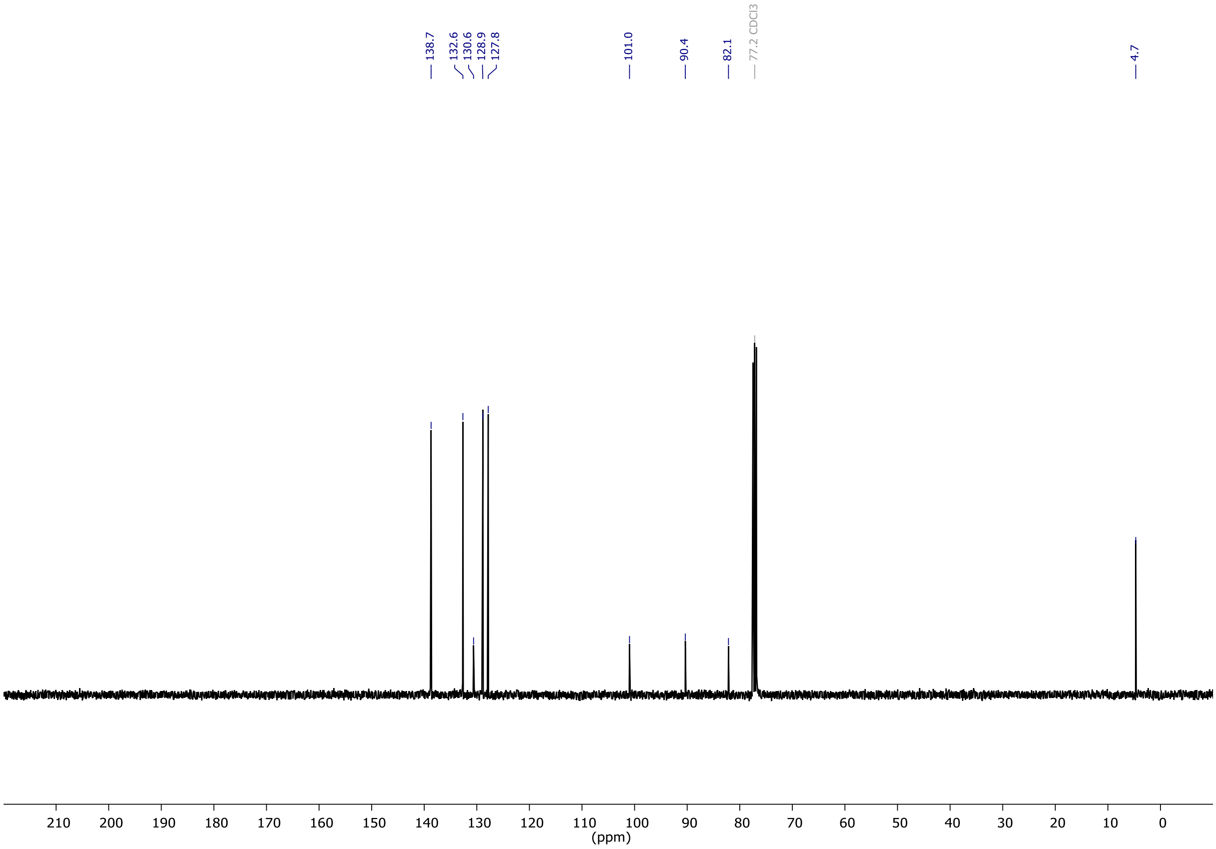

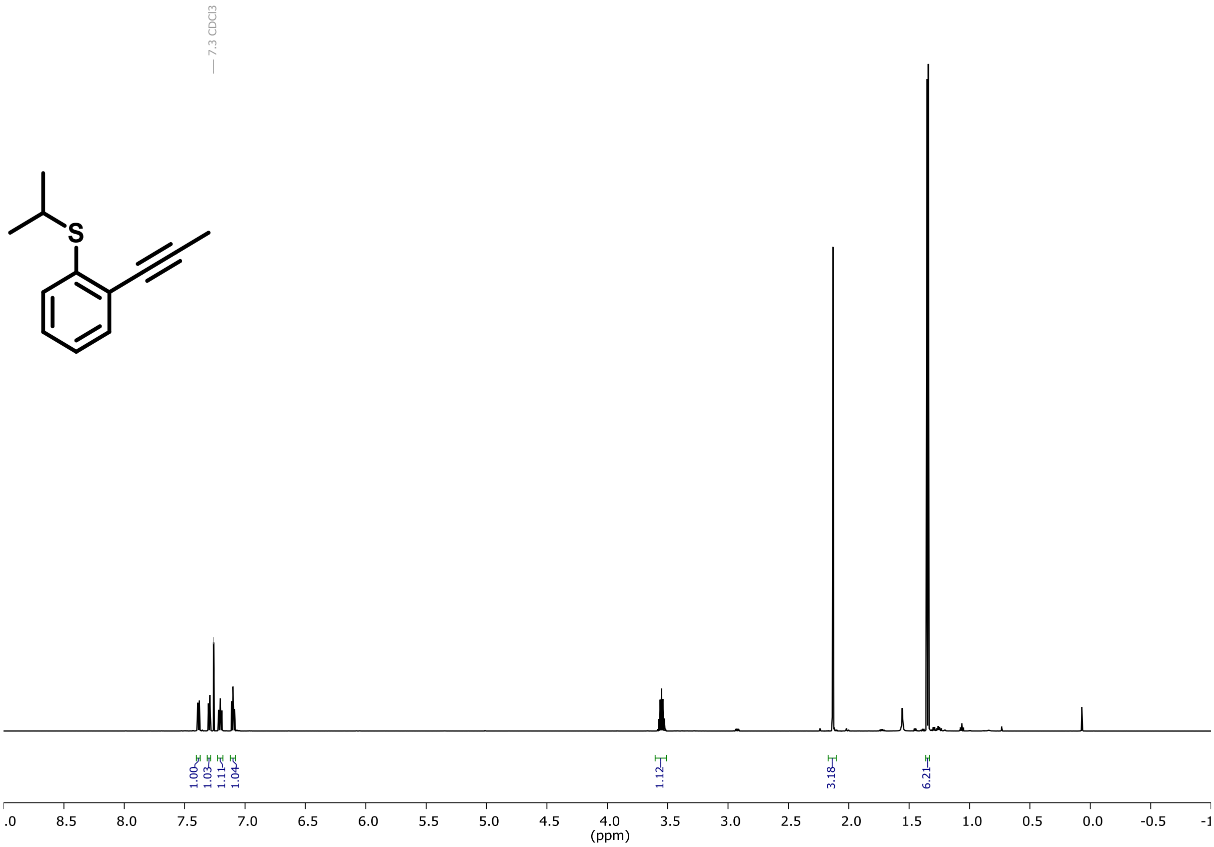
S
7.3 CDCl3
1.00
1.03
1.11
1.04
1.12
3.18
6.21
(ppm)

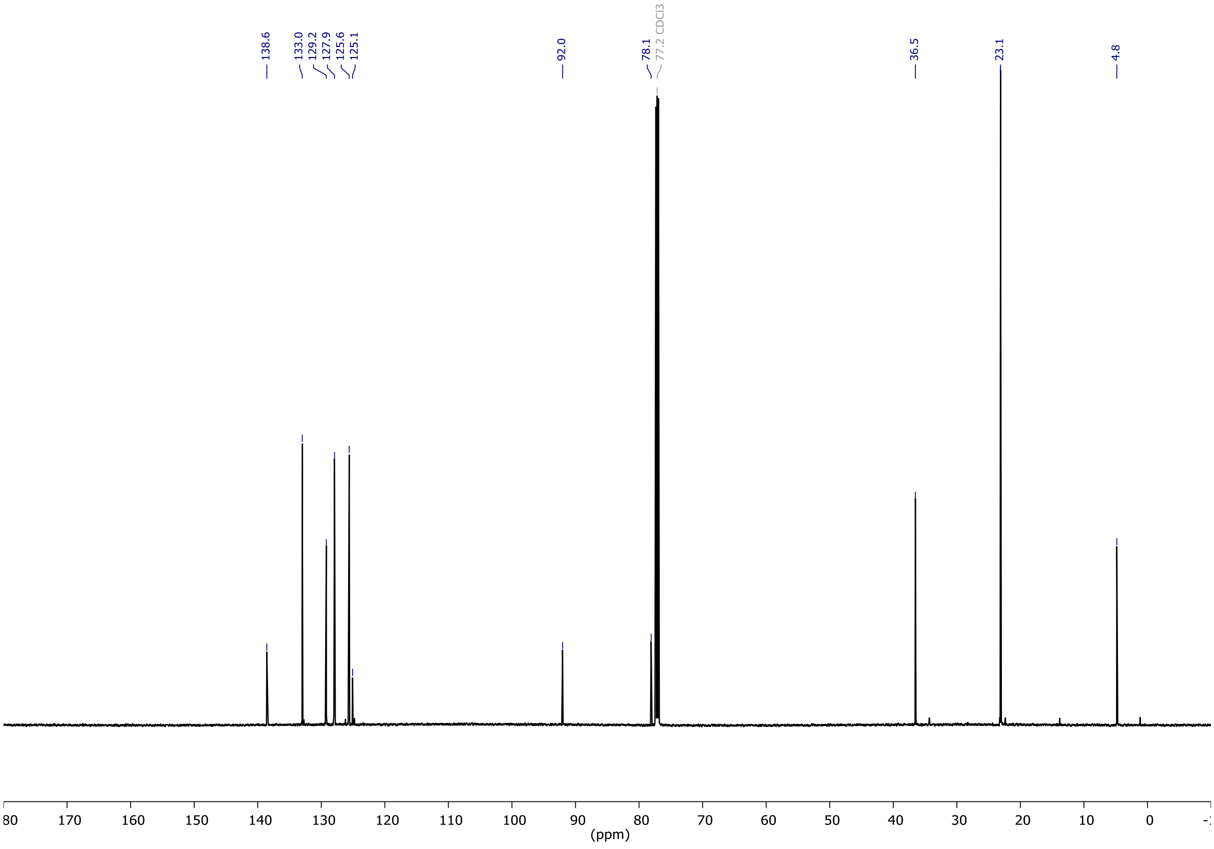
138.6
133.0
129.2
127.9
125.6
125.1
92.0
78.1
77.2 CDCl3
36.5
23.1
4.8
(ppm)

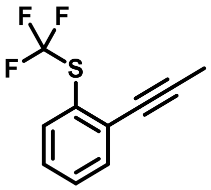
F
F
F
S

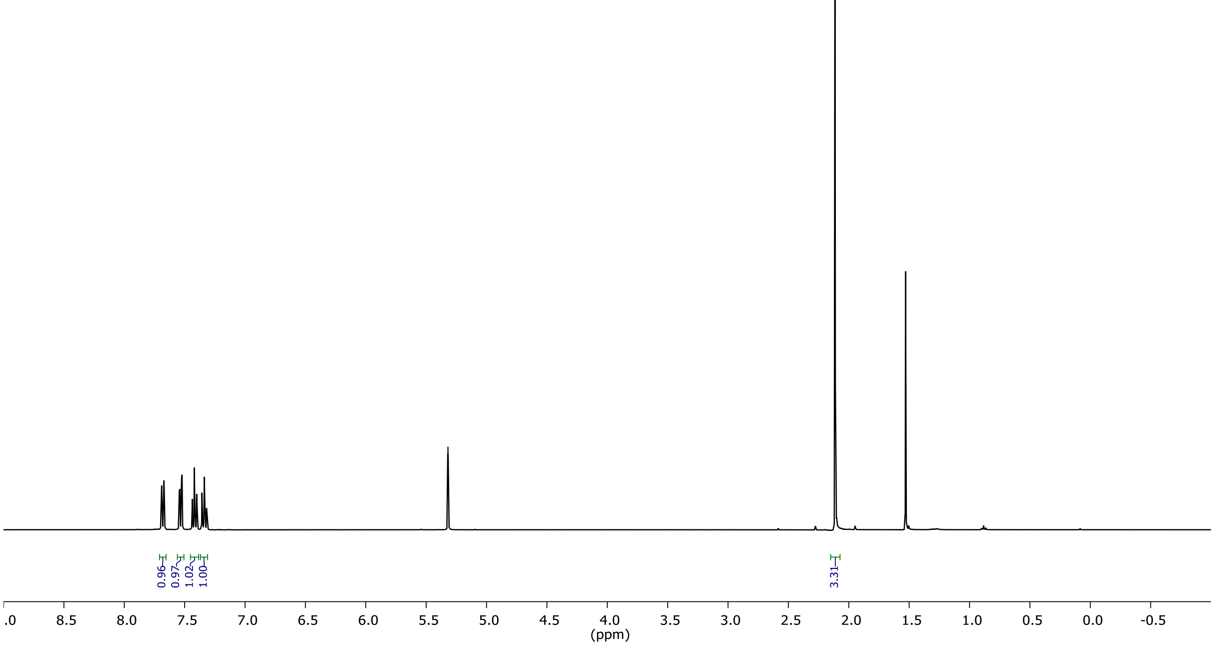
5.3 CD2Cl2
0.96
0.97
1.02
1.00
3.31
(ppm)

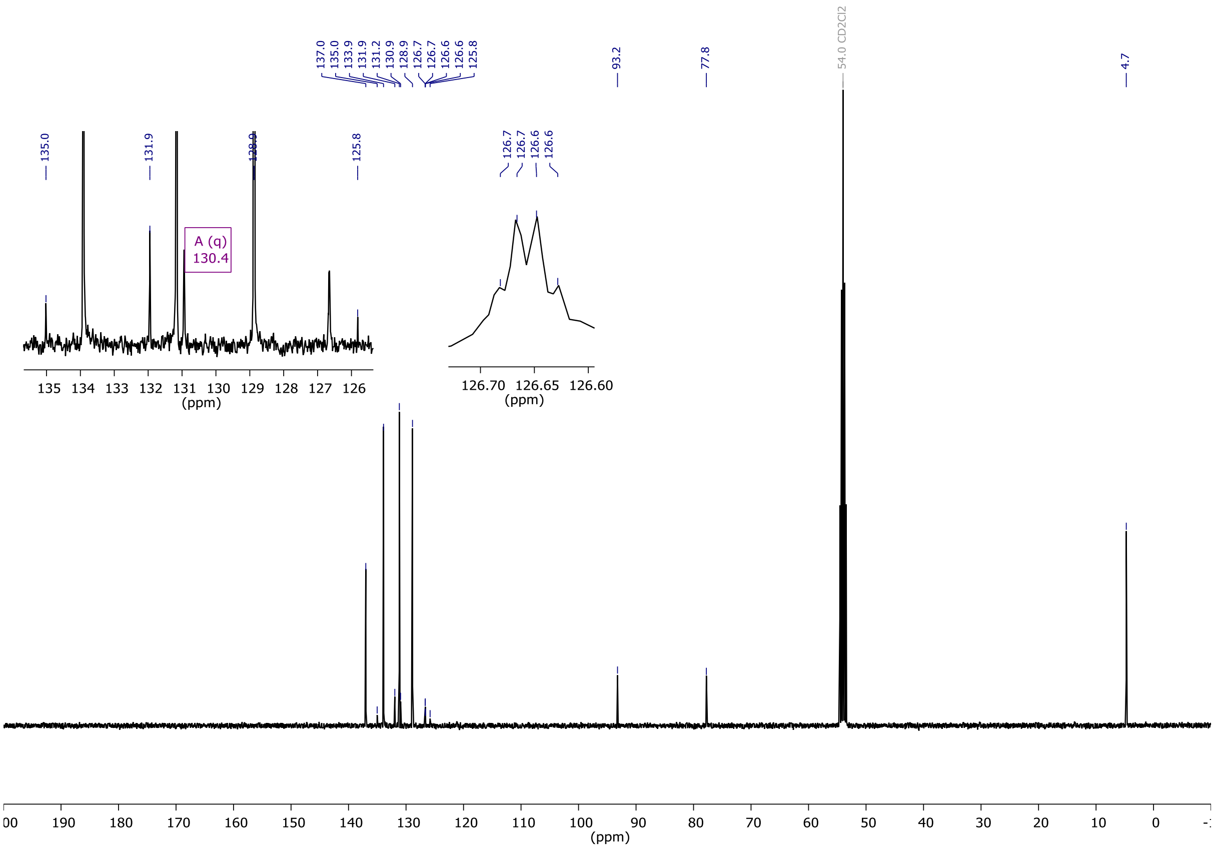
137.0
135.0
133.9
131.9
131.2
130.9
128.9
126.7
126.7
126.6
126.6
125.8
93.2
77.8
54.0 CD2Cl2
4.7
A (q)
130.4
(ppm)

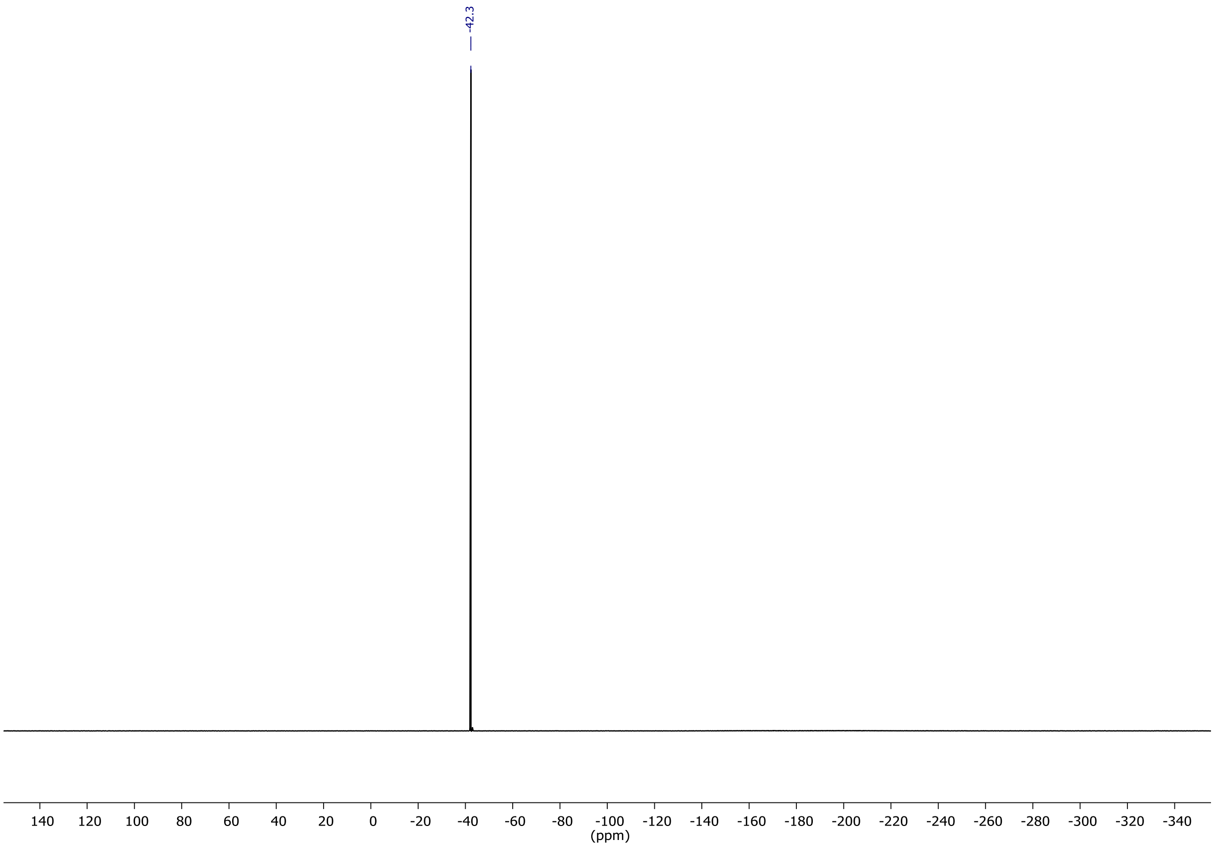
-42.3
140
120
100
80
60
40
20
0
-20
-40
-60
-80
-100
(ppm)
-120
-140
-160
-180
-200
-220
-240
-260
-280
-300
-320
-340

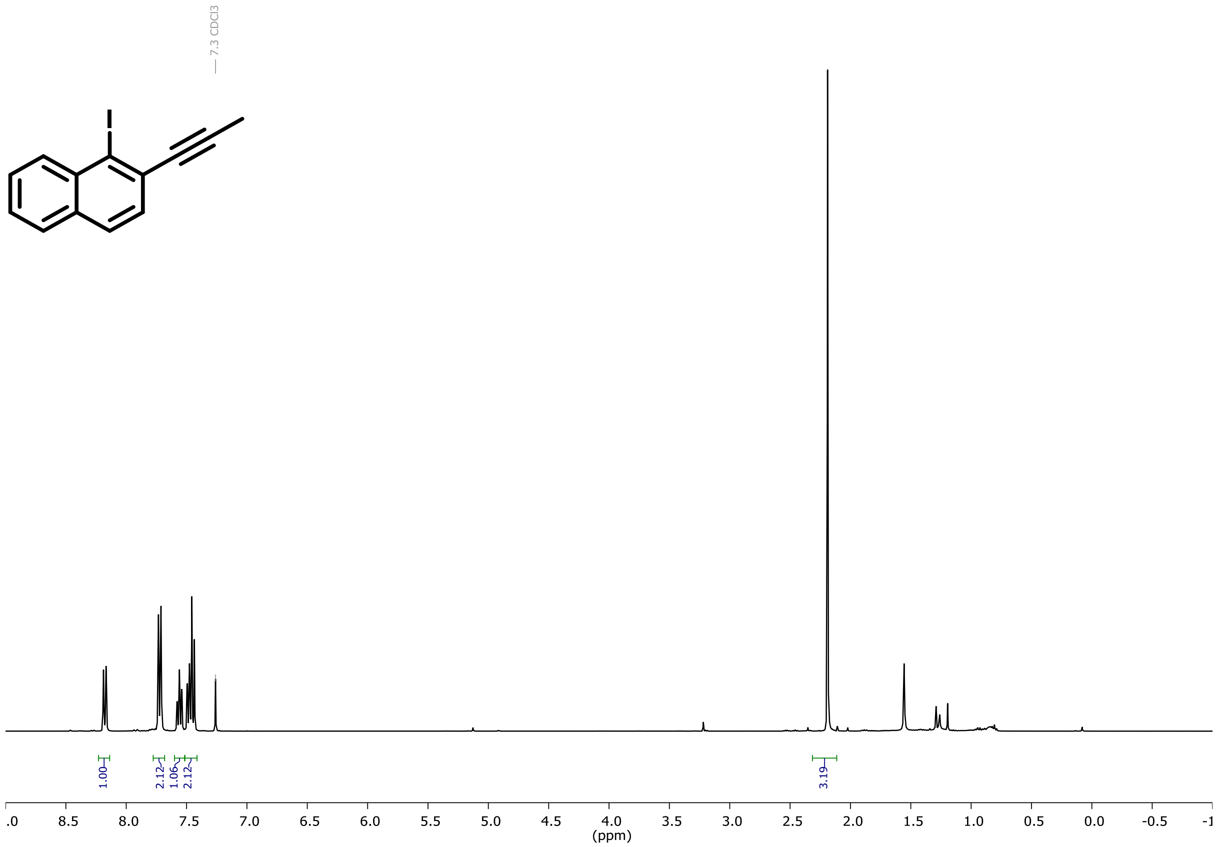
7.3 CDCl3
1.00
2.12
1.06
2.12
3.19
8.5 8.0 7.5 7.0 6.5 6.0 5.5 5.0 4.5 4.0 3.5 3.0 2.5 2.0 1.5 1.0 0.5 0.0 -0.5
(ppm)

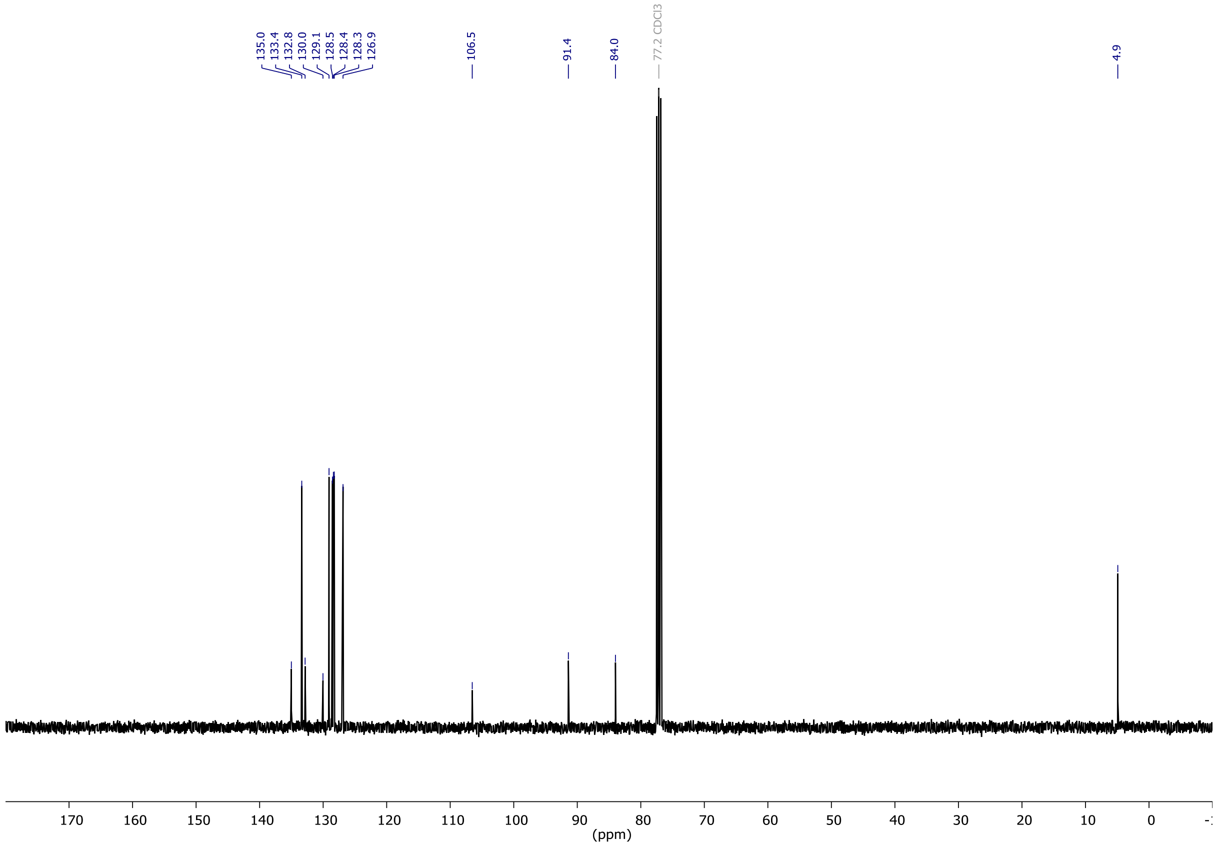
135.0
133.4
132.8
130.0
129.1
128.5
128.4
128.3
126.9
106.5
91.4
84.0
77.2 CDCl3
4.9
170 160 150 140 130 120 110 100 90 80 70 60 50 40 30 20 10 0
(ppm)

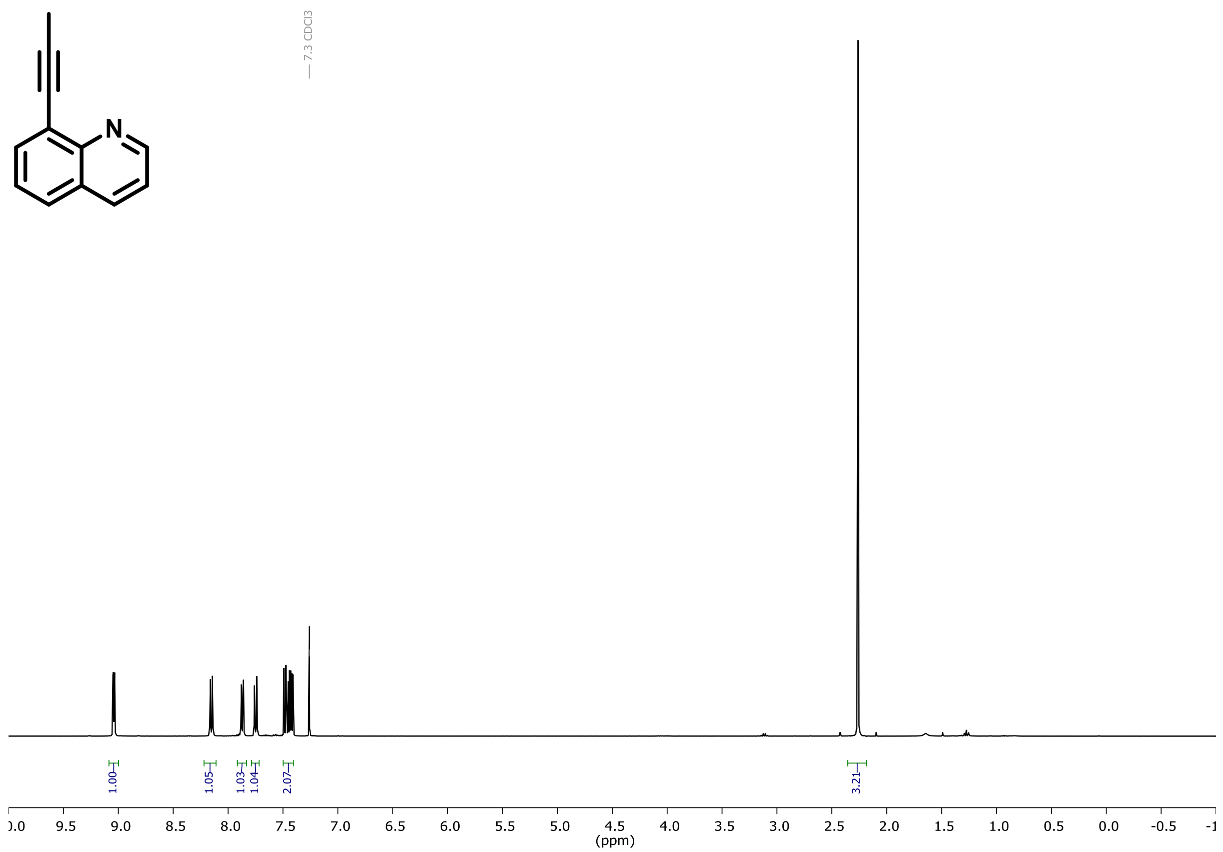
N
7.3 CDCl3
1.00
1.05
1.03
1.04
2.07
3.21
(ppm)

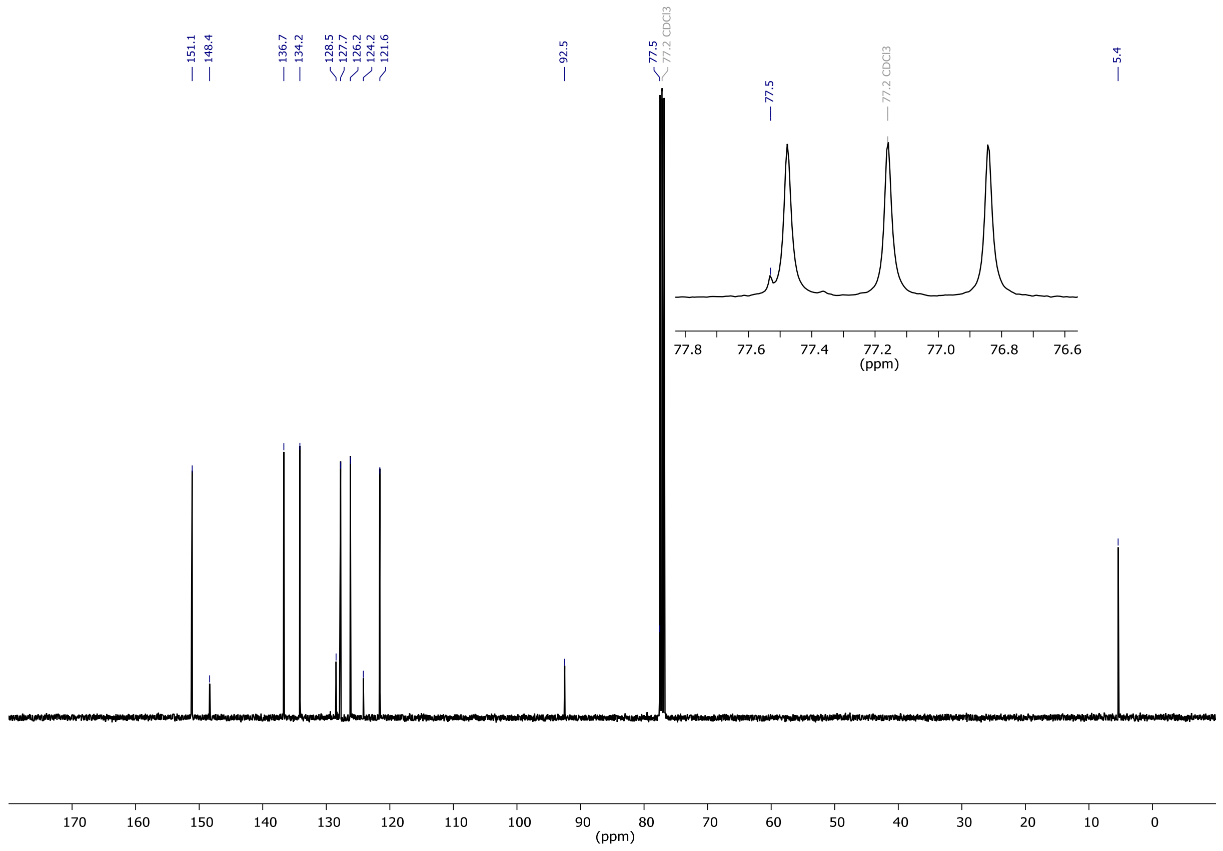
151.1
148.4
136.7
134.2
128.5
127.7
126.2
124.2
121.6
92.5
77.5
77.2 CDCl3
5.4
(ppm)

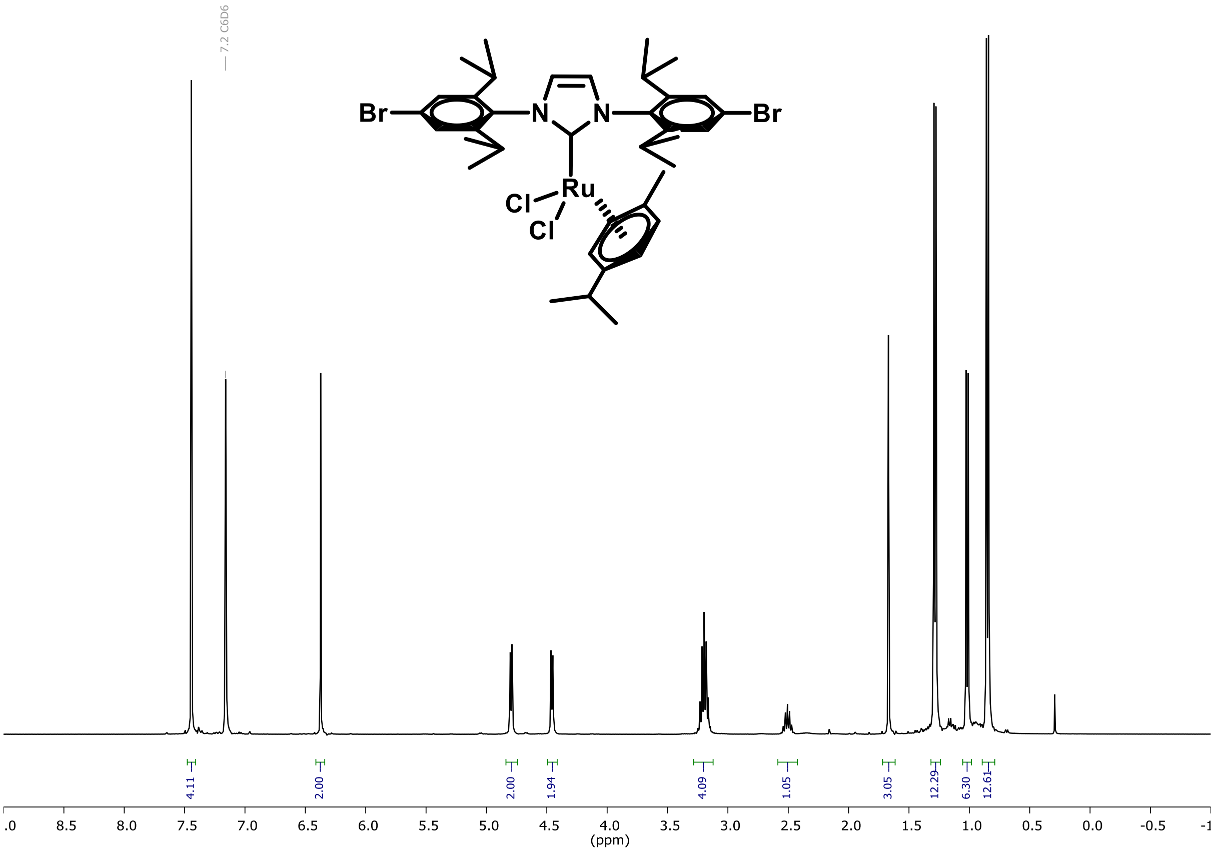
7.2 C6D6
Br
N
N
Br
Ru
Cl
Cl
4.11
2.00
2.00
1.94
4.09
1.05
3.05
12.29
6.30
12.61
(ppm)

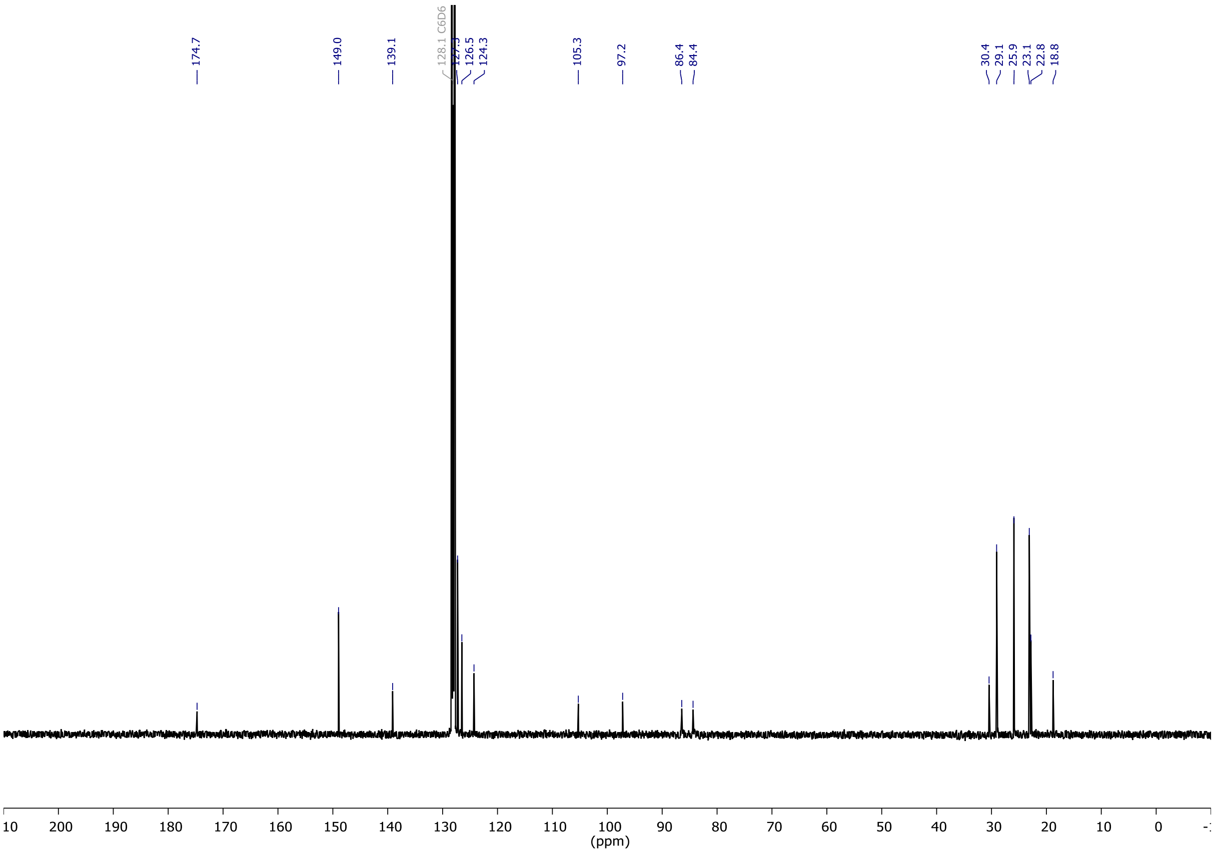
174.7
149.0
139.1
128.1 C6D6
126.5
124.3
105.3
97.2
86.4
84.4
30.4
29.1
25.9
23.1
22.8
18.8
(ppm)

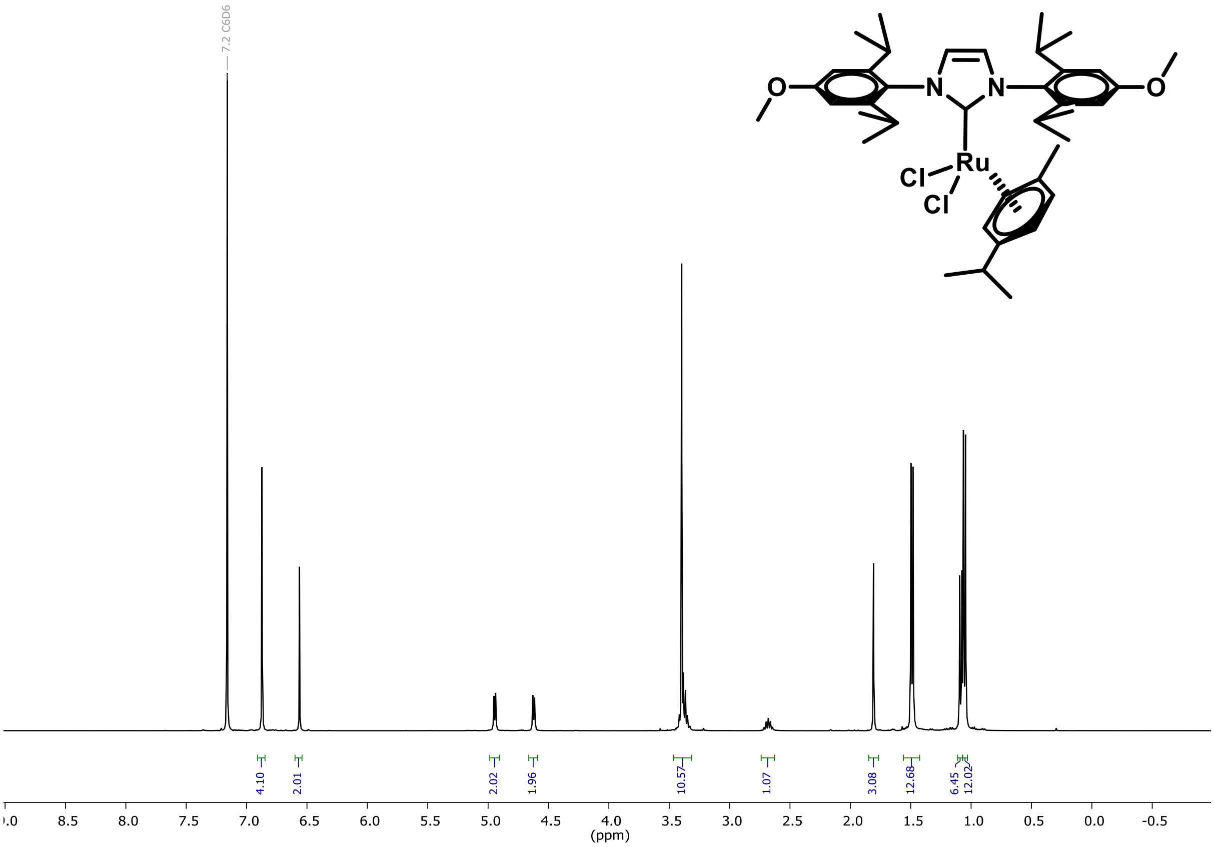
7.2 C6D6
N
N
O
O
Ru
Cl
Cl
4.10
2.01
2.02
1.96
10.57
1.07
3.08
12.68
6.45
12.02
8.5 8.0 7.5 7.0 6.5 6.0 5.5 5.0 4.5 4.0 3.5 3.0 2.5 2.0 1.5 1.0 0.5 0.0 -0.5
(ppm)

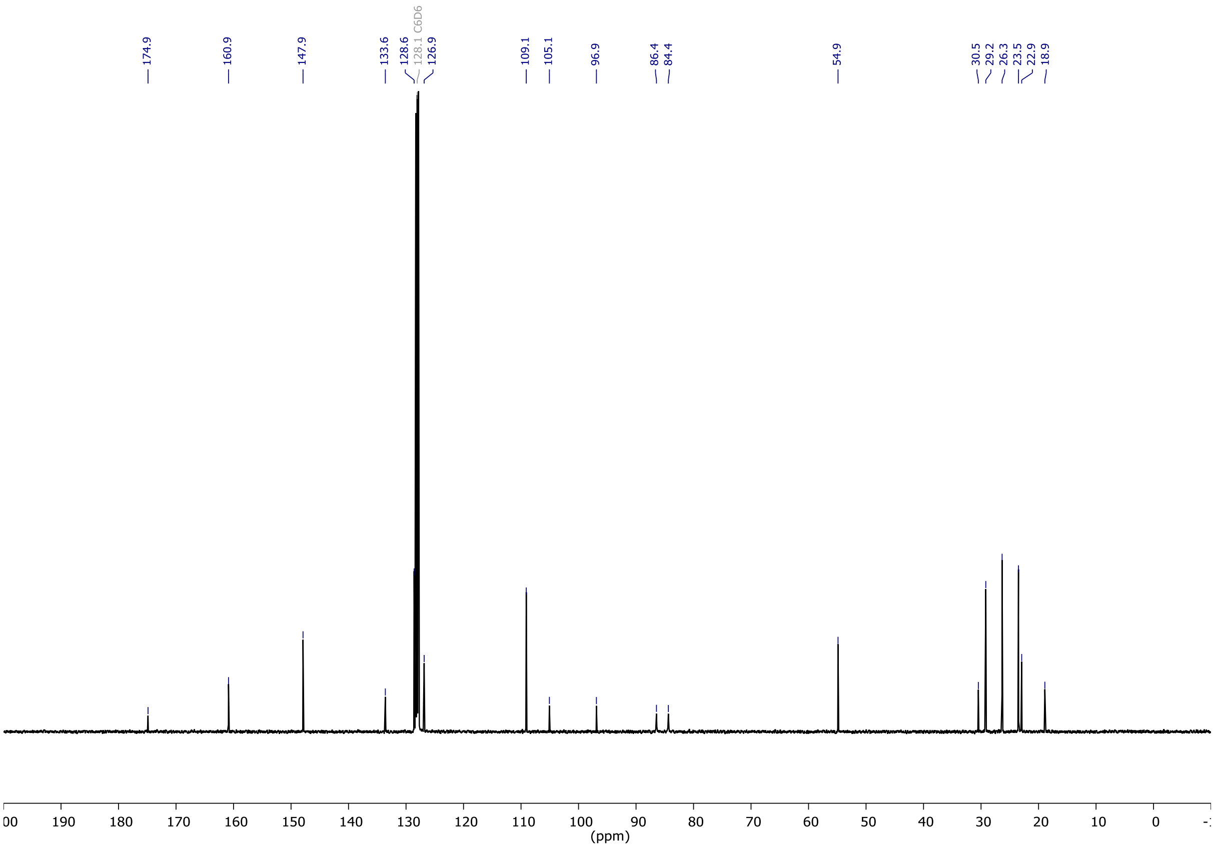
174.9
160.9
147.9
133.6
128.6
128.1 C6D6
126.9
109.1
105.1
96.9
86.4
84.4
54.9
30.5
29.2
26.3
23.5
22.9
18.9
190 180 170 160 150 140 130 120 110 100 90 80 70 60 50 40 30 20 10 0
(ppm)

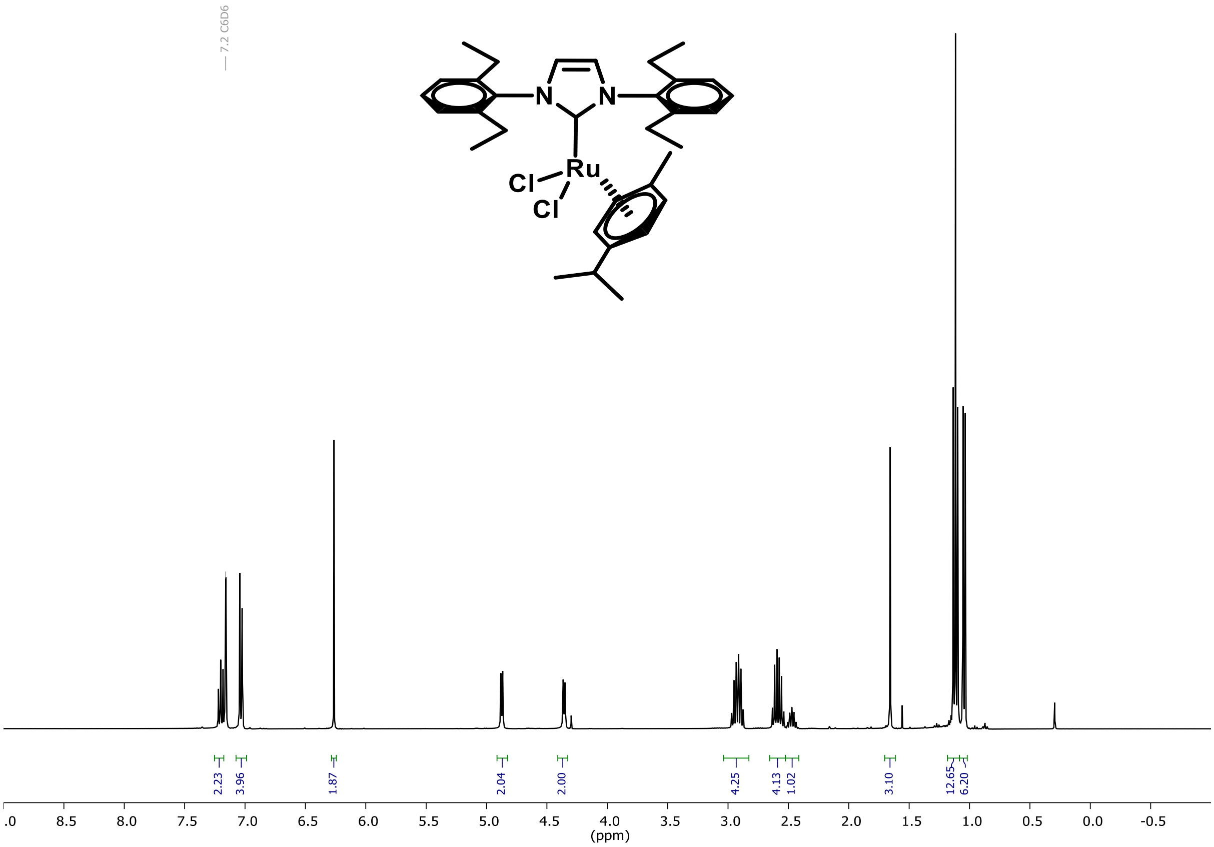
7.2 C6D6
N
N
Ru
Cl
Cl
2.23
3.96
1.87
2.04
2.00
4.25
4.13
1.02
3.10
12.65
6.20
(ppm)

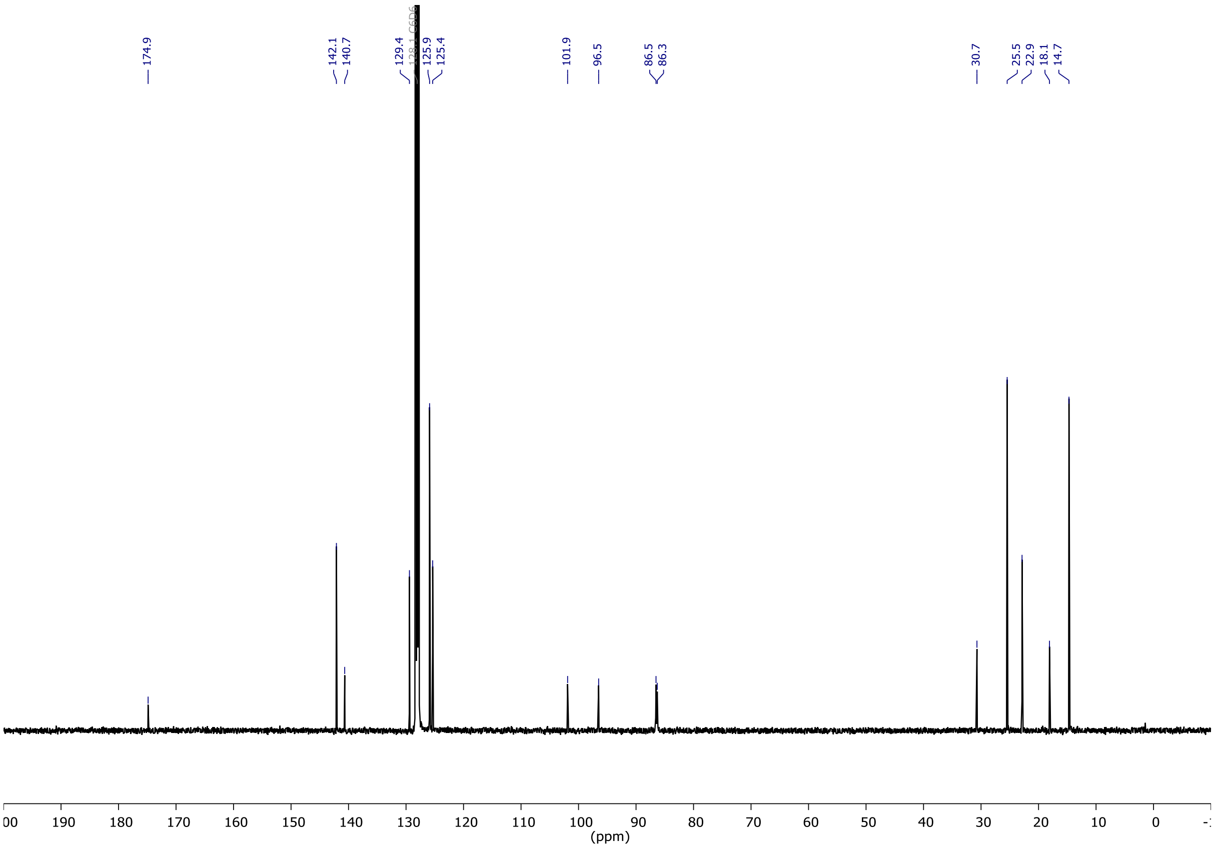
174.9
142.1
140.7
129.4
128.1 C6D6
125.9
125.4
101.9
96.5
86.5
86.3
30.7
25.5
22.9
18.1
14.7
(ppm)

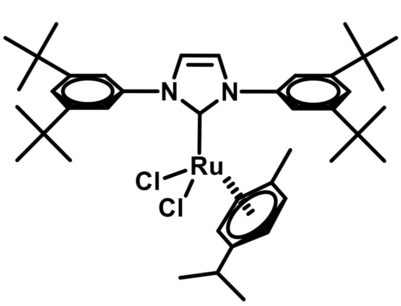
N
N
Ru
Cl
Cl

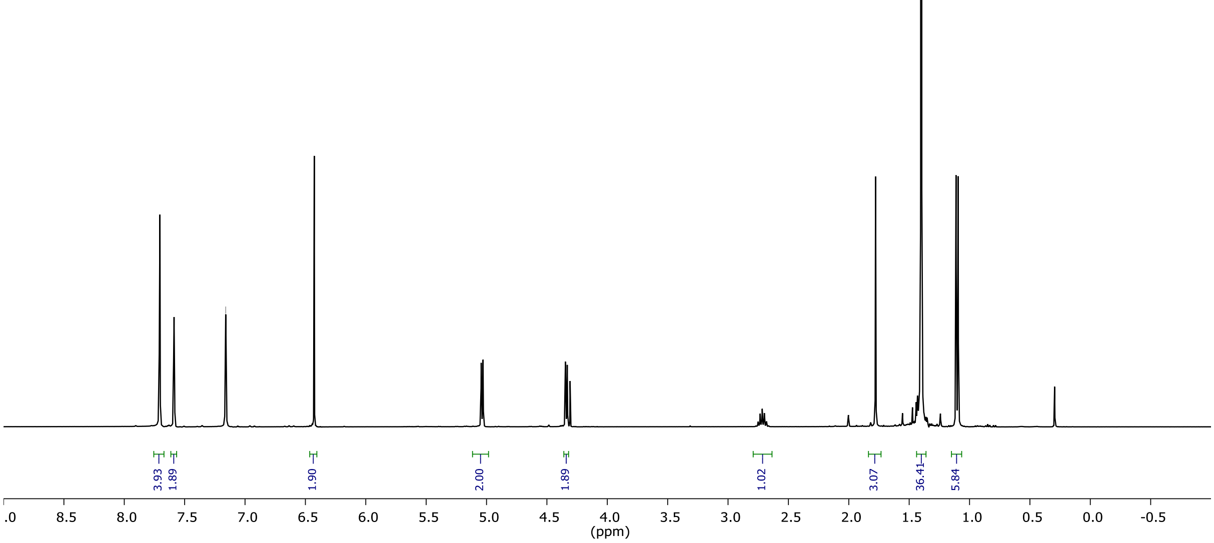
7.2 C6D6
3.93
1.89
1.90
2.00
1.89
1.02
3.07
36.41
5.84
(ppm)

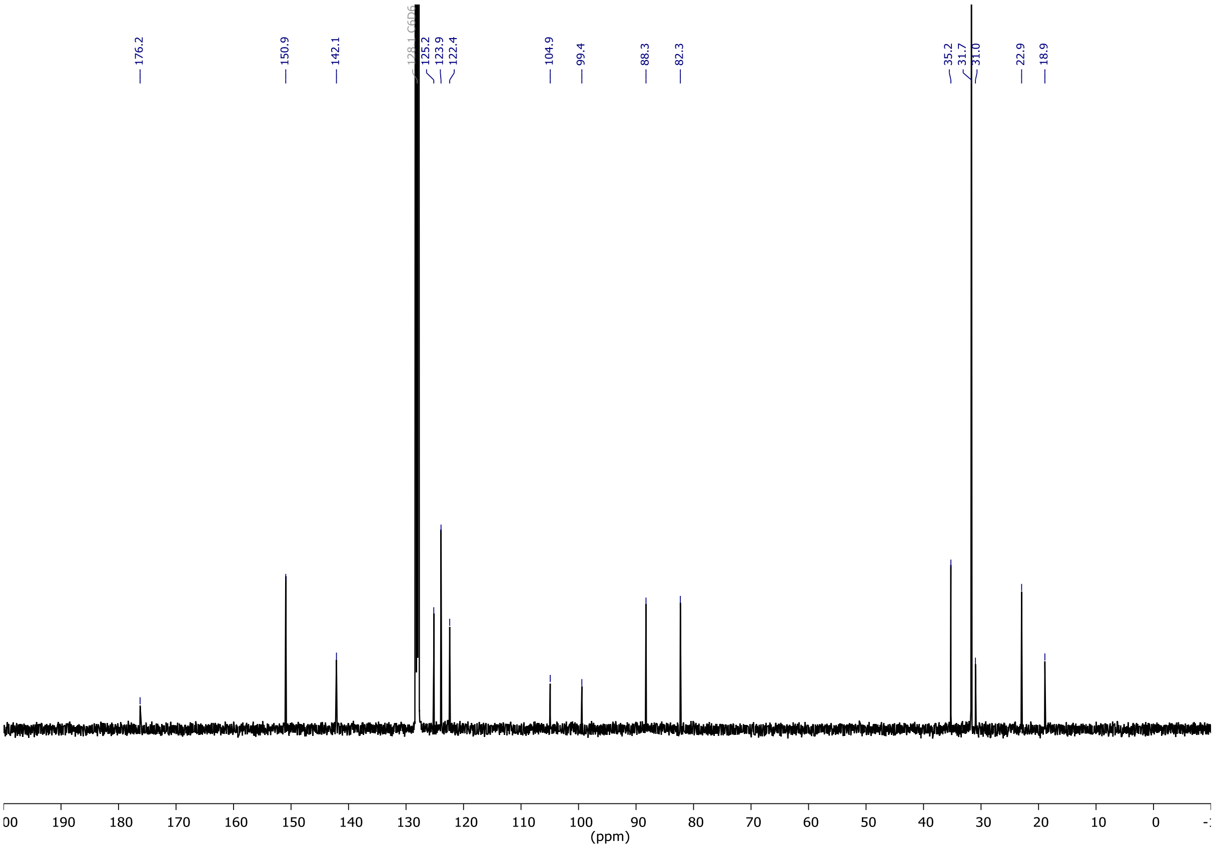
176.2
150.9
142.1
128.1 C6D6
125.2
123.9
122.4
104.9
99.4
88.3
82.3
35.2
31.7
31.0
22.9
18.9
(ppm)

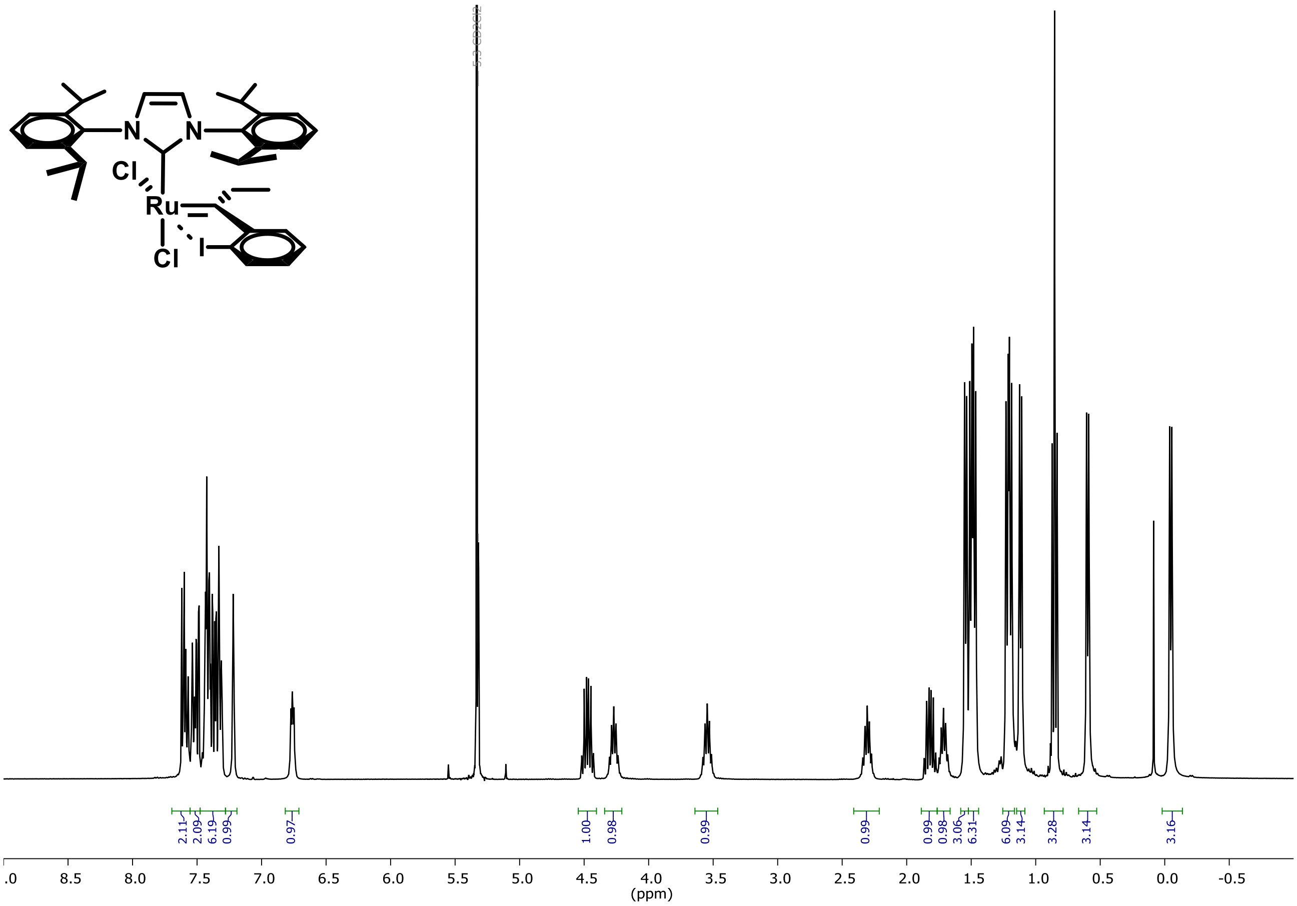
N
N
Cl
Ru
Cl
I
5.3 CD2Cl2
2.11
2.09
6.19
0.99
0.97
1.00
0.98
0.99
0.99
0.99
0.98
3.06
6.31
6.09
3.14
3.28
3.14
3.16
(ppm)

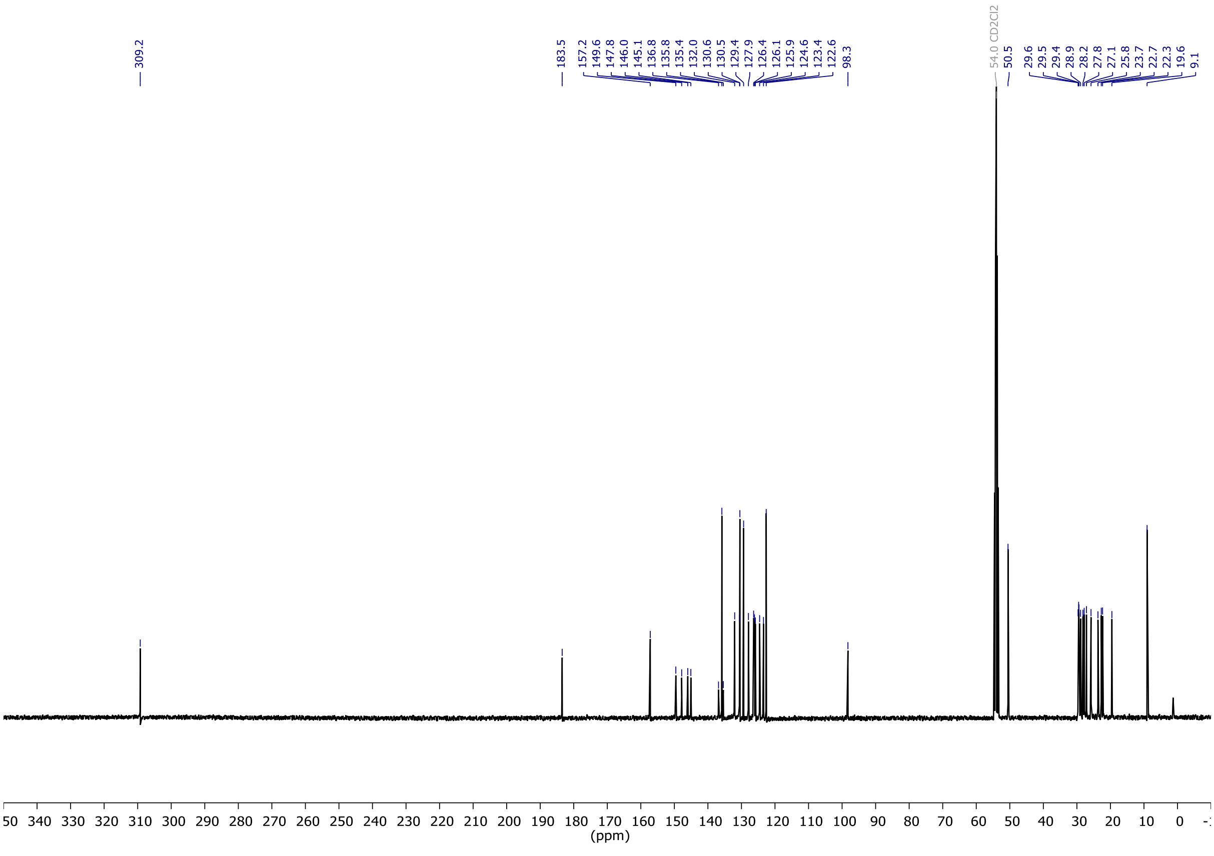
309.2
183.5
157.2
149.6
147.8
146.0
145.1
136.8
135.4
132.0
130.6
130.5
129.4
127.9
126.4
126.1
125.9
124.6
123.4
122.6
98.3
54.0 CD2Cl2
50.5
29.6
29.5
29.4
28.9
28.2
27.8
27.1
25.8
23.7
22.7
22.3
19.6
9.1
(ppm)

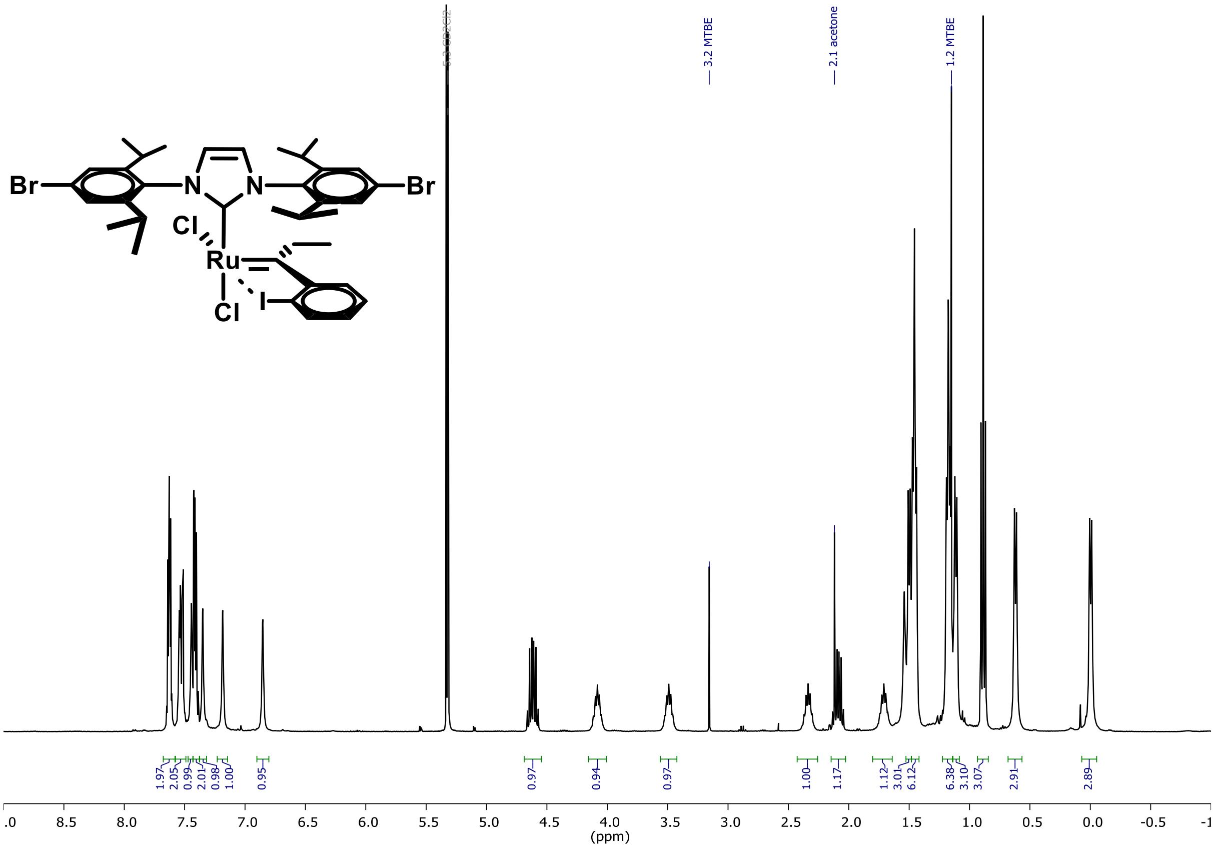
Br
N
N
Br
Cl
Ru
Cl
I
5.3 CD2Cl2
3.2 MTBE
2.1 acetone
1.2 MTBE
1.97
2.05
0.99
2.01
0.98
1.00
0.95
0.97
0.94
0.97
1.00
1.17
1.12
3.01
6.12
6.38
3.10
3.07
2.91
2.89
.0 8.5 8.0 7.5 7.0 6.5 6.0 5.5 5.0 4.5 4.0 3.5 3.0 2.5 2.0 1.5 1.0 0.5 0.0 -0.5 -1
(ppm)

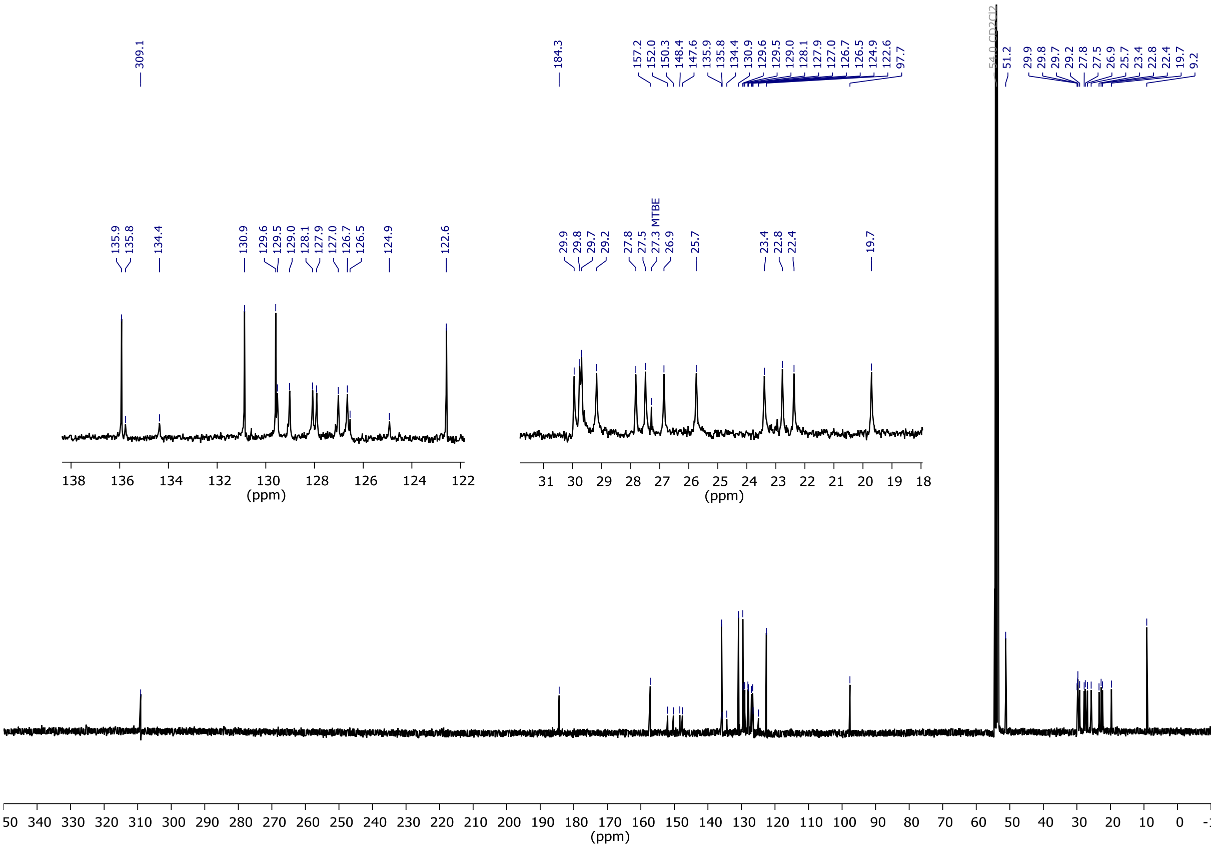
309.1
184.3
157.2
152.0
150.3
148.4
147.6
135.9
135.8
134.4
130.9
129.6
129.5
129.0
128.1
127.9
127.0
126.7
126.5
124.9
122.6
97.7
54.0 CD2Cl2
51.2
29.9
29.8
29.7
29.2
27.8
27.5
25.7
26.9
23.4
22.8
22.4
19.7
9.2
135.9
135.8
134.4
130.9
129.6
129.5
129.0
128.1
127.9
127.0
126.7
126.5
124.9
122.6
138 136 134 132 130 128 126 124 122
(ppm)
29.9
29.8
29.7
29.2
27.8
27.5
27.3 MTBE
26.9
25.7
23.4
22.8
22.4
19.7
31 30 29 28 27 26 25 24 23 22 21 20 19 18
(ppm)
50 340 330 320 310 300 290 280 270 260 250 240 230 220 210 200 190 180 170 160 150 140 130 120 110 100 90 80 70 60 50 40 30 20 10 0
(ppm)

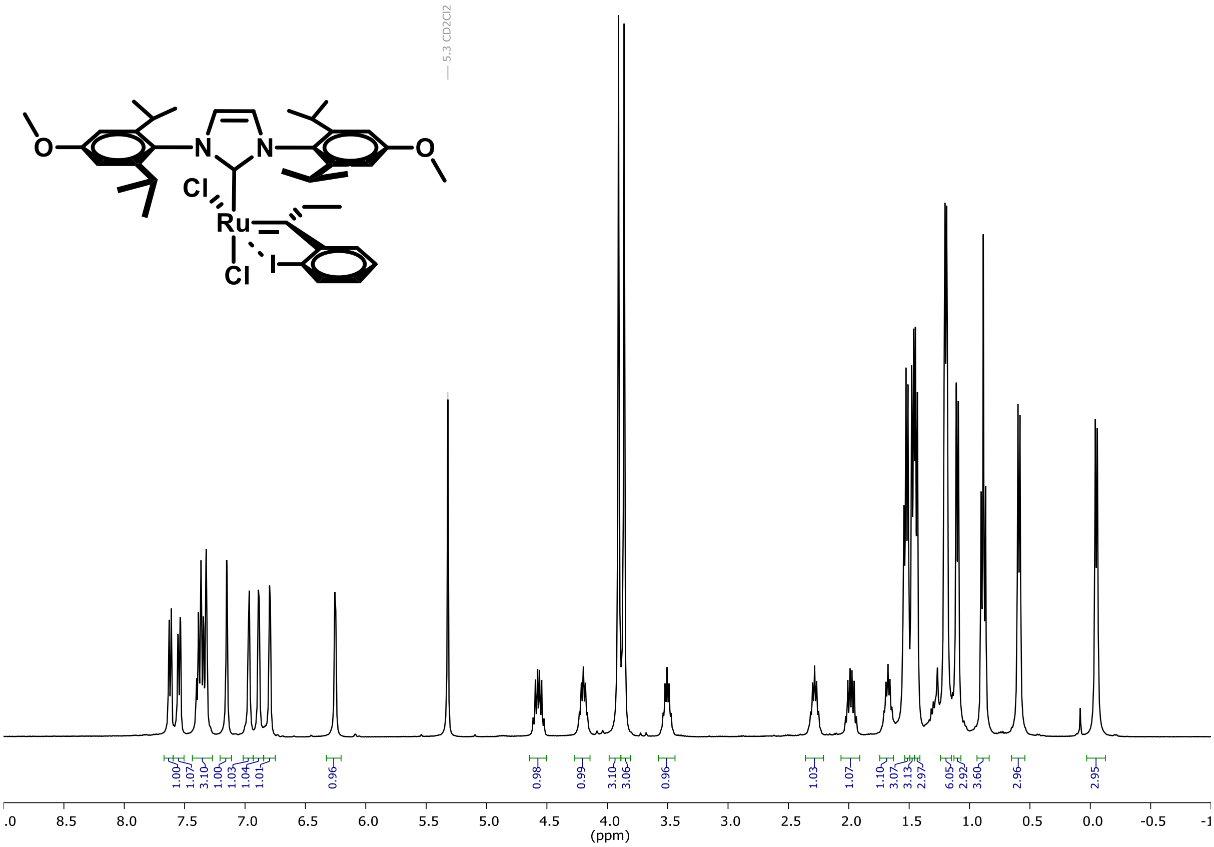

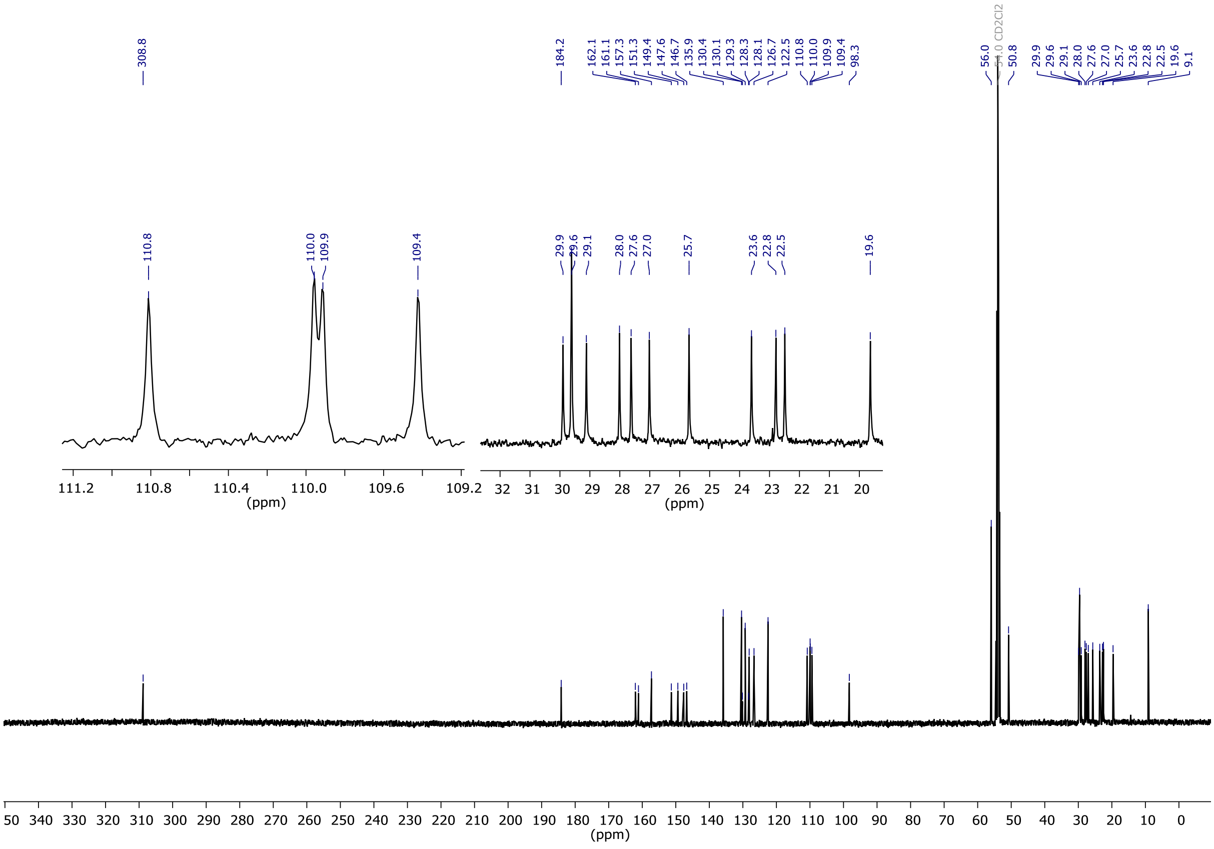

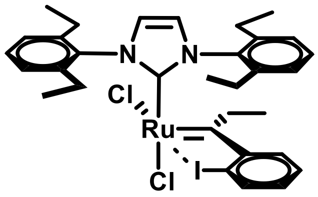

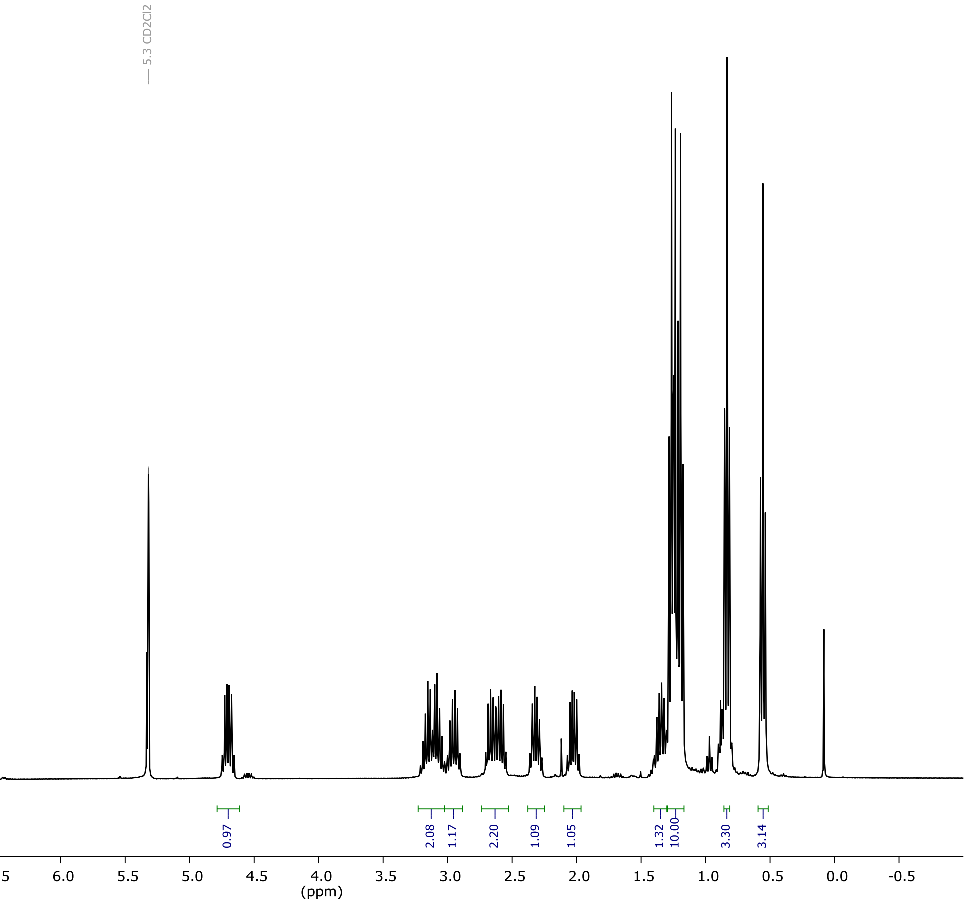
5.3 CD2Cl2
2.18
1.10
1.33
3.97
2.05
0.97
1.00
0.97
2.08
1.17
2.20
1.09
1.05
1.32
10.00
3.30
3.14
(ppm)

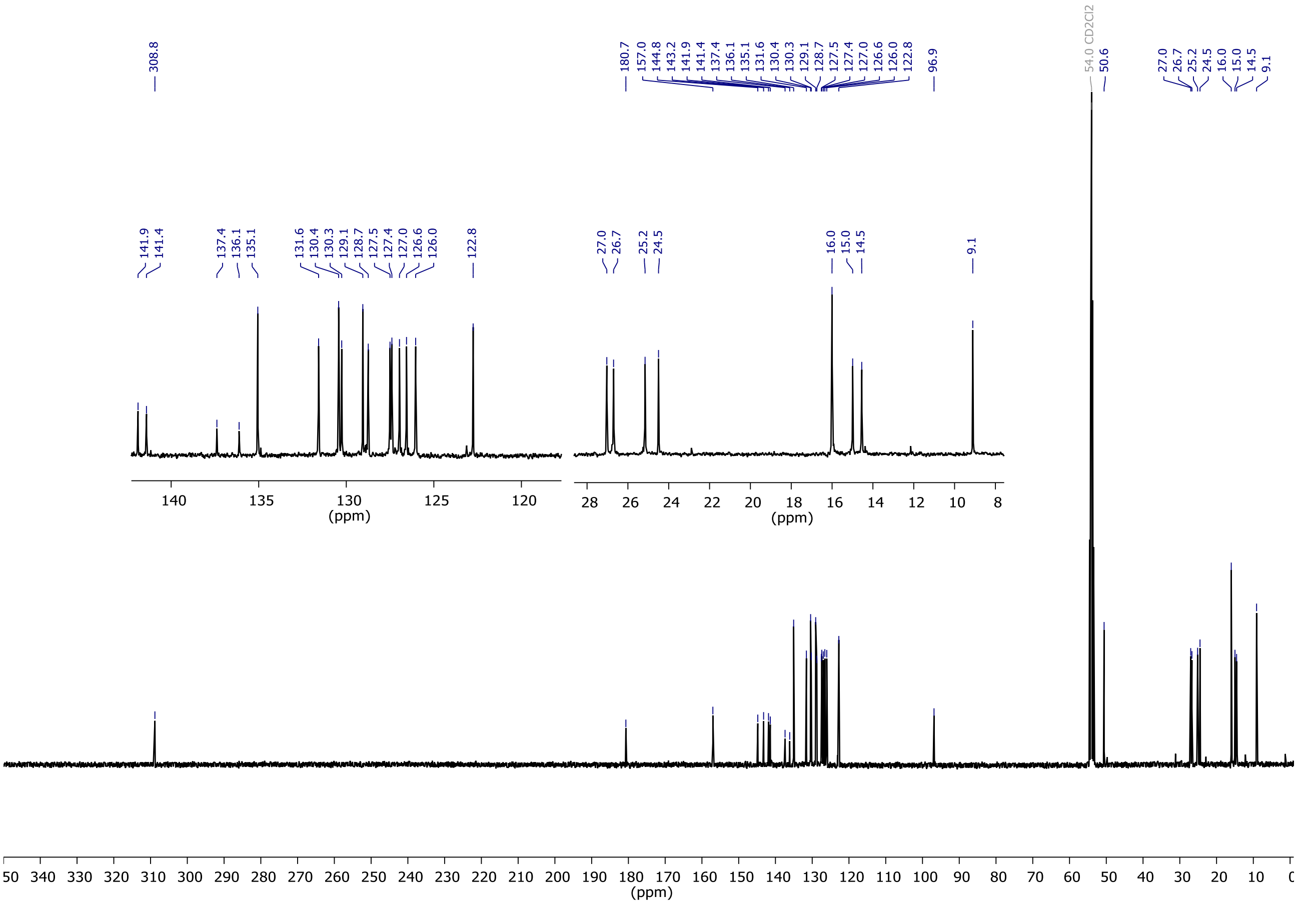
308.8
180.7
157.0
144.8
143.2
141.9
141.4
137.4
136.1
135.1
131.6
130.4
130.3
129.1
128.7
127.5
127.4
127.0
126.6
126.0
122.8
96.9
54.0 CD2Cl2
50.6
27.0
26.7
25.2
24.5
16.0
15.0
14.5
9.1
(ppm)

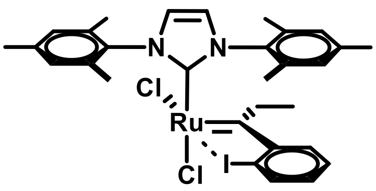
N
N
Cl
Ru
I
Cl

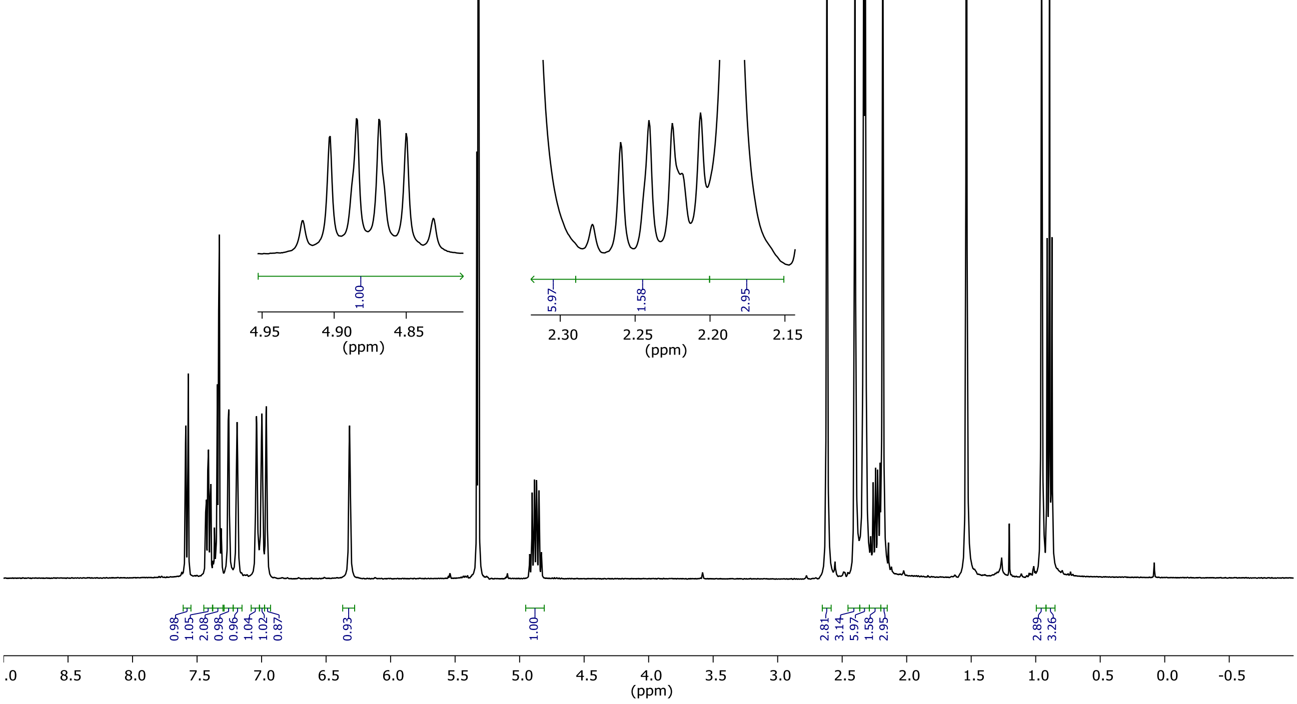
5.3 CD2Cl2
1.00
4.95 4.90 4.85
(ppm)
5.97
1.58
2.95
2.30 2.25 2.20 2.15
(ppm)
0.98 1.05 2.08 0.98 0.96 1.04 1.02 0.87
0.93
1.00
2.81 3.14 5.97 1.58 2.95
2.89 3.26
(ppm)

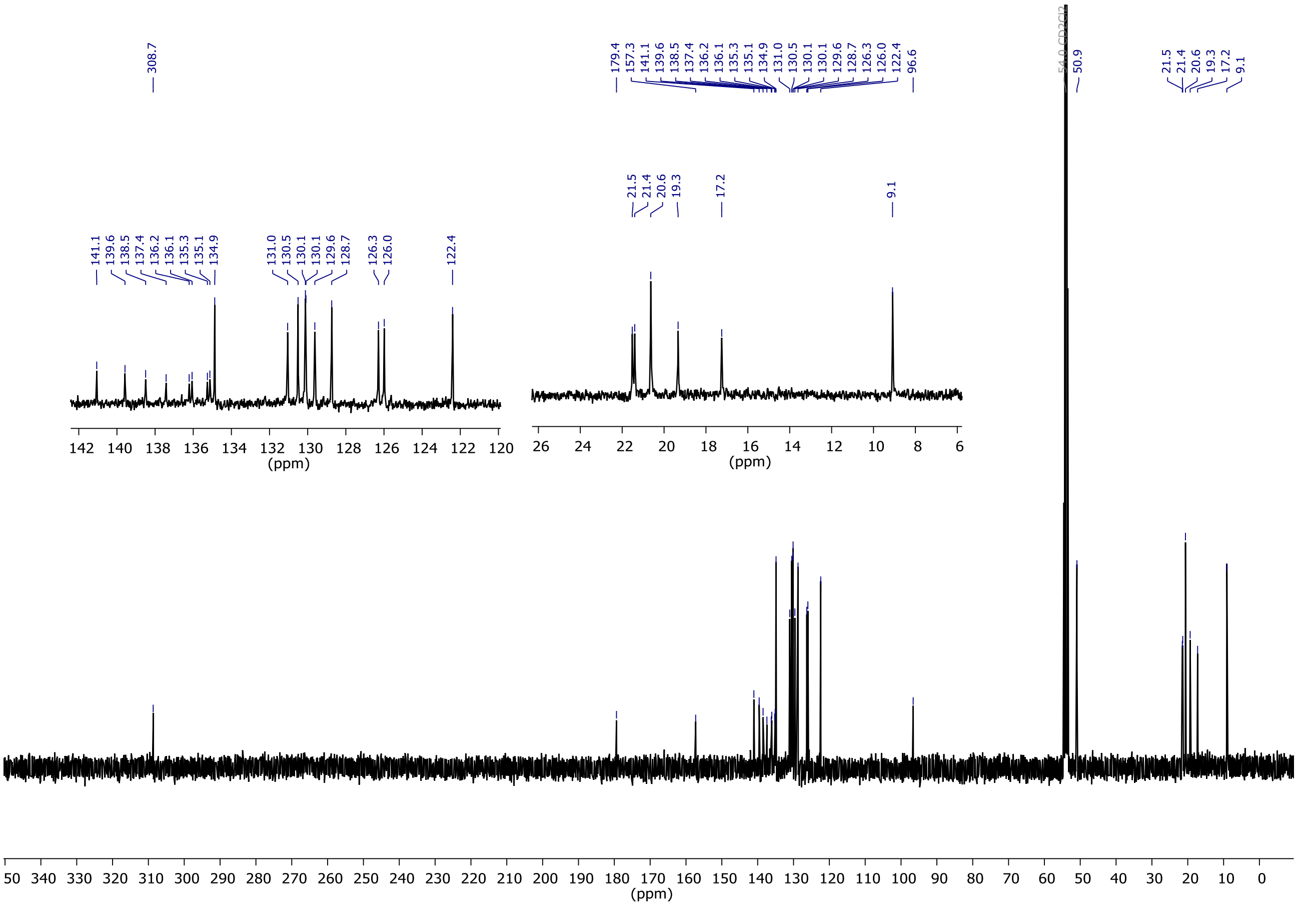
308.7
179.4 157.3 141.1 139.6 138.5 137.4 136.2 136.1 135.3 135.1 134.9 131.0 130.5 130.1 130.1 129.6 128.7 126.3 126.0 122.4 96.6
54.0 CD2Cl2
50.9
21.5 21.4 20.6 19.3 17.2 9.1
141.1 139.6 138.5 137.4 136.2 136.1 135.3 135.1 134.9 131.0 130.5 130.1 130.1 129.6 128.7 126.3 126.0 122.4
(ppm)
21.5 21.4 20.6 19.3 17.2 9.1
(ppm)
(ppm)

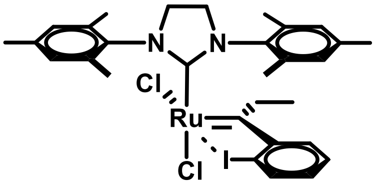

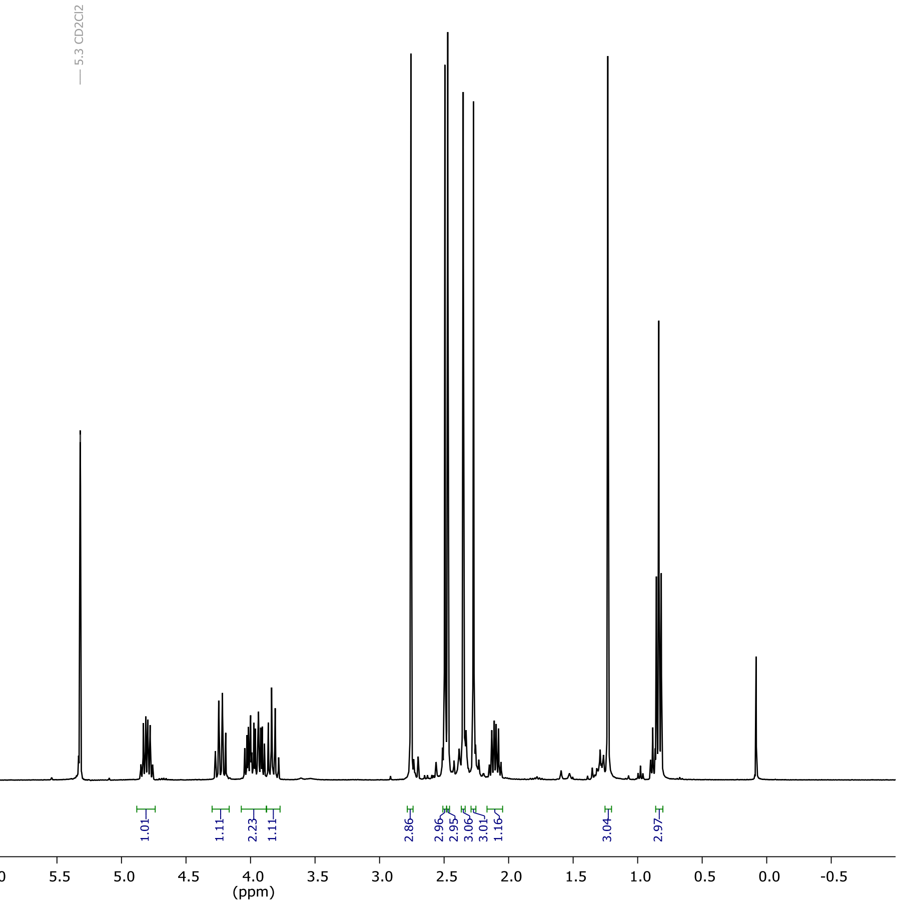

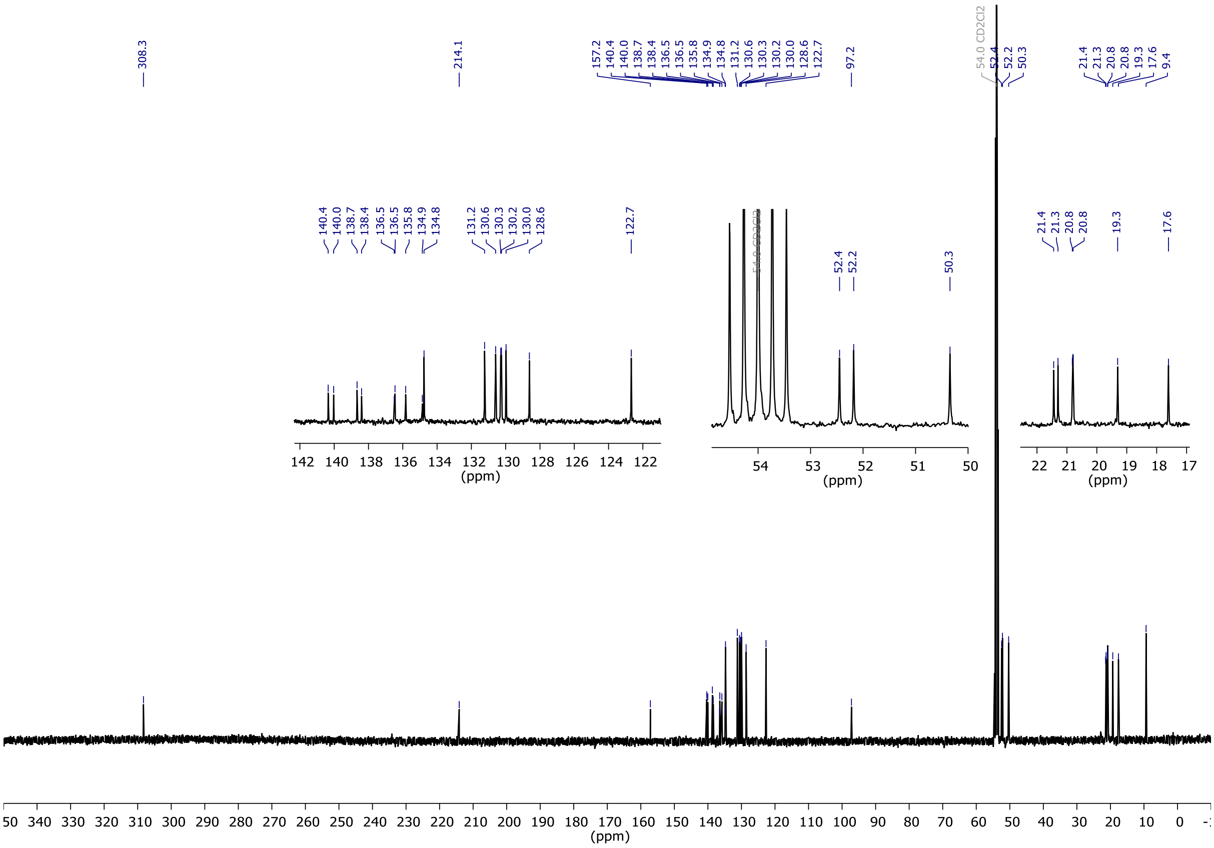

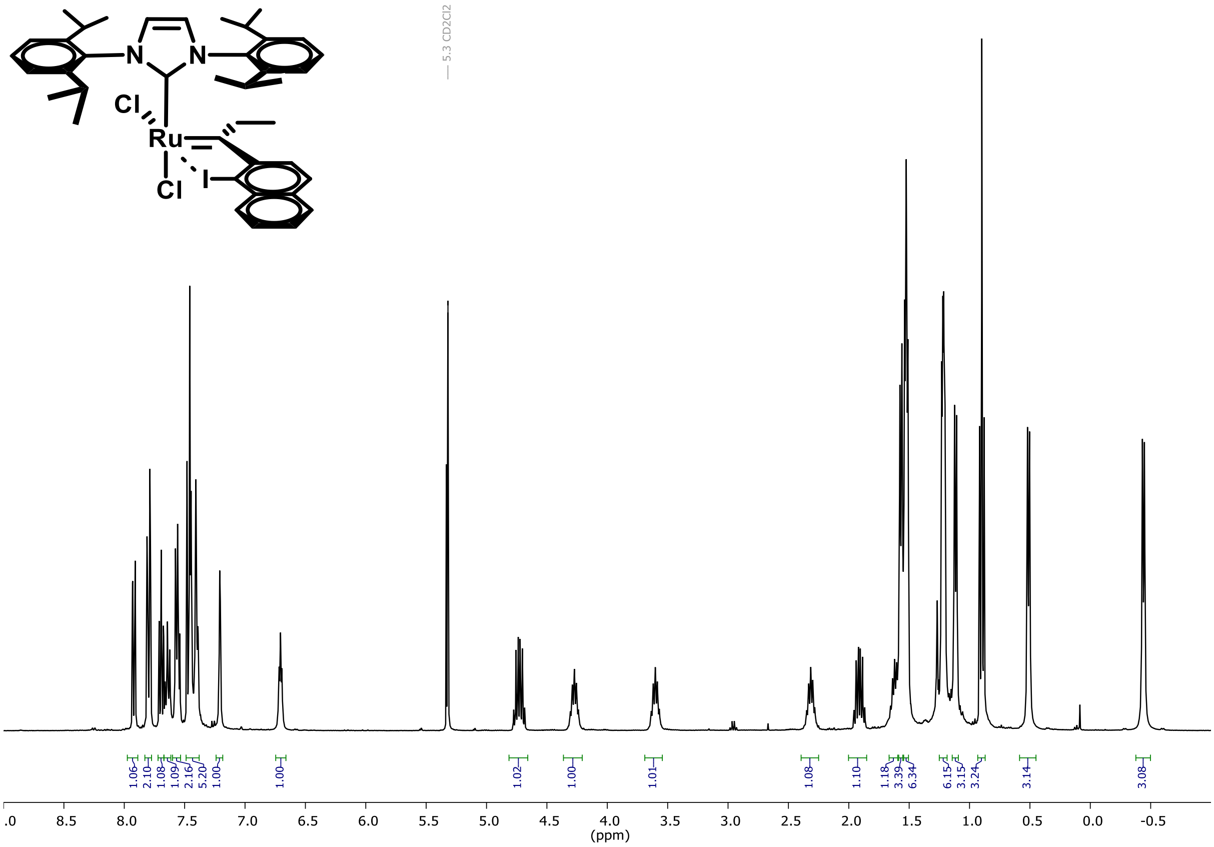
N
N
Cl
Ru
I
Cl
5.3 CD2Cl2
1.06
2.10
1.08
1.09
2.16
5.20
1.00
1.00
1.02
1.00
1.01
1.08
1.10
1.18
3.39
6.34
6.15
3.15
3.24
3.14
3.08
(ppm)

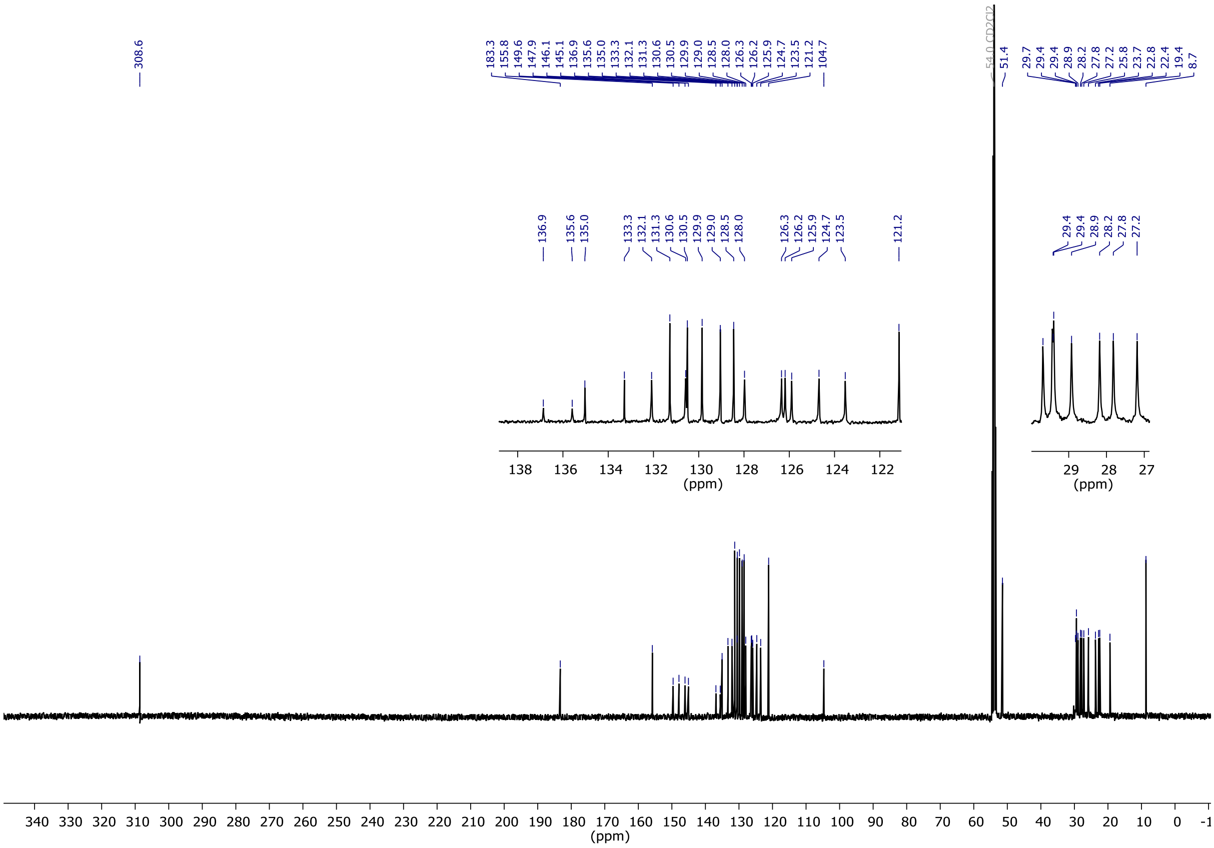
308.6
183.3
155.8
149.6
147.9
146.1
136.9
135.6
135.0
133.3
132.1
131.3
130.6
130.5
129.9
129.0
128.5
128.0
126.3
126.2
125.9
124.7
123.5
121.2
104.7
54.0 CD2Cl2
51.4
29.7
29.4
29.4
28.9
28.2
27.8
27.2
25.8
23.7
22.8
22.4
19.4
8.7
(ppm)

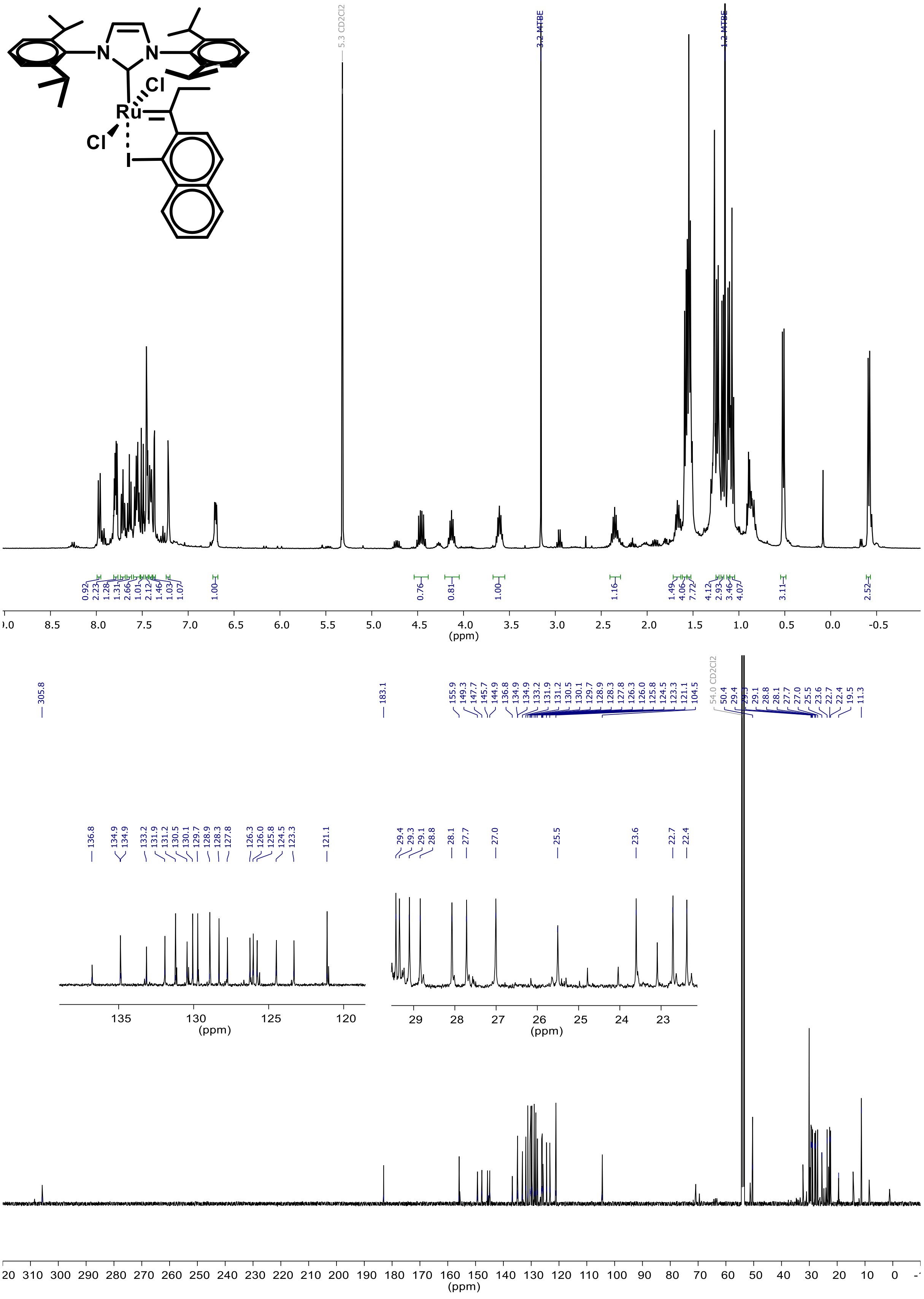

N
N
Ru
Cl
Cl
I
5.3 CD2Cl2
3.2 MTBE
1.2 MTBE
0.92 2.23 1.28 1.31 2.66 1.01 2.12 1.46 1.03 1.07
1.00
0.76
0.81
1.00
1.16
1.49 4.06 7.72
4.12 2.93 3.46 4.07
3.11
2.52
(ppm)
305.8
183.1
155.9 149.3 147.7 145.7 144.9 136.8 134.9 134.9 133.2 131.9 131.2 130.5 130.1 129.7 128.9 128.3 127.8 126.3 126.0 125.8 124.5 123.3 121.1 104.5
54.0 CD2Cl2
50.4 29.4 29.3 29.1 28.8 28.1 27.7 27.0 25.5 23.6 22.7 22.4 19.5 11.3
136.8 134.9 134.9 133.2 131.9 131.2 130.5 130.1 129.7 128.9 128.3 127.8 126.3 126.0 125.8 124.5 123.3 121.1
29.4 29.3 29.1 28.8 28.1 27.7 27.0 25.5 23.6 22.7 22.4
(ppm)
(ppm)
(ppm)

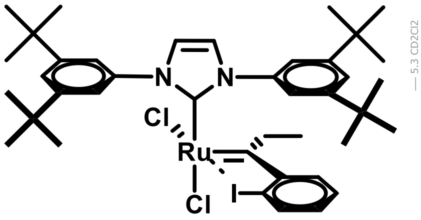

Cl
Ru
Cl
I
N
N

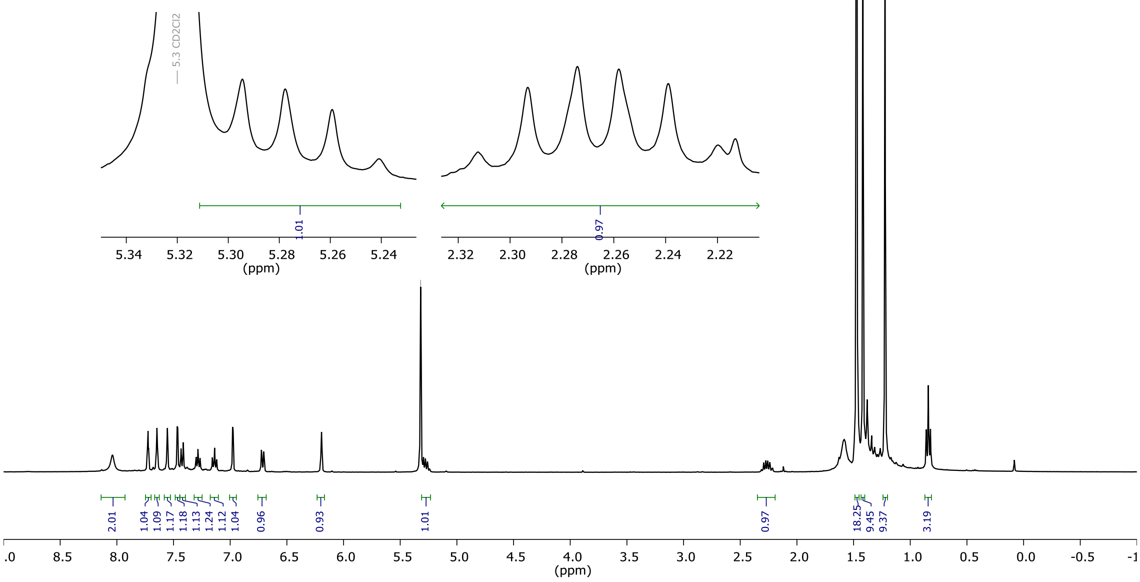

5.3 CD2Cl2
1.01
0.97
5.34 5.32 5.30 5.28 5.26 5.24
2.32 2.30 2.28 2.26 2.24 2.22
(ppm)
2.01 1.04 1.09 1.17 1.18 1.13 1.24 1.12 1.04 0.96 0.93 1.01 0.97 18.25 9.45 9.37 3.19
9.0 8.5 8.0 7.5 7.0 6.5 6.0 5.5 5.0 4.5 4.0 3.5 3.0 2.5 2.0 1.5 1.0 0.5 0.0 -0.5 -1
(ppm)

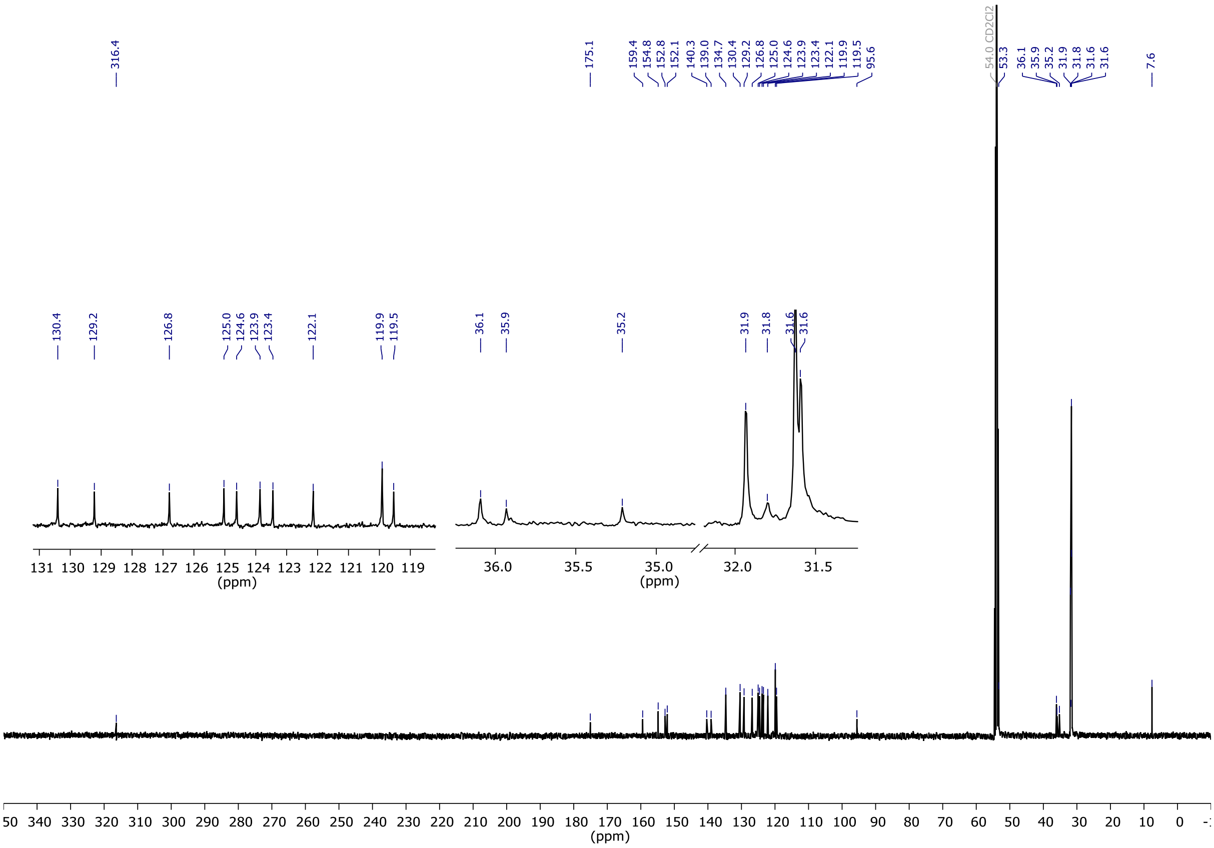

316.4
175.1
159.4 154.8 152.8 152.1 140.3 139.0 134.7 130.4 129.2 126.8 125.0 124.6 123.9 123.4 122.1 119.9 119.5 95.6
54.0 CD2Cl2
53.3
36.1 35.9 35.2 31.9 31.8 31.6 31.6
7.6
131 130 129 128 127 126 125 124 123 122 121 120 119
36.0 35.5 35.0 32.0 31.5
(ppm)
350 340 330 320 310 300 290 280 270 260 250 240 230 220 210 200 190 180 170 160 150 140 130 120 110 100 90 80 70 60 50 40 30 20 10 0
(ppm)

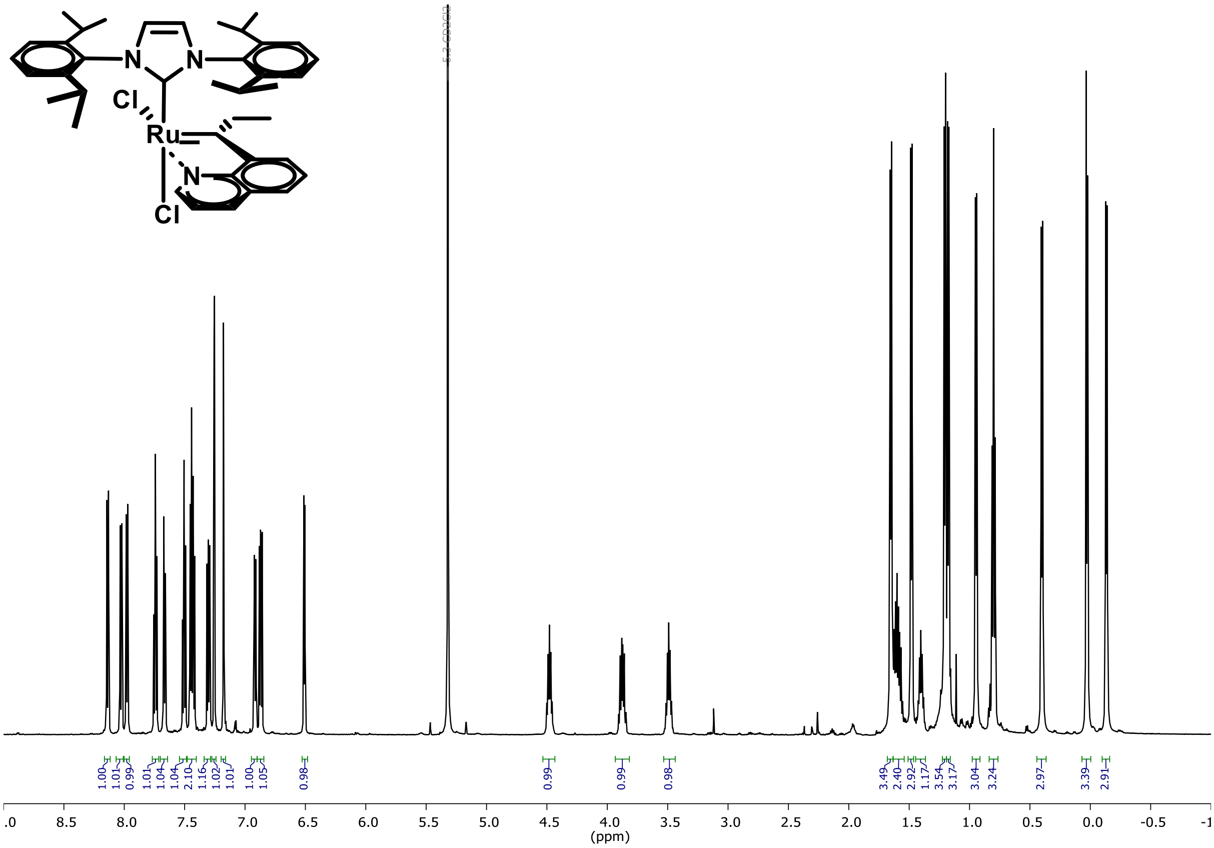

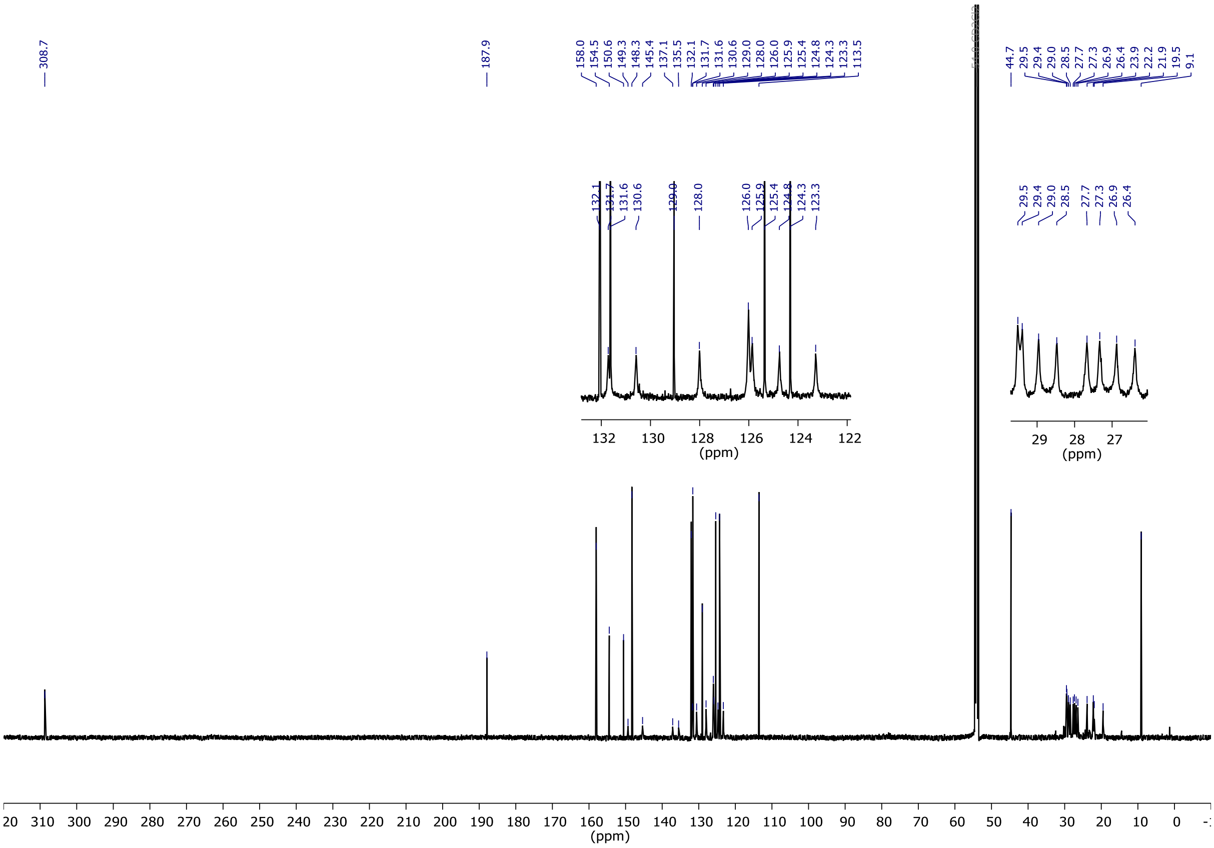

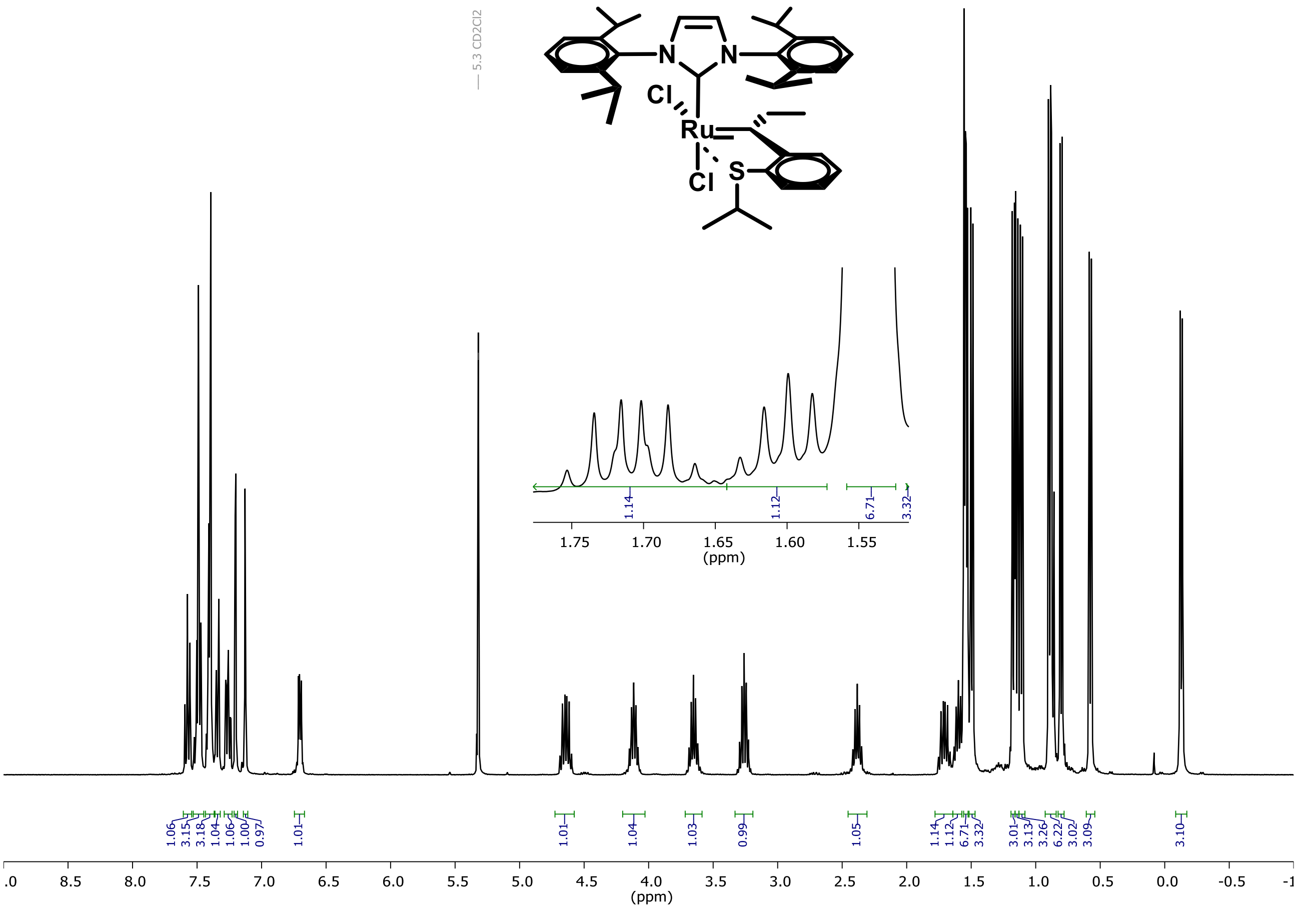
5.3 CD2Cl2
N
N
Cl
Ru
Cl
S
1.14
1.12
6.71
3.32
1.75 1.70 1.65 1.60 1.55
(ppm)
1.06
3.15
3.18
1.04
1.06
1.00
0.97
1.01
1.01
1.04
1.03
0.99
1.05
1.14
1.12
6.71
3.32
3.01
3.13
3.26
6.22
3.02
3.09
3.10
9.0 8.5 8.0 7.5 7.0 6.5 6.0 5.5 5.0 4.5 4.0 3.5 3.0 2.5 2.0 1.5 1.0 0.5 0.0 -0.5 -1
(ppm)

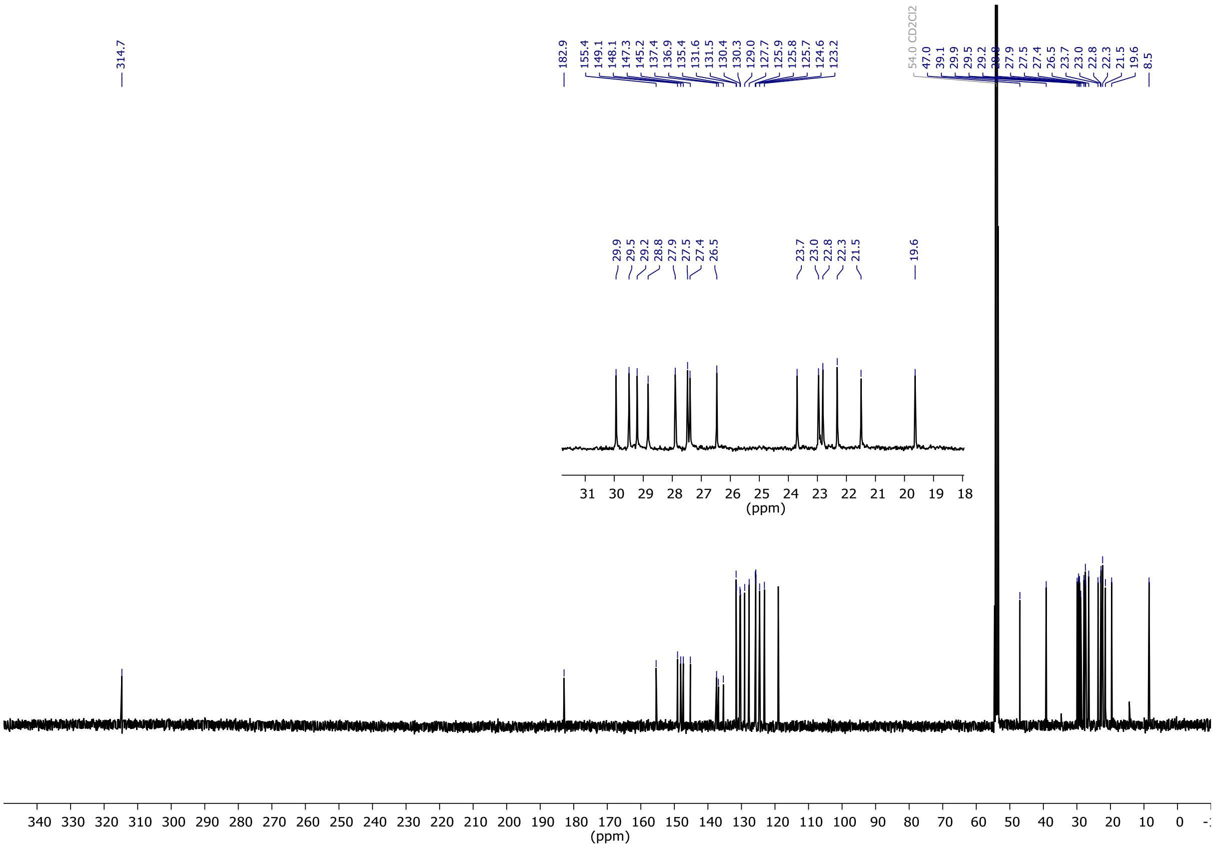
314.7
182.9
155.4
149.1
148.1
147.3
145.2
137.4
136.9
135.4
131.6
131.5
130.4
130.3
129.0
127.7
125.9
125.8
125.7
124.6
123.2
54.0 CD2Cl2
47.0
39.1
29.9
29.5
29.2
28.8
27.9
27.5
27.4
26.5
23.7
23.0
22.8
22.3
21.5
19.6
8.5
31 30 29 28 27 26 25 24 23 22 21 20 19 18
(ppm)
340 330 320 310 300 290 280 270 260 250 240 230 220 210 200 190 180 170 160 150 140 130 120 110 100 90 80 70 60 50 40 30 20 10 0
(ppm)

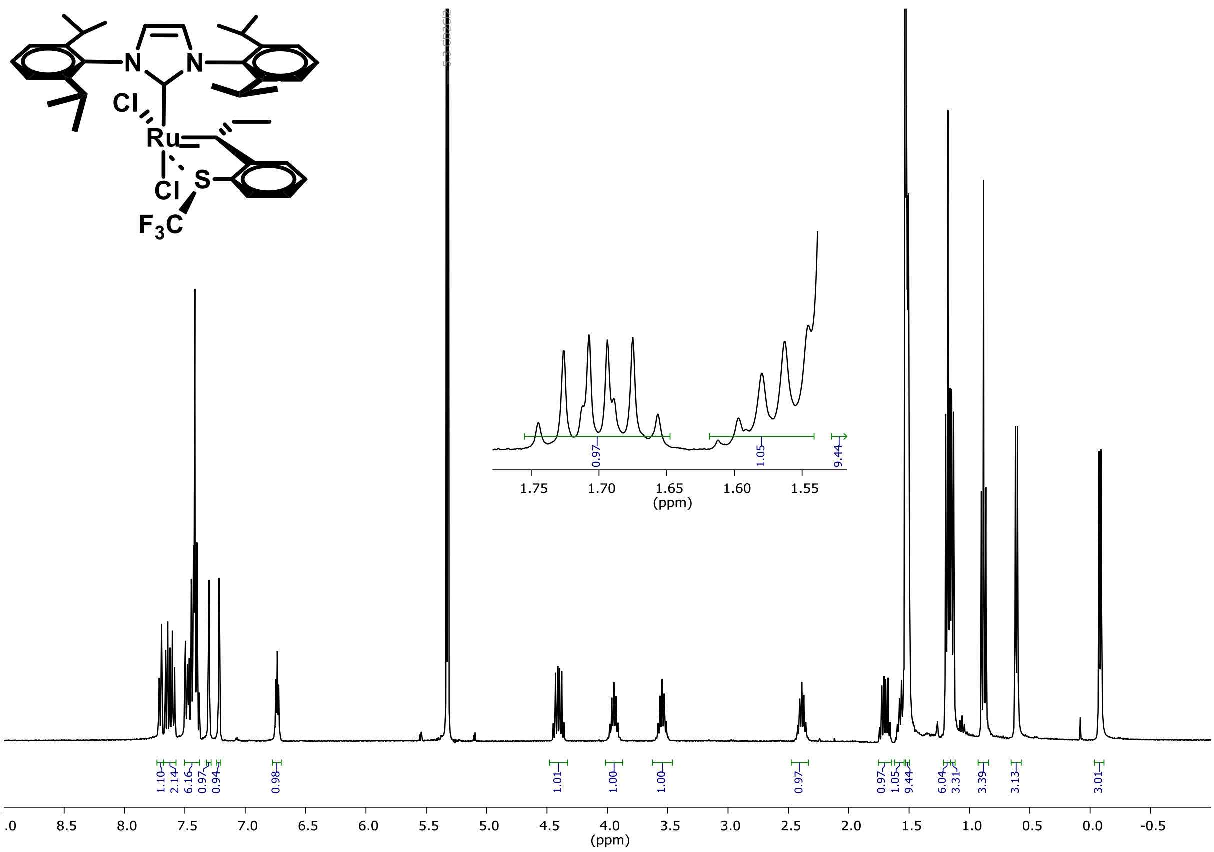
N
N
Cl
Ru
S
Cl
F3C
(ppm)

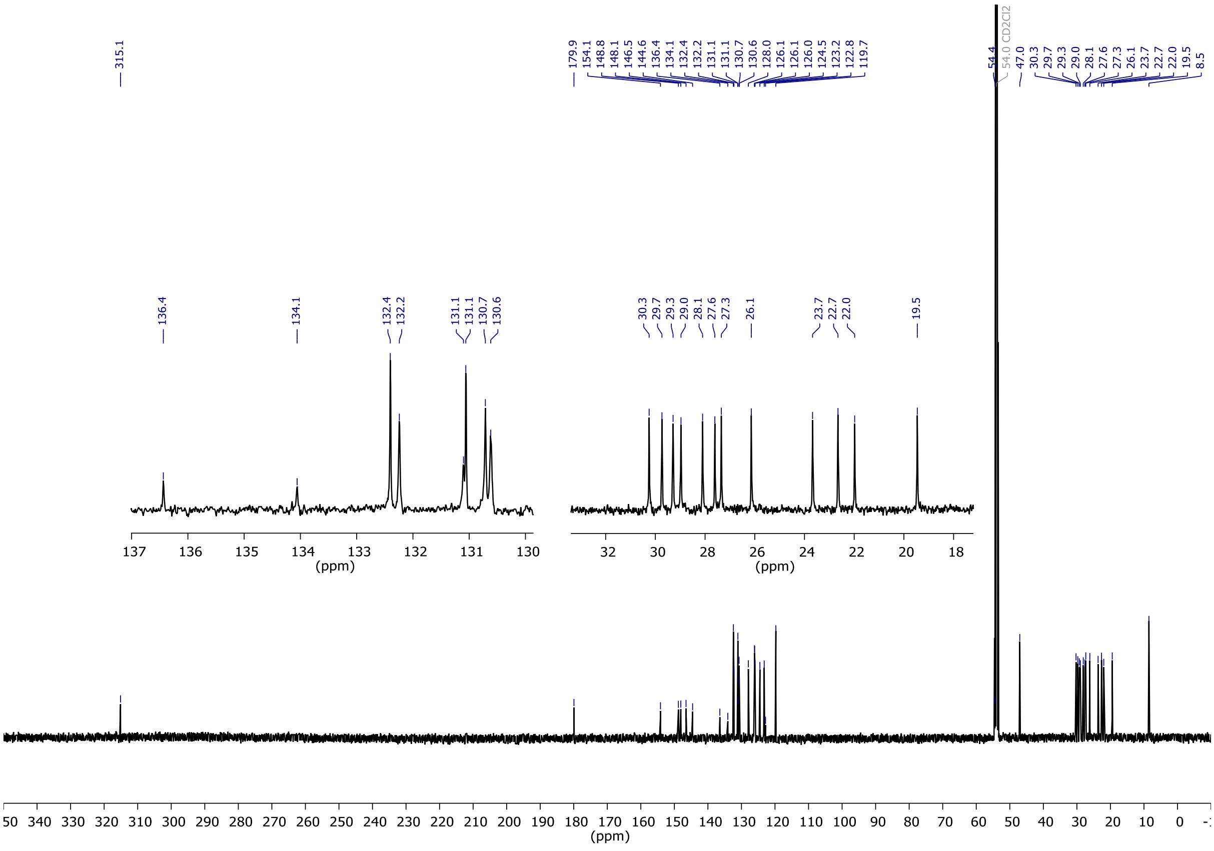
(ppm)

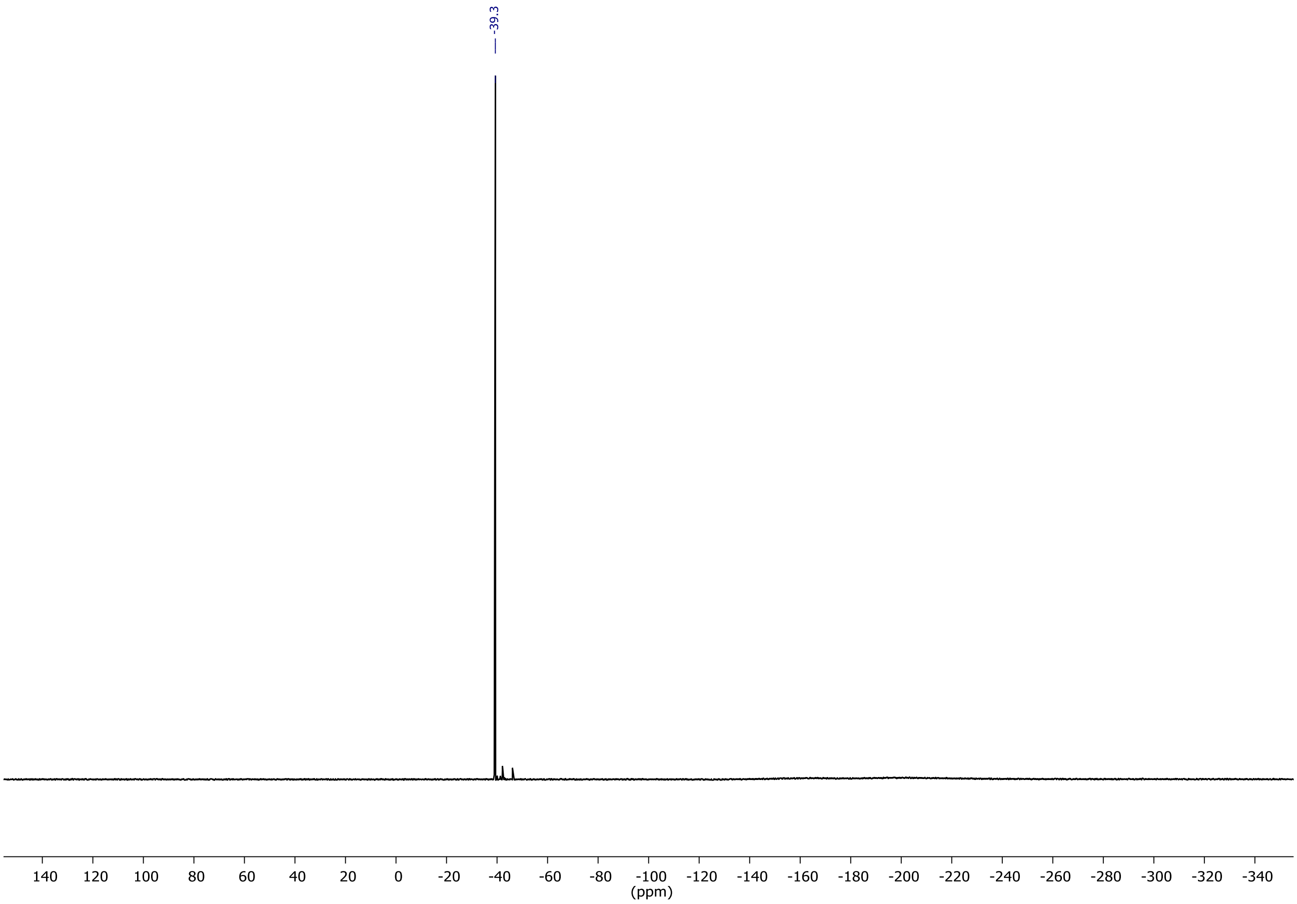
-39.3
140 120 100 80 60 40 20 0 -20 -40 -60 -80 -100 -120 -140 -160 -180 -200 -220 -240 -260 -280 -300 -320 -340
(ppm)

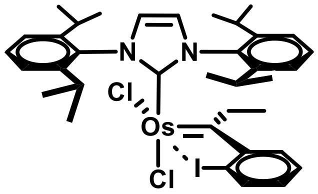
N
N
Cl
Os
Cl
I

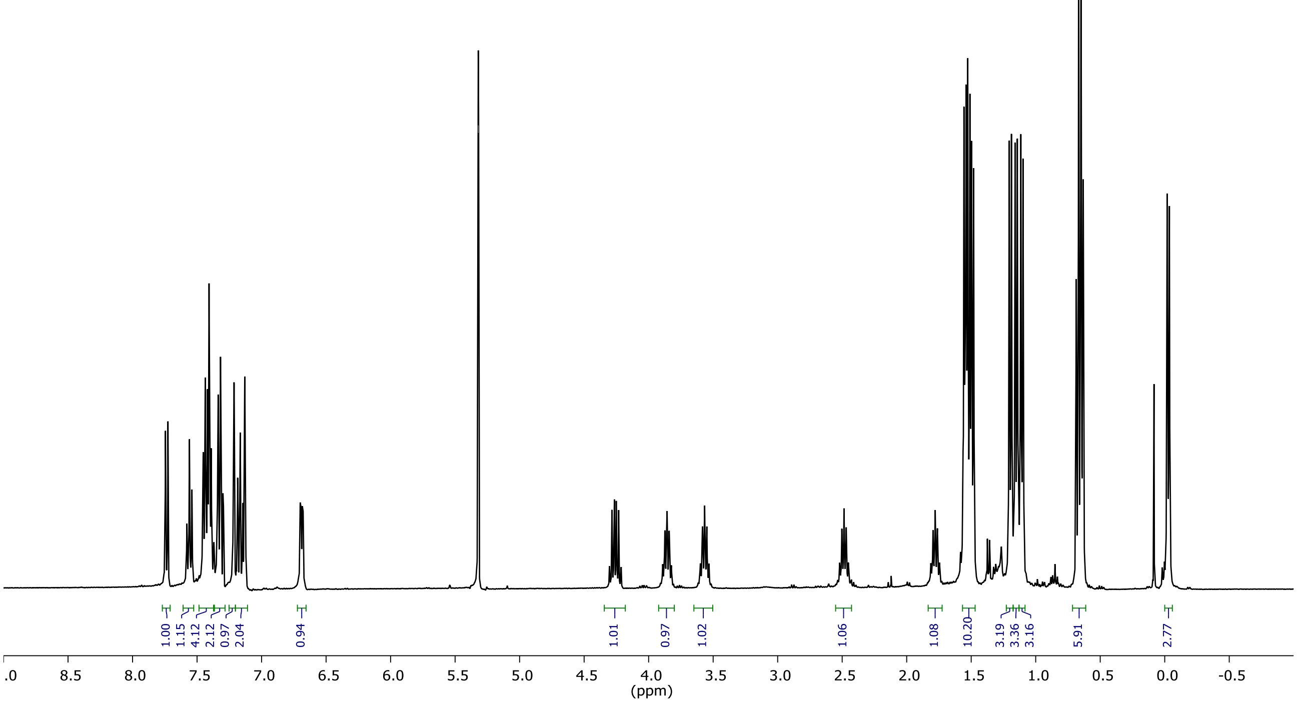
5.3 CD2Cl2
1.00
1.15
4.12
2.12
0.97
2.04
0.94
1.01
0.97
1.02
1.06
1.08
10.20
3.19
3.36
3.16
5.91
2.77
(ppm)

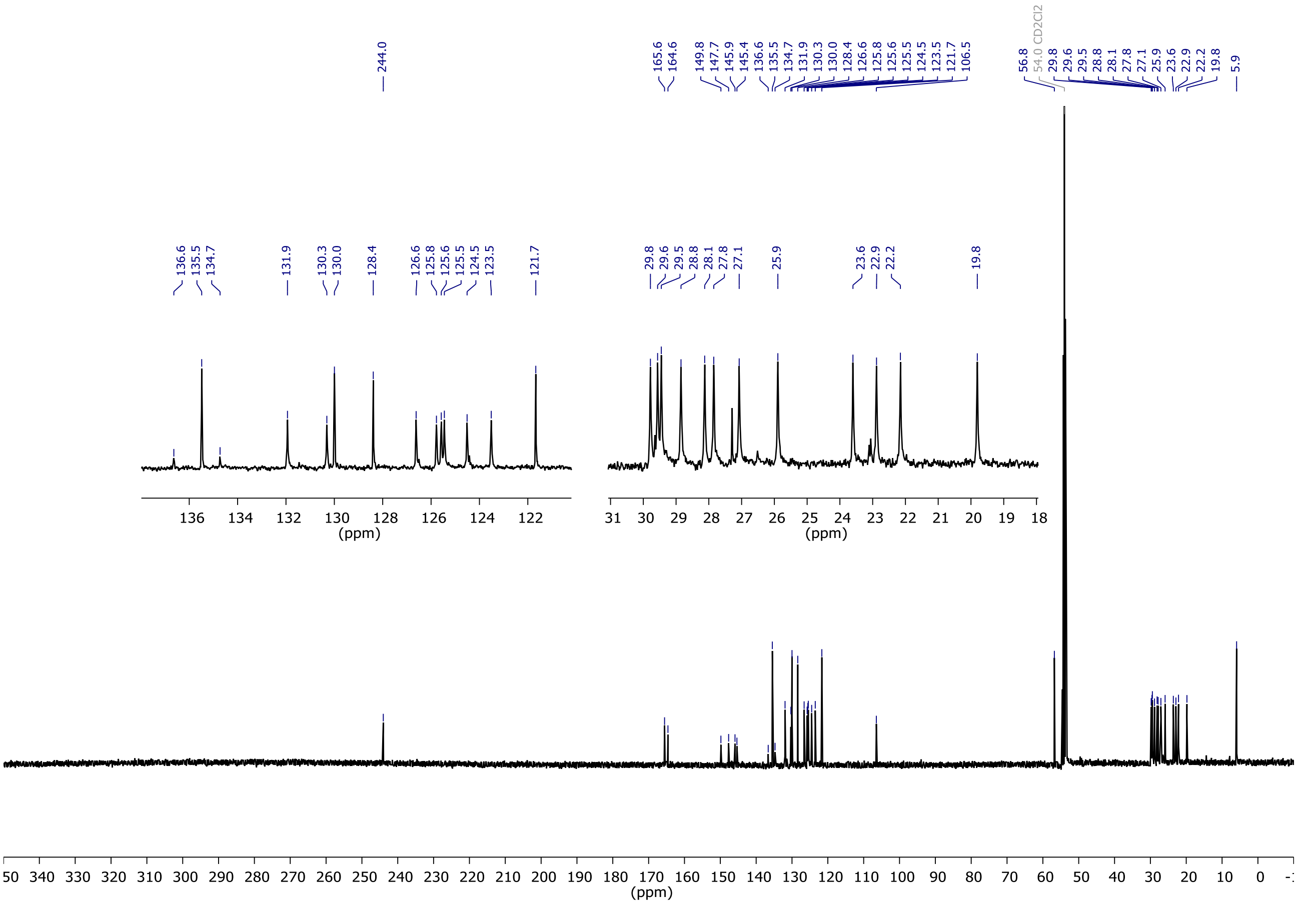
244.0
165.6
164.6
149.8
147.7
145.9
145.4
136.6
135.5
134.7
131.9
130.3
130.0
128.4
126.6
125.8
125.6
125.5
124.5
123.5
121.7
106.5
56.8
54.0 CD2Cl2
29.8
29.6
29.5
28.8
28.1
27.8
27.1
25.9
23.6
22.9
22.2
19.8
5.9
(ppm)

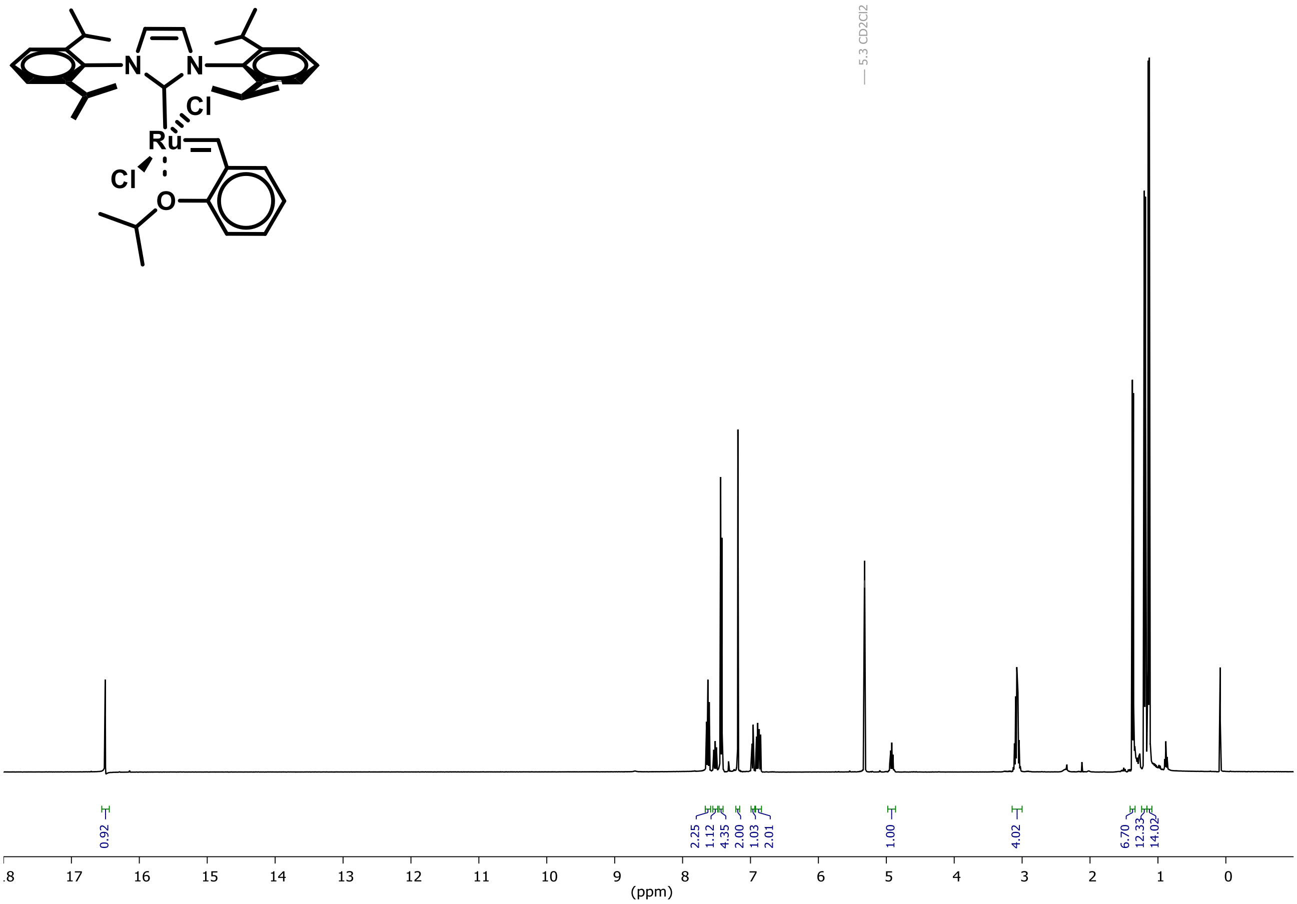
N
N
Cl
Ru
Cl
O
5.3 CD2Cl2
0.92
2.25
1.12
4.35
2.00
1.03
2.01
1.00
4.02
6.70
12.33
14.02
(ppm)

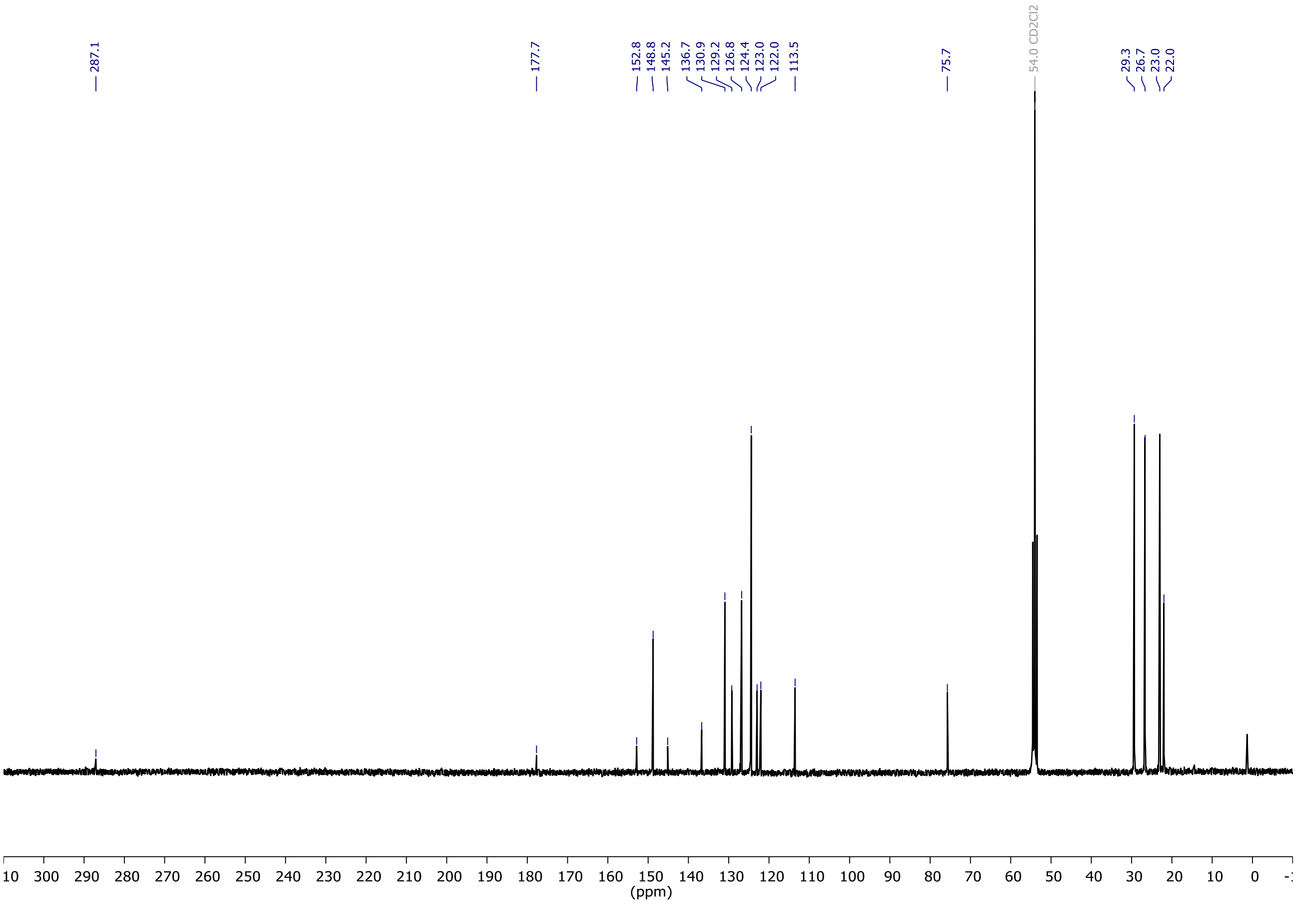
287.1
177.7
152.8
148.8
145.2
136.7
130.9
129.2
126.8
124.4
123.0
122.0
113.5
75.7
54.0 CD2Cl2
29.3
26.7
23.0
22.0
(ppm)

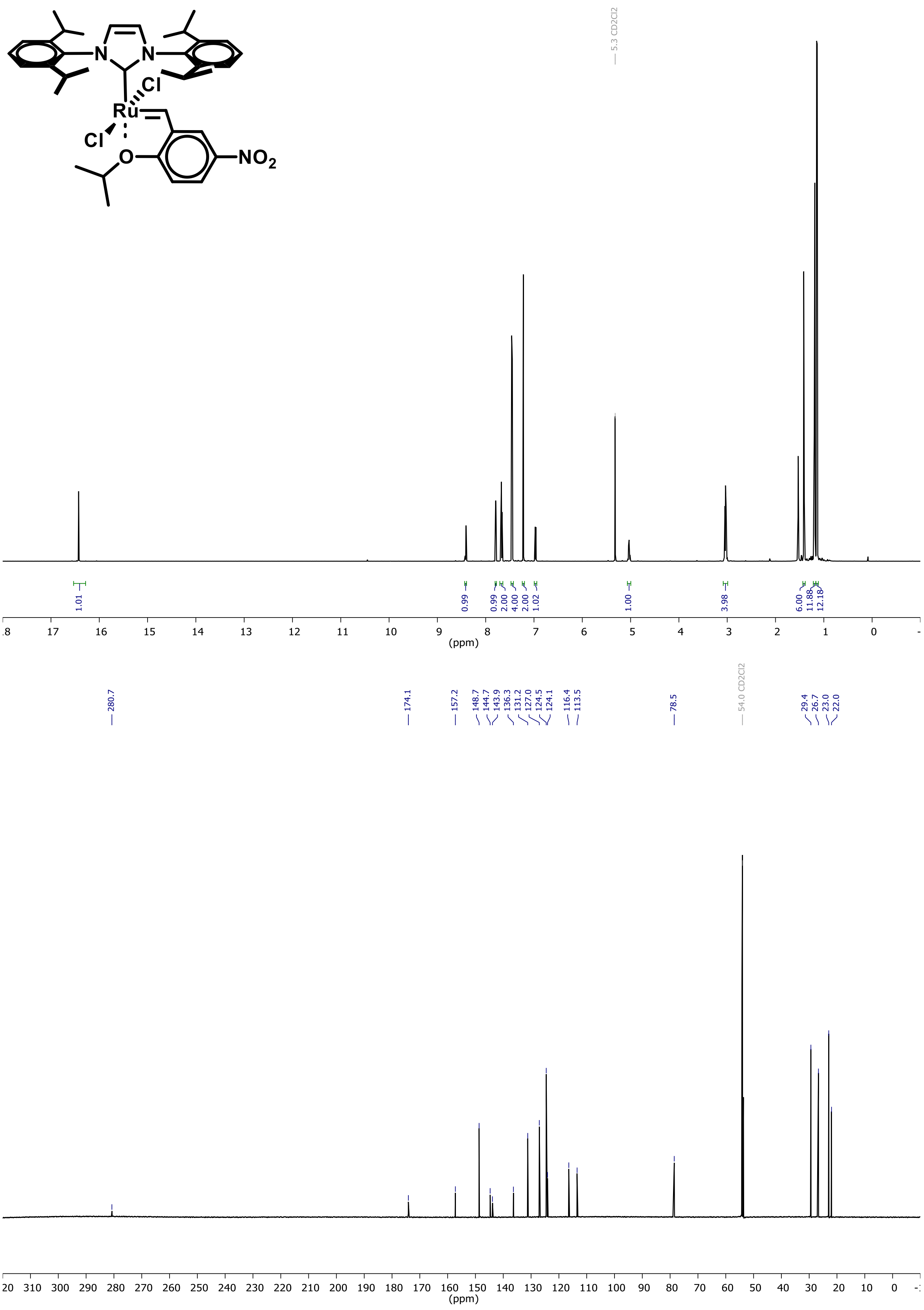

N
N
Cl
Ru
Cl
O
NO2
5.3 CD2Cl2
1.01
0.99
0.99
2.00
4.00
2.00
1.02
1.00
3.98
6.00
11.88
12.18
(ppm)
280.7
174.1
157.2
148.7
144.7
143.9
136.3
131.2
127.0
124.5
124.1
116.4
113.5
78.5
54.0 CD2Cl2
29.4
26.7
23.0
22.0
(ppm)

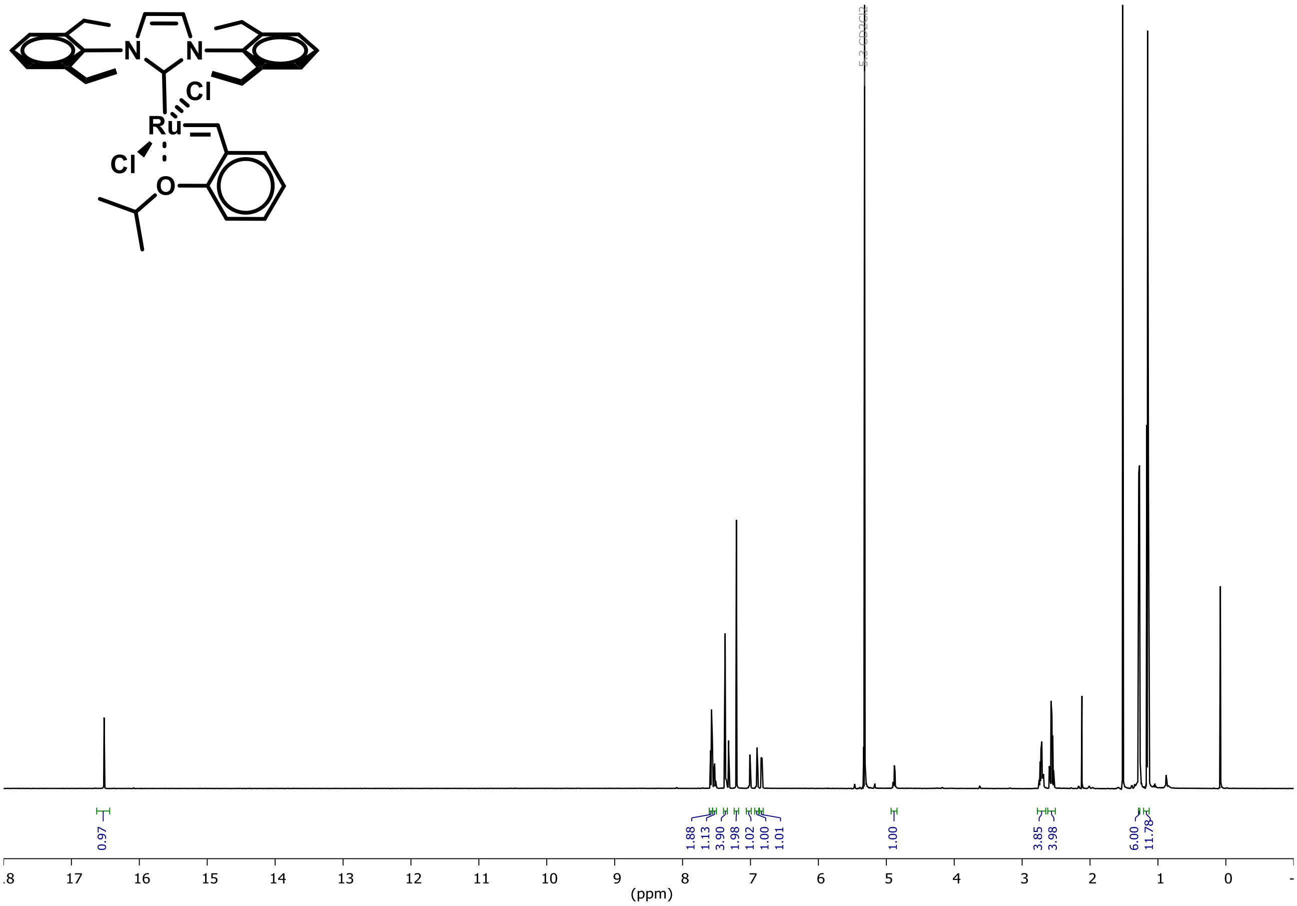
N
N
Cl
Ru
Cl
O
5.3 CD2Cl2
0.97
1.88
1.13
3.90
1.98
1.02
1.00
1.01
1.00
3.85
3.98
6.00
11.78
17
16
15
14
13
12
11
10
9
8
7
6
5
4
3
2
1
0
(ppm)

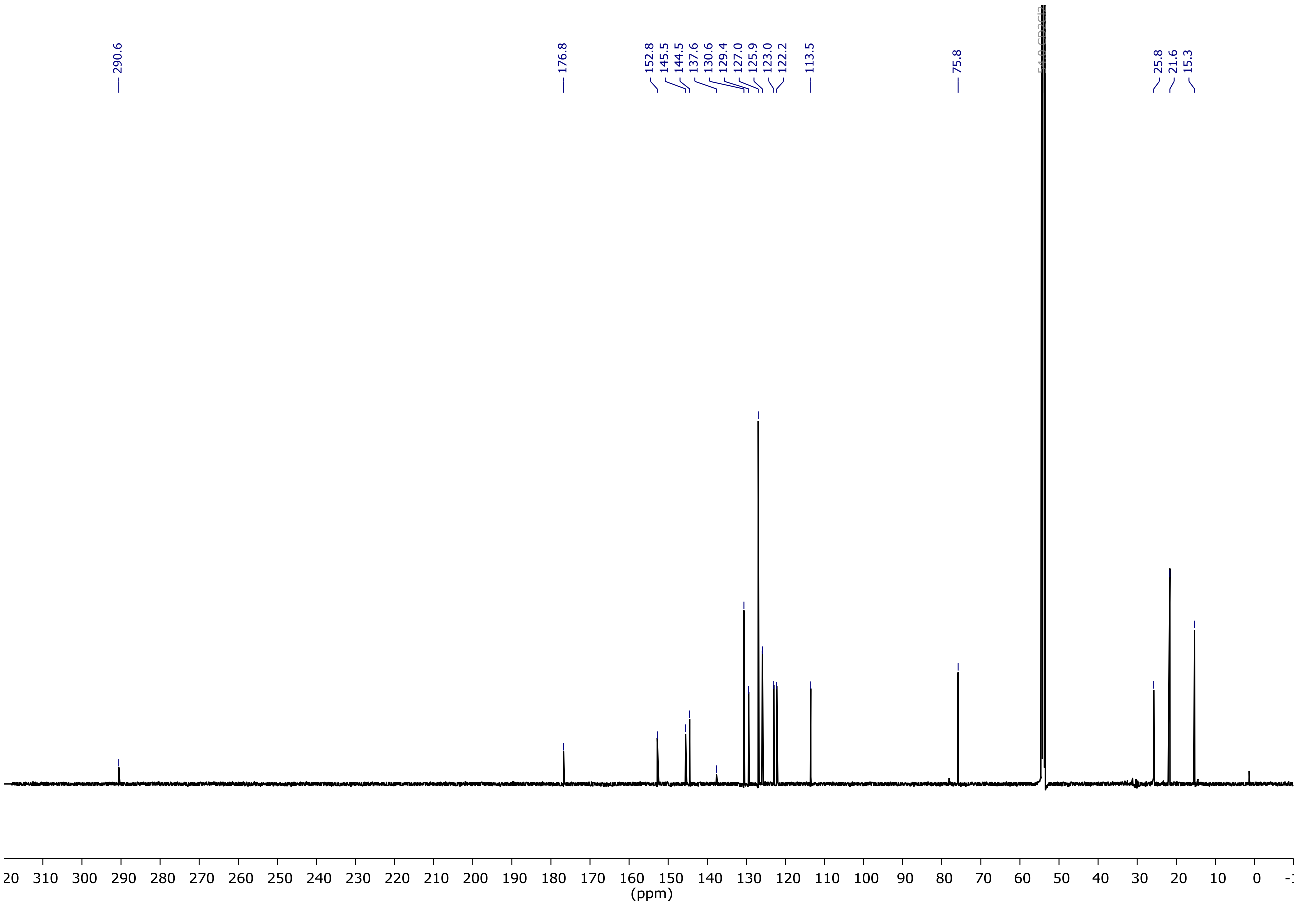
290.6
176.8
152.8
145.5
144.5
137.6
130.6
129.4
127.0
125.9
123.0
122.2
113.5
75.8
25.8
21.6
15.3
310
300
290
280
270
260
250
240
230
220
210
200
190
180
170
160
150
140
130
120
110
100
90
80
70
60
50
40
30
20
10
0
(ppm)

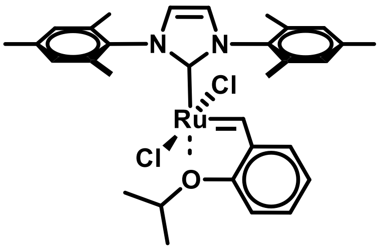
N
N
Ru
Cl
Cl
O

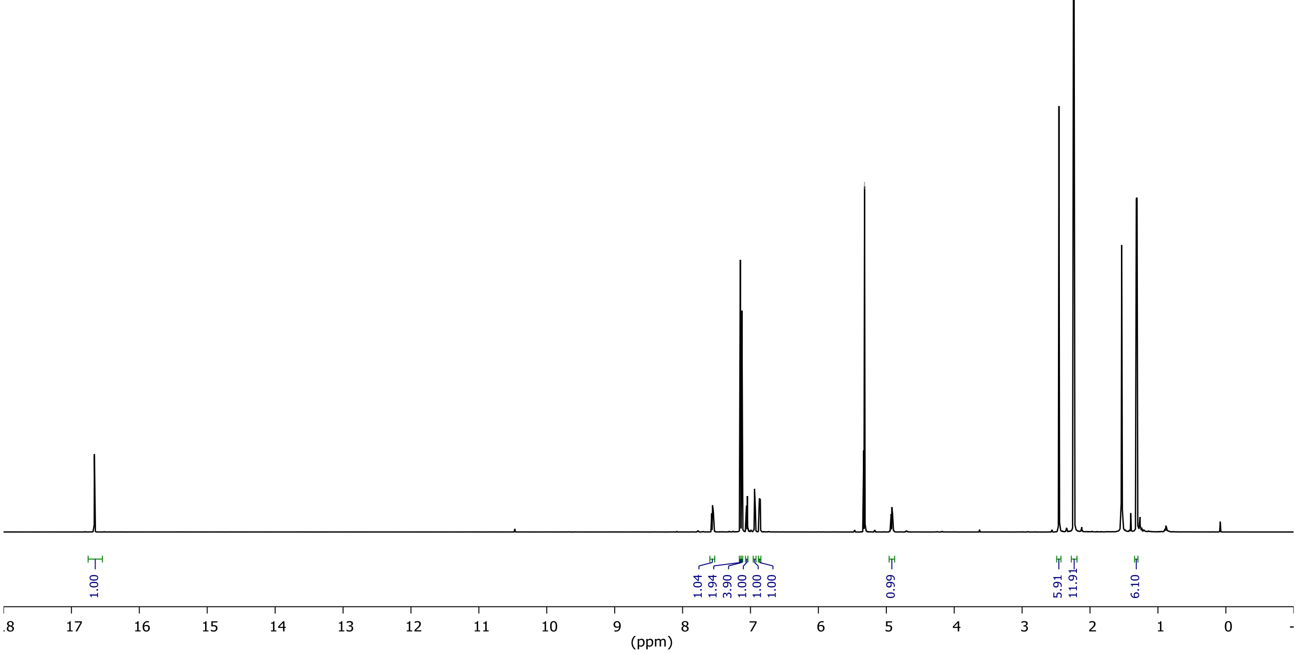
5.3 CD2Cl2
1.00
1.04
1.94
3.90
1.00
1.00
1.00
0.99
5.91
11.91
6.10
(ppm)

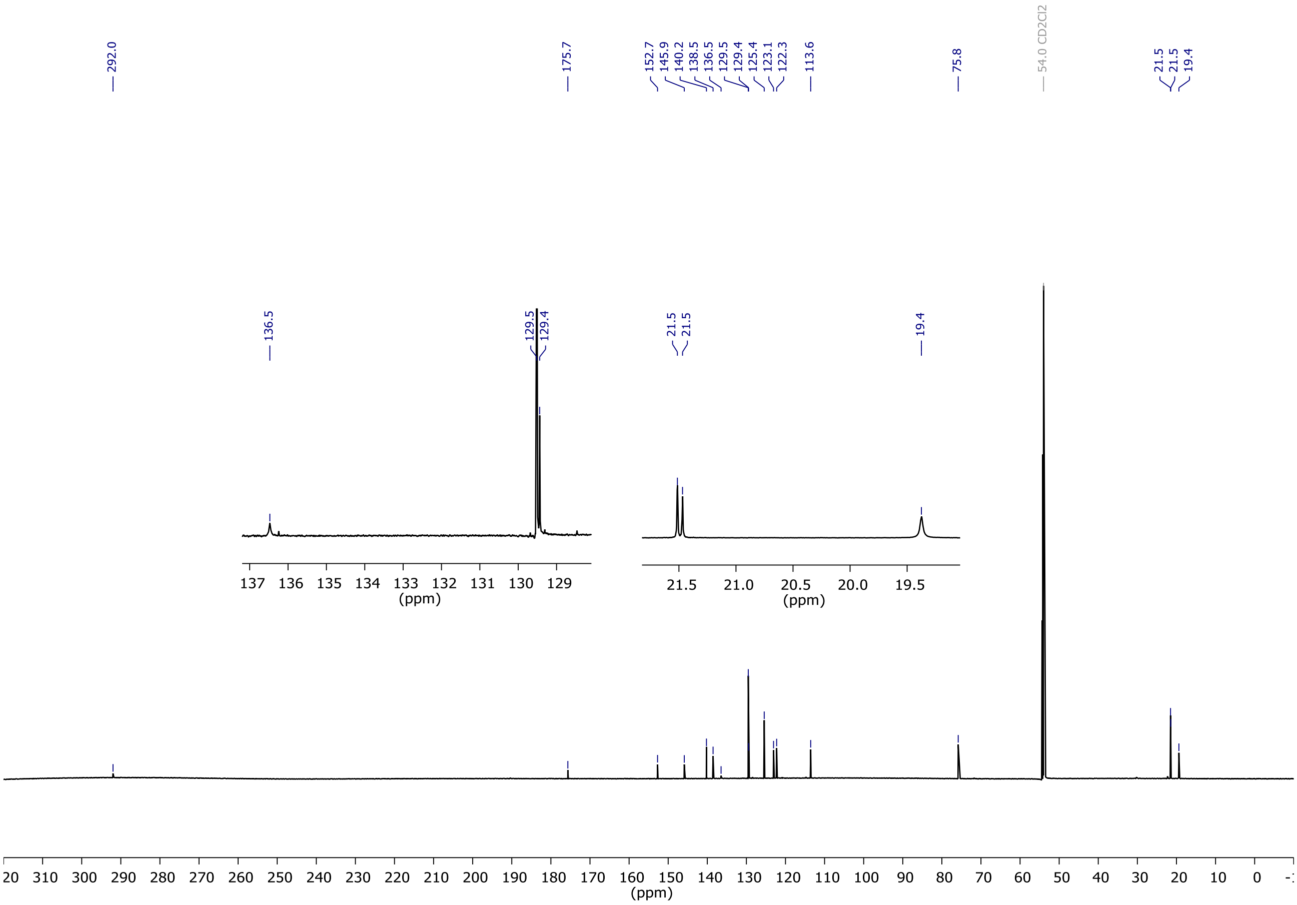
292.0
175.7
152.7
145.9
140.2
138.5
136.5
129.5
129.4
125.4
123.1
122.3
113.6
75.8
54.0 CD2Cl2
21.5
21.5
19.4
(ppm)

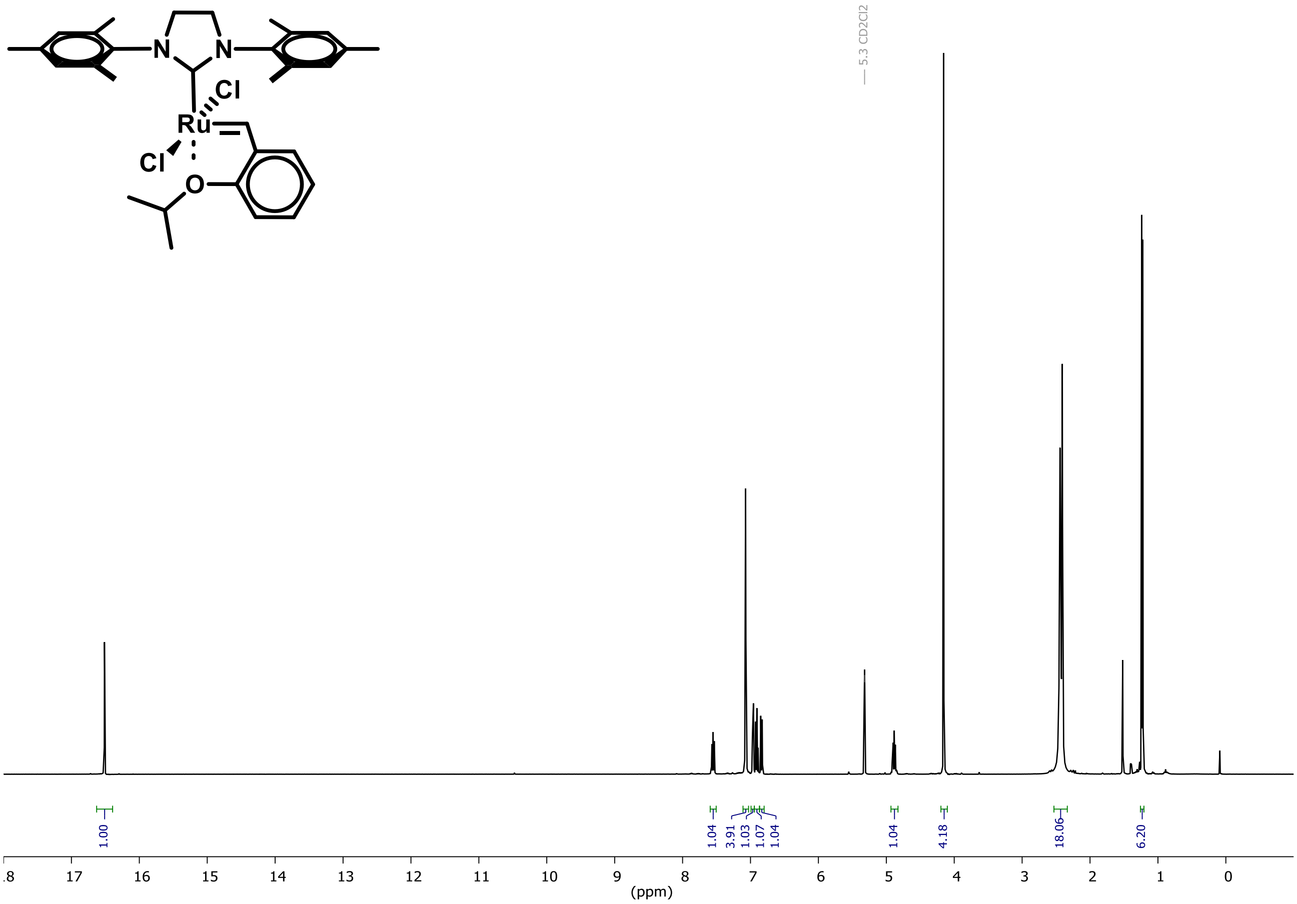
N
N
Cl
Ru
Cl
O
5.3 CD2Cl2
1.00
1.04
3.91
1.03
1.07
1.04
1.04
4.18
18.06
6.20
(ppm)

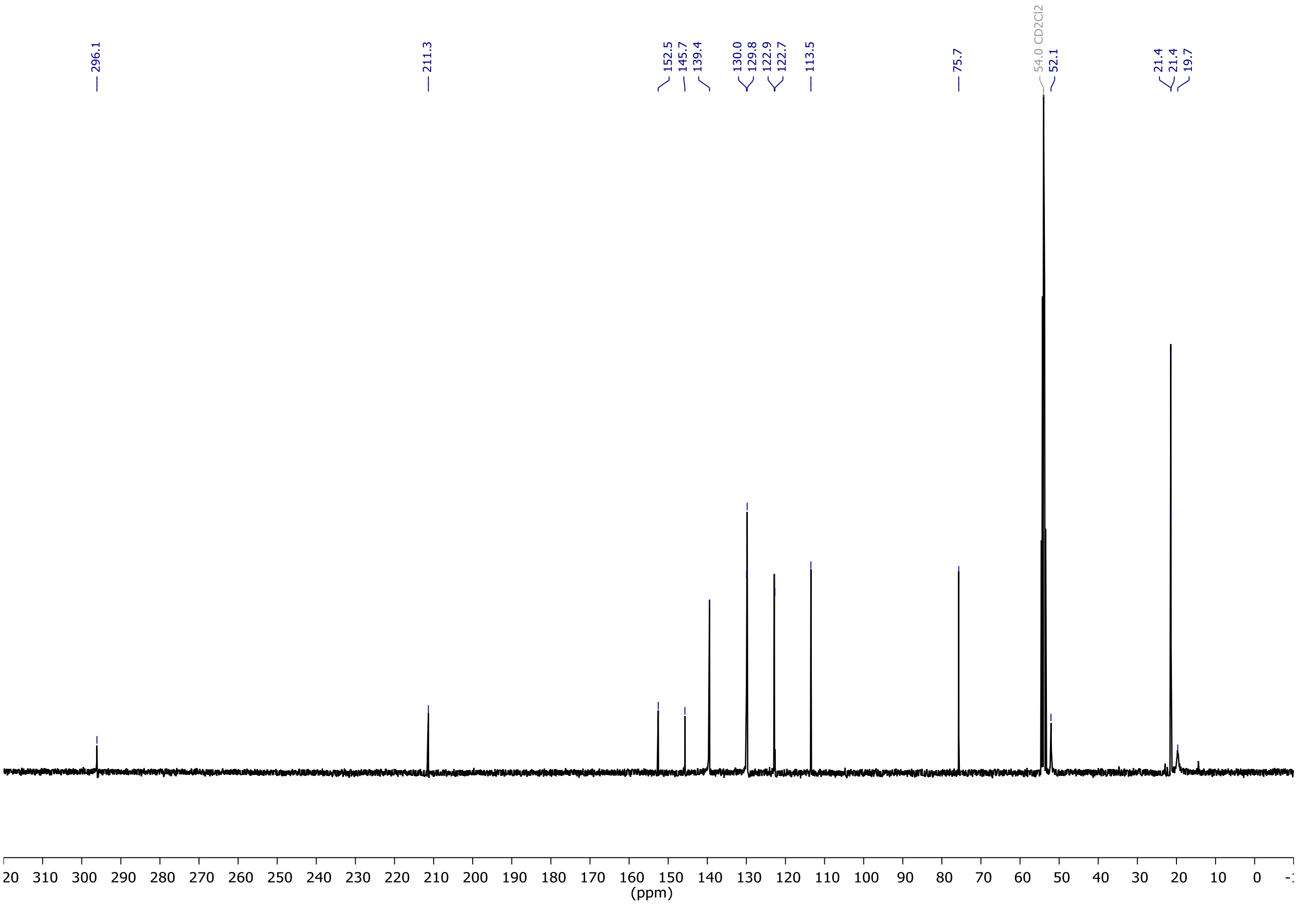
296.1
211.3
152.5
145.7
139.4
130.0
129.8
122.9
122.7
113.5
75.7
54.0 CD2Cl2
52.1
21.4
21.4
19.7
(ppm)

REFERENCES

1. Fulmer, G. R.; Miller, A. J. M.; Sherden, N. H.; Gottlieb, H. E.; Nudelman, A.; Stoltz, B. M.; Bercaw, J. E.; Goldberg, K. I., NMR Chemical Shifts of Trace Impurities: Common Laboratory Solvents, Organics, and Gases in Deuterated Solvents Relevant to the Organometallic Chemist. *Organometallics* **2010,** *29* (9), 2176-2179.
2. Otera, J.; Orita, A.; An, D.-L.; Zhang, Z.; Mineyama, H., Convenient Synthesis of (1-Propynyl)arenes through a One-Pot Double Elimination Reaction, and Their Conversion to Enynes. *Synlett* **2007,** *12*, 1909-1912.
3. Fürstner, A.; Seidel, G. Palladium-Catalyzed Arylation of Polar Organometallics Mediated by 9-Methoxy-9-Borabicyclo[3.3.1]nonane: Suzuki Reactions of Extended Scope. *Tetrahedron* **1995**, *51*, 11165-11176.
4. Masters, K.-S.; Wallesch, M.; Bräse, S., *ortho*-Bromo(propa-1,2-dien-1-yl)arenes: Substrates for Domino Reactions. *J. Org. Chem.* **2011,** *76* (21), 9060-9067.
5. Patchett, R.; Magpantay, I.; Saudan, L.; Schotes, C.; Mezzetti, A.; Santoro, F., Asymmetric Hydrogenation of Ketones with H2 and Ruthenium Catalysts Containing Chiral Tetradentate S2N2 Ligands. *Angew. Chem. Int. Ed.* **2013,** *52* (39), 10352-10355.
6. Ahmed, T. S.; Grandner, J. M.; Taylor, B. L. H.; Herbert, M. B.; Houk, K. N.; Grubbs, R. H., Metathesis and Decomposition of Fischer Carbenes of Cyclometalated Z-Selective Ruthenium Metathesis Catalysts. *Organometallics* **2018,** *37* (14), 2212-2216.
7. Van Veldhuizen, J. J.; Gillingham, D. G.; Garber, S. B.; Kataoka, O.; Hoveyda, A. H., Chiral Ru-Based Complexes for Asymmetric Olefin Metathesis: Enhancement of Catalyst Activity through Steric and Electronic Modifications. *J. Am. Chem. Soc.* **2003,** *125* (41), 12502-12508.
8. Kumar, G.; Guda, R.; Husain, A.; Bodapati, R.; Das, S. K., A Functional Zn(II) Metallacycle Formed from an N-Heterocyclic Carbene Precursor: A Molecular Sensor for Selective Recognition of Fe3+ and IO4– Ions. *Inorg. Chem.* **2017,** *56* (9), 5017-5025.
9. Nolan, S.; Meiries, S., A New Synthetic Route to p-Methoxy-2,6-disubstituted Anilines and their Conversion into N-Heterocyclic Carbene Precursors. *Synlett* **2013,** *25*, 393-398.
10. Kyan, R.; Sato, K.; Mase, N.; Watanabe, N.; Narumi, T., Tuning the Catalyst Reactivity of Imidazolylidene Catalysts through Substituent Effects on the N-Aryl Groups. *Org. Lett.* **2017,** *19* (10), 2750-2753.
11. Berlin, J. M.; Campbell, K.; Ritter, T.; Funk, T. W.; Chlenov, A.; Grubbs, R. H., Ruthenium-Catalyzed Ring-Closing Metathesis to Form Tetrasubstituted Olefins. *Org. Lett.* **2007,** *9* (7), 1339-1342.
12. Biberger, T.; Zachmann, R. J.; Fürstner, A., Grubbs Metathesis Enabled by a Light-Driven gem-Hydrogenation of Internal Alkynes. *Angew. Chem. Int. Ed.* **2020,** *59* (42), 18423-18429.
13. Day, C. S.; Fogg, D. E., High-Yield Synthesis of a Long-Sought, Labile Ru-NHC Complex and Its Application to the Concise Synthesis of Second-Generation Olefin Metathesis Catalysts. *Organometallics* **2018,** *37* (24), 4551-4555.
14. Castarlenas, R.; Esteruelas, M. A.; Oñate, E., N-Heterocyclic Carbene−Osmium Complexes for Olefin Metathesis Reactions. *Organometallics* **2005,** *24* (18), 4343-4346.
15. Merino, E.; Poli, E.; Díaz, U.; Brunel, D., Synthesis and characterization of new ruthenium N-heterocyclic carbene Hoveyda II-type complexes. Study of reactivity in ring closing metathesis reactions. *Dalton Trans.* **2012,** *41* (36), 10913-10918.
